# On the Reaction of Pacman‐Phosphanes with Lewis Acids

**DOI:** 10.1002/chem.202502029

**Published:** 2025-07-21

**Authors:** Leon Ohms, Pascal Schmidt, Jonas Surkau, Jonas Bresien, Axel Schulz

**Affiliations:** ^1^ Institut für Chemie Universität Rostock Albert‐Einstein‐Straße 3a 18059 Rostock Germany; ^2^ Leibniz‐Institut für Katalyse e.V. an der Universität Rostock Albert‐Einstein‐Straße 29a 18059 Rostock Germany

**Keywords:** adducts, borane, gacl3, pacman, phosphane

## Abstract

The reactions of Pacman phosphanes (**1a**) with Lewis acids such as R_3_B (R = C_6_H_5_, C_6_F_5_) and GaCl_3_ were investigated. While equilibria between mono‐ and di‐adducts were found in the reaction with BPh_3_, B(C_6_F_5_)_3_ preferentially formed the di‐adduct (**1a·**2B(C_6_F_5_)_3_), which could be isolated and fully characterized. density functional theory (DFT) calculations showed that the *N*‐bonded adducts are thermodynamically more stable than the *P*‐bonded adducts. In contrast to the reaction of the Pacman phosphane with boranes, GaCl_3_ reacted with **1a** to give an unusual [Pacman‐GaCl_2_]^+^[GaCl_4_]^−^ salt, featuring a nearly planar GaN_2_C_2_ heterocycle as part of the Pacman macrocycle. In addition, the coordination geometry on one P atom changes from triple to an unusual quadruple coordination, although it is still a formal P atom in the + III oxidation state, as shown by quantum chemical calculations.

## Graphical abstract

The reactivity of a Pacman phosphane (a macrocyclic, multidentate phosphane ligand) with Lewis acids such as BPh₃, B(C₆F₅)₃, and GaCl₃ were investigated, revealing distinct bonding motifs and coordination behaviors. While B(C₆F₅)₃ forms a stable di‐adduct, GaCl₃ induces an unusual structural rearrangement with the formation of a [Pacman‐GaCl₂]⁺[GaCl₄]⁻ salt and a rare tetracoordinated phosphorus center in the + III oxidation state.

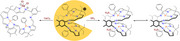

## 1 Introduction

**Scheme 1:**
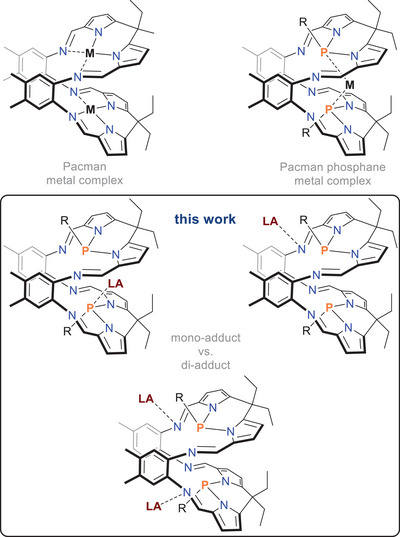
Top: Pacman metal complex^[^ ^20^ ^]^ (left), Pacman phosphane metal complex^[^ ^21^ ^]^ (right), and bottom: Pacman phosphane Lewis acid (LA) complexes.

Pacman ligands, first introduced in the late 1970s to mimic enzymes and study metal‐metal interactions, are a class of nitrogen‐based heterocycles widely used as chelating ligands in coordination chemistry (Scheme 1, top left).^[^ ^1^, ^2^ ^]^ The term “Pacman ligand” is a colloquial reference to their unique structural characteristics, which resemble the shape of the popular arcade character “Pac‐Man.”^[^ ^3^, ^4^ ^]^ These ligands are typically large, multidentate molecules that can wrap around or “capture” metal ions in a specific, constrained geometry, resembling Pac‐Man's open mouth. This ability to coordinate with (mostly) two metal centers in a precise and selective manner makes Pacman ligands particularly interesting in various applications, such as in catalysis, materials science, and bioinorganic chemistry.^[^ ^5^, ^6^ ^]^ Pacman ligands can be based on porphyrins or calix[4]pyrroles,^[^ ^7^, ^8^ ^]^ featuring mostly one metal ion in both halves of the multidentate Pacman ligand.

Only recently, Pacman phosphanes were introduced, which are a specific subclass of Pacman ligands that incorporate two phosphorus‐based donor atoms into their structure,^[^ ^9^, ^10^ ^]^ however, in contrast to classical diphosphanes without a direct P‐P bond (Scheme 1, top right).^[^ ^11^, ^12^, ^13^, ^14^, ^15^, ^16^, ^17^, ^18^, ^19^ ^]^ Phosphanes are well‐known in coordination chemistry for their ability to donate electron pairs to metal centers, forming stable metal‐phosphane complexes. What makes Pacman phosphanes distinctive is the way two phosphane groups are arranged within the ligand to create a rigid, “C‐shaped” structure that can enclose and “capture” a metal ion in a specific, sterically confined manner.^[^ ^9^ ^]^ Typically, Pacman phosphanes consist of two central, unlinked phosphane fragments, each attached to two nitrogen atoms of the Pacman heterocycle, which is structured to create a cavity around both P donor atoms. As a result, when Lewis acidic metal cations are incorporated into the Pacman system, they are usually coordinated by the two phosphorus atoms, but the iminic nitrogen atoms of the Pacman ligand can also act as additional donor atoms ^[^ ^9^ ^]^ Therefore, Pacman phosphanes can bind to metal ions through multiple donor sites. The phosphorus atoms provide electron density to the metal center, while the rest of the ligand's structure helps maintain a controlled and stable coordination environment.

We were intrigued by the idea of studying the reaction of Pacman phosphanes with neutral, classical main group element‐based Lewis acids (Scheme 1, bottom), focusing on the adduct formation that can occur via either the embedded central P atoms or the N atoms of the heterocycles. In addition, the formation of FLPs (frustrated Lewis acid‐base pair)^[^ ^22^, ^23^, ^24^ ^]^ is still possible if the steric demand is high enough, so that in this case a corresponding activation chemistry could be possible.

## 2 Results and Discussion

### 2.1 Synthesis of Pacman Phosphanes.^[^ ^9^ ^]^

**Scheme 2:**
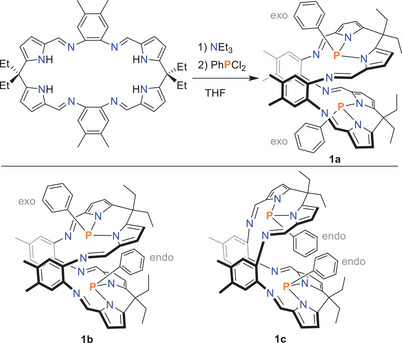
Synthesis of Pacman phosphanes **1**.

A yellow solution of Pacman ligand dissolved in tetrahydrofuran (THF) is treated with triethylamine at ambient temperature, then cooled down to − 80 °C. At this temperature, PhPCl_2_ is added dropwise, forming P‐N bonds in Pacman phosphane **1** as HCl is eliminated. After removal of the THF and extraction with diethyl ether, a mixture of the exo‐exo‐isomer (**1a**) and endo‐exo‐isomer (**1b**) is obtained. To separate the exo‐exo‐isomer (as shown in Scheme 2) from the endo‐exo‐isomer, the orange powder is dissolved in THF, concentrated in vacuo, and left overnight for crystallization at 5 °C, resulting in the deposition of orange crystals of the exo‐exo‐isomer with a yield of ca. 40%. Pure (exo‐exo) **1a** decomposes at 284 °C and shows a singlet ^31^P{^1^H} nuclear magnetic resonance (NMR) signal in CD_2_Cl_2_ at 68.1 ppm (s).

### 2.2 Reaction of Pacman Phosphane 1a with Phenyl Boranes

#### 2.2.1 Synthesis

**Scheme 3:**
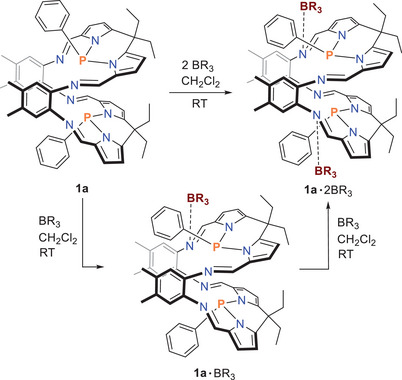
Reaction of **1a** with BR_3_ (R = Ph, C_6_F_5_).

**Figure 1:**
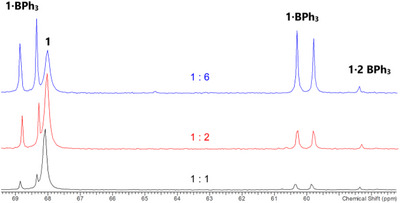
^31^P{^1^H} NMR spectrum of **1** : *n* BPh_3_ mixtures (*n* = 1–6).

The reaction was first carried out in an NMR tube, which was filled with **1a** and one equivalent of colorless BPh_3_ dissolved in deuterated dichloromethane, resulting in a clear yellow solution (Scheme 3). The ^31^P NMR spectrum revealed the formation of mono‐adduct **1**·BPh_3_ (δ[^31^P] = 68.6 and 60.2, doublet) and small amounts of di‐adduct **1**·2BPh_3_ (δ[^31^P] = 58.4, singlet), with uncoordinated **1a** (δ[^31^P] = 68.1, singlet) remaining the major component (Figure 1). After 24 hours another equivalent of BPh_3_ was added, and another 24 hours later, four additional equivalents of BPh_3_ were added. In both experiments, signals of all three species (**1a**, **1a**·BPh_3_, and **1**·2BPh_3_) were observed; however, mono‐adduct **1a**·BPh_3_ was now the dominant species, and there was a slightly increased intensity for **1a**·2BPh_3_.

Since in the reaction of **1a** with BPh_3_ only equilibria between **1a**, mono‐ and di‐adducts were found, we used a stronger Lewis acid, namely B(C_6_F_5_)_3_, in a second series of experiments to favor adduct formation. Indeed, when one or two or more equivalents of B(C_6_F_5_)_3_ were added, the formation of the novel di‐adduct (**1a**·2 B(C_6_F_5_)_3_, δ[^31^P] = 61.0, singlet) was observed in the ^31^P NMR experiment almost exclusively alongside traces of mono‐adduct (**1a**·2 B(C_6_F_5_)_3_, (δ[^31^P] = 72.1 and 21.0 doublet). Even when purified crystals of the di‐adduct were dissolved again, only very small traces of mono‐adduct were found, but no uncoordinated starting material (δ[^31^P] = 68.1). The optimized reaction of **1a** and B(C_6_F_5_)_3_ in CH_2_Cl_2_ yielded a highly pure, crystalline di‐adduct in yields between 50 and 60%, which could be isolated in the form of yellow crystals. **1a·**2B(C_6_F_5_)_3_ decomposes above 254 °C and dissolves completely in CH_2_Cl_2_, albeit with the formation of small amounts of mono‐adduct.

**Figure 2:**
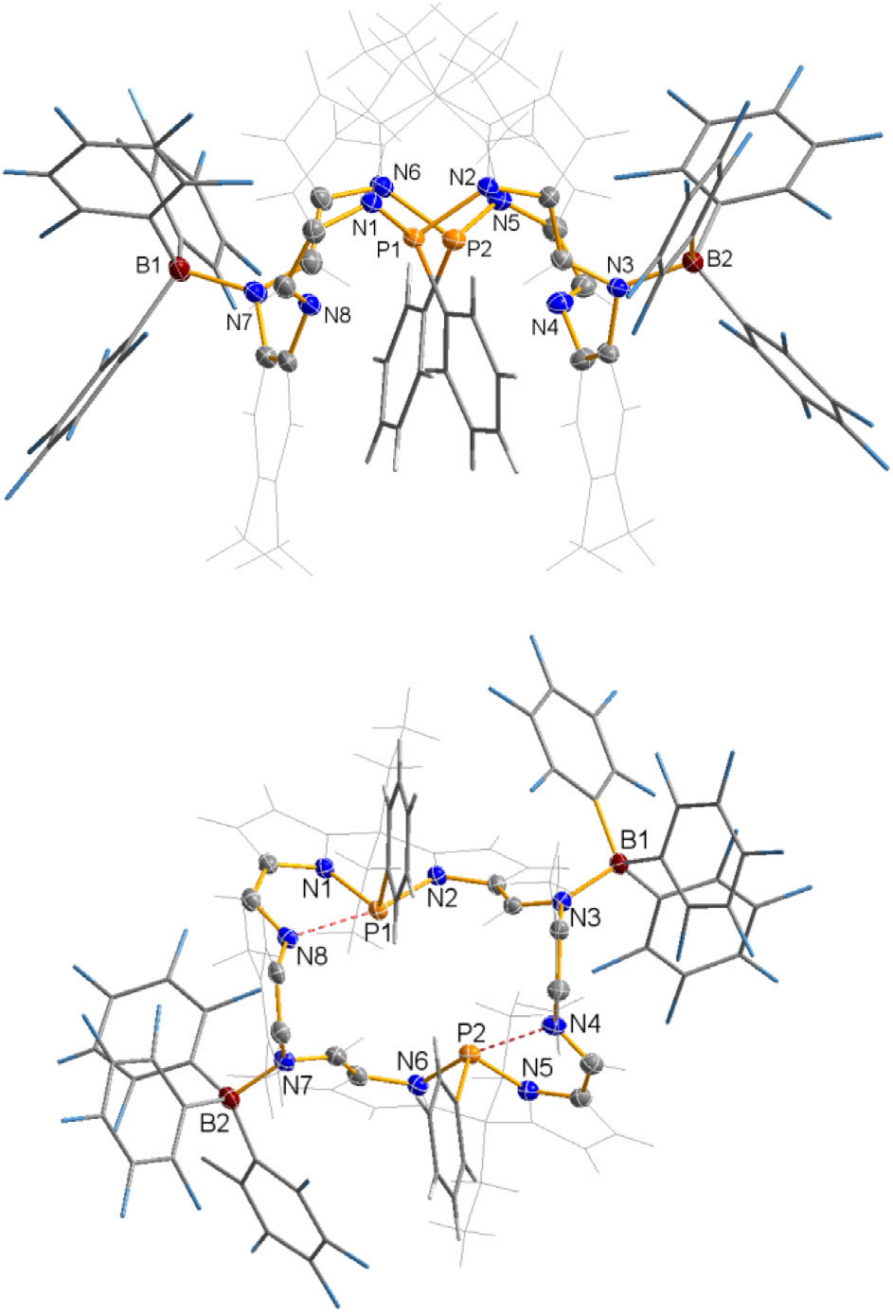
Two different views of the molecular structure of **1a·**2B(C_6_F_5_)_3_ in the crystal. ORTEPs are shown of all noncarbon ring atoms and ring carbon atoms (T = 123(2) K, ellipsoid probability = 50%). Color code: N blue, C grey, H light grey, P orange, F light blue, and B brown. Selected structural parameters, distances in Å: P1···P2 3.9433(9), P1‐N2 1.827(2), P2‐N6 1.814(2), P1‐N1 1.754(2), P2‐N5 1.750(2), P1···N8 2.499(2), P2···N4 2.496(2), B1‐N3 1.630(3), B2‐N7 1.637(3).

Single‐crystal X‐ray diffraction studies (SC‐XRD) clearly showed the presence of the di‐adduct **1a·**2B(C_6_F_5_)_3_ in solid state, but both Lewis acid molecules are bound to the two iminic N atoms of the Pacman ligand, but not to the P atoms of the two phosphane moieties (Figure 2). The typical Pacman structure as illustrated in Scheme 2 is maintained. **1a·**2B(C_6_F_5_)_3_ crystallizes in the monoclinic space group *P*2_1_/*c* with *Z* = 4. In accord with computations (vide infra), both B(C_6_F_5_)_3_ molecules are attached to iminic N atoms outside the “Pacman mouth.” The B‐N distances of 1.630(4) and 1.637(4) Å are in the expected range of *N*‐bound B(C_6_F_5_)_3_ donor‐acceptor complexes (cf. Σ*r*_cov_(B‐N)  =  1.56 Å,^[^ ^25^ ^]^ 1.556(2) and 1.564(2) in the silver dicyanamide salt [Ag(Et_2_O)_3_][N{CN·B(C_6_F_5_)_3_}_2_], 1.611(2) in HCN·B(C_6_F_5_)_3_)_2_, and N1–B1 1.664(2) in HN_3_·B(C_6_F_5_)_3_).^[^ ^26^, ^27^, ^28^, ^29^, ^30^ ^]^ The formation of the *P*‐bound adduct would lead to significantly longer P‐B bonds (Σ*r*_cov_(P‐B)  =  1.96 Å)^[^ ^25^ ^]^ and is thermodynamically less favored according to computations (see below). The distorted pyramidal P atom displays two P‐N bonds in the range of 1.75 – 1.83 Å which corresponds to a P‐N single bond (Σ*r*_cov_(P‐N) = 1.82 Å).^[^ ^25^ ^]^ The P‐N distances to the not directly bound N4 / N8 atoms lie both around 2.50 Å, which is considerably smaller than the sum of the van der Waals radii (Σ*r*_vdW_(P‐N) = 3.32 Å), indicative of weak interactions.^[^ ^31^ ^]^

Finally, we tried to use the borane‐Pacman adducts for the activation of small molecules such as CS_2_, EtBr, and C_2_H_2_, but in all these cases no activation was observed (see Supporting Information).

#### 2.2.2 Computations–Structure and Bonding

**Scheme 4:**
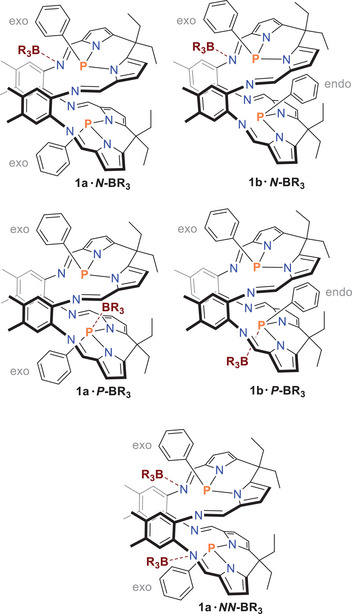
Structural sketch of the mono‐ and di‐adducts under consideration.

To shed light on the thermodynamics of the adduct formation, quantum chemical computations were carried out. As we encountered a variety of different isomers with a large number of conformers and rotamers within the Pacman‐phosphane macrocycle, we always conducted an isomer search calculation for each species first, employing the xTB software (GFN2‐xTB level of theory)^[^ ^32^, ^33^ ^]^ as well as the CREST computer code^[^ ^34^, ^35^ ^]^ and CENSO algorithm^[^ ^36^ ^]^ (for details, see Supporting Information) to better understand the complex 3D structure of **1** and its Lewis acid adducts. Only the lowest energy isomers/conformers are discussed below. Starting with the Pacman phosphane macrocycle, we have essentially found only two important isomers, namely the *exo‐exo* (**1a**) and *endo‐exo* species (**1b**), which are separated by only 2.4 kcal/mol, with **1a** being the most thermodynamically stable isomer (Scheme 2). Note: The *endo‐endo* species is optimized to the *exo‐exo* species **1a**. Starting from the isomers **1a** and **1b**, we have now calculated all possible mono‐ and di‐adducts (Scheme 4). For comparison, in addition to the BPh_3_ and B(C_6_F_5_)_3_ adducts, we also calculated BH_3_ adducts to account for the possible problem of steric hindrance within the Pacman phosphane.

**Scheme 5:**
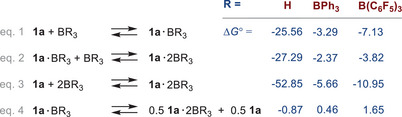
Computed thermodynamical data (in kcal/mol) of the mono‐ and di‐adduct formation in the reaction between **1a** and BR_3_ (level of theory: b97‐3c + SMD[CH_2_Cl_2_] + GmRRHO(GFN2[alpb]‐bhess) // b97‐3c[SMD], for details see Supporting Information). All adducts are *N*‐bonded, but the notation is omitted for clarity.

The relative stability of the *P*‐ and *N*‐bonded mono‐adducts of **1a** and **1b** (Table 1) shows that the *N*‐bonded adducts are thermodynamically much more stable in all cases, whereas in the *P*‐bonded adducts only the BH_3_ adduct is a true adduct, while in the BPh_3_ and B(C_6_F_5_)_3_ *P*‐bonded adducts the borane dissociates off to form a weakly bonded van der Waals complex with the Pacman phosphane, which can be attributed to the increased steric demand of these two boranes compared to the small BH_3_. In addition, the *exo‐exo* adducts **1a·** *N*‐BR_3_ are thermodynamically the most stable in all cases (Table 1). For this reason, to save CPU time, starting from **1a**, only the *NN*’‐bonded di‐adducts, **1a·** *NN*‐2BR_3_, were investigated further in the following. Stable **1a·** *NN*‐2BR_3_ isomers were found for all three boranes (see Supporting Information, Table S3). As expected, the formation of the mono‐adduct **1a**·*N*‐BR_3_ according to eq. 1 is exergonic for all three boranes, whereby the exergonicity along **1a**·*N*‐BH_3_ [‐25.56] > **1a**·*N*‐B(C_6_F_5_)_3_ [‐7.13] > **1a**·*N*‐BPh_3_ [‐3.29 kcal/mol] becomes significantly lower (Scheme 5). The same trend can also be observed in the formation of di‐adducts according to eqs. 2 and 3. Since the reaction enthalpies for adduct formation in the case of BPh_3_ and B(C_6_F_5_)_3_ are relatively small (between ca. −2 and −11 kcal/mol), it is not surprising that traces of mono‐adduct are always observed upon addition of two or more equivalents of Lewis acid or upon dissolution of di‐adduct crystals.

**Table 1:** Relative energies (in kcal/mol) of the *N*‐ and *P*‐bound mono‐adducts of **1a** and **1b**.

species/ R =	BH_3_	BPh_3_	B[C_6_F_5_]_3_
1a‐*N*‐BR_3_	0.00	0.00	0.00
1a‐*P*‐BR_3_	23.44	16.41^[^ ^a^ ^]^	20.48^[^ ^a^ ^]^
1b‐*N*‐BR_3_	1.63	7.14	6.76
1b‐*P*‐BR_3_	22.76	11.75^[^ ^a^ ^]^	15.29^[^ ^a^ ^]^

^[a]^ no true adduct since the B‐P distance is far too long

According to Natural Bond Orbital (NBO) analysis^[^ ^37^, ^38^, ^39^ ^]^ both P atoms possess two strongly polarized P‐N and one weakly polarized P‐C bonds along with one lone pair localized at each P atom (Table S9). The B‐N bonds are similarly polarized like the P‐N bonds, and the charge transfer amounts to 0.367e per B(C_6_F_5_)_3_ molecule.

### 2.3 Reaction of Pacman Phosphane 1a with GaCl_3_

#### 2.3.1 Synthesis

**Scheme 6:**
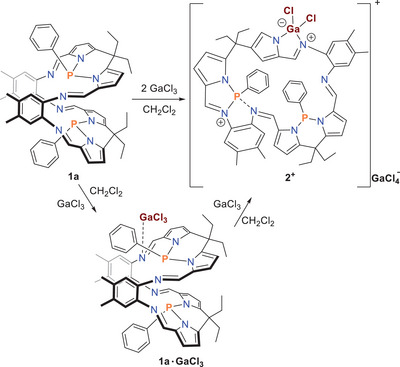
Reaction of **1a** with GaCl_3_.

In a third series of experiments, we studied the reaction of **1a** with the Lewis acid GaCl_3_ (Scheme 6). For this purpose, the orange Pacman ligand was dissolved together with colorless crystalline GaCl_3_ in dichloromethane, and the solution was stirred for 1 h at room temperature to form an orange solution. The solution was layered with *n*‐heptane. After 4 days, orange‐colored crystals could be isolated (yield 40%). The ^1^H, ^13^C, and ^31^P NMR data showed that it was not a classical di‐adduct as in **1a·**2B(C_6_F_5_)_3_ (vide supra), which was isolated, because two singlet signals were found in the ^31^P NMR spectra (δ[^31^P] = 70.3 and 52.2), and a fourfold splitting of the classical Pacman ring signals was observed in the ^1^H/^13^C spectra (see Figure S2). The isolated crystals (**2**GaCl_4_, Scheme 6) decomposed above 175 °C but are long‐term stable when stored under argon in a sealed glass ampoule.

**Figure 3:**
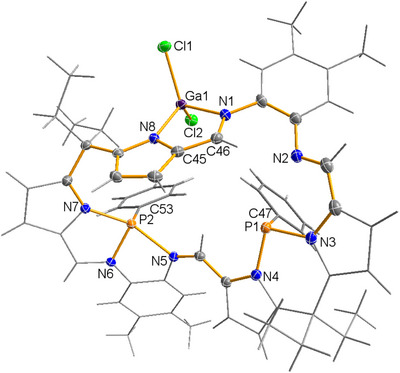
Molecular structure of **2**GaCl_4_ in the crystal. Only one of the two independent molecules is shown. [GaCl_4_]^−^ omitted for clarity. ORTEPs are shown of all noncarbon ring atoms and ring carbon atoms (T = 123(2) K, ellipsoid probability = 50%). Color code: N blue, C grey, H light grey, P orange, Cl green, and Ga violet. Selected structural parameters, distances in Å: P1···P2 5.385(1), P1‐N3 1.744(2), P1‐N4 1.782(2), P2‐N6 1.825(2), P2‐N7 1.855(2), P2‐N5 2.137(2), P1‐C47 1.826(3), P2‐C53 1.844(2), Ga1‐N8 1.914(2), Ga1‐N1 1.954(2), Ga1‐Cl2 2.1473(7), Ga1‐Cl1 2.1497(7), N1‐C46 1.321(3), N8‐C45 1.395(3), C45‐C46 1.400(3).

The exact molecular structure of the isolated crystals (**2**GaCl_4_) could be determined using SC‐XRD. **2**GaCl_4_ crystallized in the monoclinic space group *P*2_1_/*n* with *Z* = 8 and two independent molecules per cell. Only one of the two (very similar) independent molecules is depicted in Figure 3, revealing a macrocyclic cation (**2^+^**) featuring a GaN_2_C_2_ five‐membered heterocycle with [GaCl_4_]^−^ as counter ion. This means that presumably the two GaCl_3_ molecules formally reacted to give a [GaCl_2_]^+^ and [GaCl_4_]^−^, with the [GaCl_2_]^+^ ion now coordinating to an iminic N atom and a neighboring pyrrolic N atom that was previously bound to a P atom, forming a [Pacman‐GaCl_2_]^+^ cation (**2^+^**), featuring the GaN_2_C_2_ five‐membered heterocycle now as part of the Pacman‐phosphane and with the [GaCl_4_]^−^ ion as the counterion. It can be assumed that in the first step the mono‐adduct **1a**·GaCl_3_ is formed (Scheme 6), from which the second GaCl_3_ molecule abstracts a chloride ion, affording a cation that rearranges to give **2**GaCl_4_. It is worth noting that in solution, in addition to the main NMR signals in ^1^H, ^13^C, and ^31^P spectra that can be assigned to the structure of **2**GaCl_4_ as shown in Figure 3, another set of broadened signals can be observed (Figure S2). These signals also occur when pure crystals of **2**GaCl_4_ are dissolved, indicating a dynamic behavior of **2**GaCl_4_ in solution.

A closer look at the molecular structure in the crystal shows no significant interionic interactions and that the typical Pacman structure is no longer present (*d*(P1···P2) = 5.385(1), cf. 3.9433(9) Å in **1a**·B(C_6_F_5_)_3_), that is, it is only a macrocycle without the typical Pacman mouth with a formal 3‐coordinated and a 4‐coordinated P atom in the center of the overall cationic macrocycle. While the two P‐N bonds on the 3‐coordinated P1 atom (*d*(P1‐N3)  =  1.744(2), *d*(P1‐N4)  =  1.782(3) Å) are in the range of typical single bonds (vide supra), the three P‐N bonds on P2 are significantly elongated (*d*(P2‐N6)  =  1.825(2), *d*(P2‐N7)  =  1.856(2), *d*(P2‐N5)  =  2.137(3) Å, cf. Σ*r*_cov_(P‐N) = 1.82 Å,^[^ ^25^ ^]^ Σ*r*_vdW_(P‐N) = 3.32 Å).^[^ ^31^ ^]^ Therefore, the environment of P1 is best described as a distorted trigonal pyramid and that of P2 as a highly distorted bisphenoid (vide infra). Accordingly, the (N5‐P2‐N7) axial angle is 160.85(9)°, the (N6‐P2‐N7) angle is 83.49(9)° and the (N6‐P2‐C53) angle is 95.6(1)°. The five‐membered GaN_2_C_2_ heterocycle is almost planar (deviation from planarity < 5°) with Ga‐N distances of 1.914(2) and 1.954(2) Å (cf. Σ*r*_cov_(Ga‐N) = 1.95 Å).^[^ ^25^ ^]^

#### 2.3.2 Computations–Structure and Bonding

**Scheme 7:**
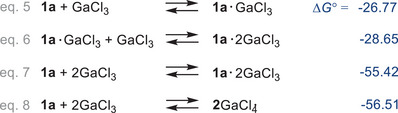
Computed thermodynamical data (in kcal/mol) for the reaction of **1a** with GaCl_3_ (Level of theory: b97‐3c + SMD[CH_2_Cl_2_] + GmRRHO(GFN2[alpb]‐bhess) // b97‐3c[SMD], for details see Supporting Information).

The same methodology as discussed before for the boranes was used to calculate the structures and thermodynamic data of the gallium species discussed below (for more details, see Supporting Information). As already calculated for the boranes, both the mono‐ (Δ_eq.5_ *G*° = −26.8/mol) and the di‐adduct formation (Δ_eq.7_ *G*° = −55.42 kcal/mol) between **1a** and GaCl_3_ are exergonic (Scheme 7). However, the formation of **2**GaCl_4_ is also exergonic (Δ_eq.8_ *G*° = −56.51 kcal/mol) and even more thermodynamically stable than the di‐adduct **1a**·2GaCl_3_ by −1.1 kcal/mol, which explains the formation of this salt and not the di‐adduct in the experiment (vide supra).

**Scheme 8:**
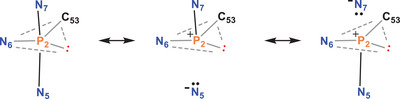
NBO Lewis formulae describing the 4‐electron 3‐center bond along the N5‐P2‐N7 axis in **2^+^**. Only the distorted pseudo‐trigonal bipyramidal environment around P2 is shown. A certain degree of ionic bonding can also be considered for the equatorial P‐N and P‐C bonds but is not shown here. The grey line represents only connectivity, and the black line a 2‐electron bond.

The generation of **2**GaCl_4_, with its unique structure around the phosphorus atoms and the GaN_2_C_2_ five‐membered heterocycle, warrants further investigation to better understand its structure and bonding. According to the NBO analysis, a lone pair is localized at each of the two P atoms, both of which have a dominant s‐character (P1: HO = h = sp^0.77^, P2: h = sp^0.58^; HO = h = hybrid orbital). While the NBO analysis finds two polarized N‐P bonds (with a N: 77 / P: 23% localization) and one weakly polarized P‐C bond (C: 62 / P: 38%) for P1, three very strongly polarized P2‐N bonds (P: 20, 15, and 8%) and one weakly polarized P‐C (P: 38%) bond are found for P2 (Tables S10). In agreement with the structural data (see above), this means that P1 adopts a distorted trigonal pyramidal structure, while P2 has a distorted bisphenoidal structure (Scheme 8). The unusual coordination geometry on the P2 requires further examination. A closer study of delocalization effects using second‐order perturbation theory within the NBO methodology shows very strong hyperconjugative effects, especially along the N‐P‐N axis [σ(P2‐N5) → σ*(P2‐N7): *E* ^2^  =  48 kcal/mol and σ(P2‐N7) → σ*(P2‐N5): *E* ^2^ = 81 kcal/mol], corresponding to resonance between covalent and ionic Lewis formulae (Scheme 8). In other words, the bond along the N‐P‐N axis in this pseudo‐trigonal bipyramidal structure is best understood as a classic 4‐electron 3‐center (4e, 3c) bonding unit, commonly observed in trigonal bipyramidal PX_5_ systems with P in the + V oxidation state. However, in Pacman phosphane cation **2^+^,** the P2 atom has a lone pair, indicating the formal oxidation state + III. Both the Ga‐N and the Ga‐Cl bonds are highly polarized, with only 18% (Ga‐Cl) and 11% (Ga‐N) localization of the bonds at the gallium center.

## 3 Conclusion

The reactions of Pacman phosphanes (**1a**) with Lewis acids such as R_3_B (R = C_6_H_5_, C_6_F_5_) and GaCl_3_ led to the formation of equilibria between free **1a**, mono‐ and di‐adducts. In the case of B(C_6_F_5_)_3_, it was possible to isolate the *N,N’*‐bonded di‐adduct (**1a**·2B(C_6_F_5_)_3_), which could be fully characterized. DFT calculations indicated that the *N*‐bonded adducts are thermodynamically more stable than the *P*‐bonded adducts.

In contrast to the reaction of the Pacman phosphane with boranes, GaCl_3_ reacted with **1a** to produce an intriguing [Pacman‐GaCl_2_]^+^[GaCl_4_]^−^ salt, featuring a nearly planar GaN_2_C_2_ heterocycle as part of the Pacman macrocycle. Additionally, the coordination geometry on one P atom changes from triple to an unusual quadruple coordination, although it remains a formal P atom in the + III oxidation state, as demonstrated by quantum chemical calculations. The formation of [Pacman‐GaCl_2_]^+^[GaCl_4_]^−^ salt is thermodynamically favored over the di‐adduct **1a**·2GaCl_3_ by 1.1 kcal/mol, consistent with experimental results.

## 4 Experimental Section

### General

All manipulations were carried out under an argon atmosphere using standard Schlenk line or Glovebox techniques (oxygen‐and moisture‐free conditions). All starting materials, reactants, and solvents were purified and/or synthesized according to literature procedures, which are described in further detail in the Supporting Information. The removal of solvents in vacuo was carried out at 1 × 10^−3^ mbar and at 25 °C if not stated otherwise. Further information on experimental procedures, data acquisition and processing, purification of starting materials and solvents, as well as a full set of analytical data for each compound and crystallographic information can be found in the Supporting Information.

### Synthesis of **1a**·2B(C_6_F_5_)_3_

A 25 mL Schlenk flask is filled with colorless B(C_6_F_5_)_3_ (215 mg, 0.42 mmol) and orange **1a** (217 mg, 0.21 mmol). The solids are dissolved in dichloromethane (12 mL) and left to stand without stirring overnight, resulting in the formation of yellow crystals. The supernatant solution is removed with a syringe and kept as a second fraction. The crystals are rinsed with dichloromethane (0.5 mL) at − 80 °C. The crystals are dried in vacuo (1 × 10^−3^ mbar) for 2 hours at 60 °C. The solution of the second fraction is concentrated in vacuo (1 × 10^−3^ mbar) and left to stand in a water bath overnight at 5 °C. The crystals of the second fraction are rinsed twice with dichloromethane (0.5 mL) at − 80 °C and dried in vacuo (1 × 10^−3^ mbar) for 2 hours at 60 °C. Yield: 223 mg (0.11 mmol, 55%).

**Mp**.: 254 °C (dec.); **EA** calc. (incl. 0.5 eq. CH_2_Cl_2_) (found) in %: C 56.88 (56.19), H 2.98 (2.92), N 5.62 (5.66); **^31^P{^1^H} NMR** (CD_2_Cl_2_, 202.5 MHz): *δ*  =  61.0 ppm (s); **MS**: (ESI, m/z, CH_2_Cl_2_): 929.4 [PacPPh]^+^.*

### Synthesis of **2**GaCl_4_

A 10 mL Schlenk tube is filled with orange crystalline **1a** (520 mg, 0.50 mmol) and colorless crystalline GaCl_3_ (180 mg, 1.02 mmol). The solids are dissolved in dichloromethane (10 mL). The solution is stirred for 1 hour at ambient temperature, resulting in an orange solution. The solution is layered with *n*‐heptane (5 mL) and left to stand for 4 d, resulting in the formation of orange crystals. The supernatant solution is removed via a syringe. The crystals are rinsed with dichloromethane (0.5 mL) at − 80 °C and dried in vacuo (1 × 10^−3^ mbar) for 1 hour. Yield: 254 mg (0.2 mmol, 40%).

**Mp**.: 176 – 187 °C (dec.); **EA** calc. (found) in %: C 54.37 (53.83), H 4.56 (4.90), N 8.75 (8.83); **^31^P{^1^H} NMR** (CD_2_Cl_2_, 202.5 MHz): *δ*  =  70.3 (s ppm (s, 1 P), 52.2 (s, 1 P); **MS**: (ESI, m/z, CH_2_Cl_2_): 1069.3 [K]^+^, 929.4 [PacPPh]^+^.*

* The full set of analytical data including assignments etc. can be found in the Supporting Information.

## Conflict of Interest

The authors declare no conflict of interest.

## Supporting information



Supporting Information

## Data Availability

The data that support the findings of this study are available in the supplementary information of this article.
