# Supplementary material for: On the Reaction of Pacman‐Phosphanes with Lewis Acids
Source: Chemistry. 2025 Jul 21;31(44):e202502029. doi: 10.1002/chem.202502029 (PMC12336754; doi:10.1002/chem.202502029)
Supplement: Supplementary file 1 — Supporting Information [file CHEM-31-e202502029-s001.pdf]

SUPPORTING INFORMATION

On the Reaction of Pacman-Phosphanes with Lewis Acids

*Leon Ohms, Pascal Schmidt, Jonas Surkau, Jonas Bresien, and Axel Schulz**

This file includes:

1 Experimental 2

2 Structure elucidation 4

3 Syntheses of compounds 7

4 Synthesis of non-isolated products 20

5 Activation attempts 22

6 Computational details 24

7 XYZ Structure of the "best" Isomer 29

8 References 83

1 Experimental

General information. If not stated otherwise, all manipulations were carried out under oxygen- and moisture-free conditions in an inert argon atmosphere using standard Schlenk or drybox techniques. All glassware was heated three times *in vacuo* using a heat gun (650 °C) and cooled under argon atmosphere. Solvents were transferred using syringes, which were purged three times with argon prior to use. Solvents and reactants were either obtained from commercial sources or synthesized as detailed in Table S1.

Table S1. Origin and purification of solvents and reactants.

Substance	Origin	Purification
CH_2Cl_2	local trade	purified according to literature procedure[1]<br>dried over P_4O_{10}, stored over CaH_2<br>freshly distilled and degassed (freeze-pump-thaw)
THF	Fisher Scientific, 99.5%	dried over Na/benzophenone<br>freshly distilled prior to use
n-hexane	local trade	dried over Na/benzophenone/tetraglyme<br>freshly distilled prior to use
toluene	local trade	dried over Na/benzophenone<br>freshly distilled prior to use
PhF	local trade	dried over CaH_2<br>freshly distilled prior to use
CD_2Cl_2	euriso-top	dried over P_4O_{10} and CaH_2<br>freshly distilled prior to use
NEt_3	Sigma Aldrich, 99%	dried over Na<br>freshly distilled prior to use
$PhBCl_2$	TCI, 98.0%	used as obtained
$GaCl_3$	old stock	-
$B(C_6F_5)_3$	synthesized[2]	purified according to literature procedure[2]
Pacman ligand	synthesized[3]	purified according to literature procedure[3]
PacPh	synthesized[3]	purified according to literature procedure[3]
$PacK_4$	synthesized[3]	purified according to literature procedure[3]

NMR spectra were recorded on Bruker spectrometers (AVANCE 250, AVANCE 300 or AVANCE 500) and were referenced internally to the deuterated solvent (^{13}C: CD_2Cl_2 δ_{ref} = 54.0 ppm), to protic impurities in the deuterated solvent (^{1}H: $CHDCl_2$ δ_{ref} = 5.32 ppm) or externally (^{11}B: $BF_3 \cdot Et_2O$ δ_{ref} = 0 ppm; ^{15}N: CH3NO2 δref = 0 ppm; ^{31}P: 85% H_3PO_4 δ_{ref} = 0 ppm). The ^{15}N chemical shifts were taken from ^{1}H-^{15}N-HMBC NMR spectra. All measurements were carried out at ambient temperature unless denoted otherwise. NMR signals were assigned using experimental data (e.g. chemical shifts, coupling constants, integrals where applicable).

IR spectra of crystalline samples were recorded on a Bruker Alpha II FT-IR spectrometer equipped with an ATR unit at ambient temperature under argon atmosphere. Relative intensities are reported according to the following intervals: very weak (vw, 0–10%), weak (w, 10–30%), medium (m, 30–60%), strong (s, 60–90%), very strong (vs, 90–100%).

Raman spectra of crystalline samples were recorded using a LabRAM HR 800 Horiba Jobin YVON Raman spectrometer equipped with an Olympus BX41 microscope with variable lenses. The samples were excited by an infrared laser (785 nm, 100 mW, air-cooled diode laser) or a red laser (633 nm, 17 mW, air-cooled HeNe laser). All measurements were carried out at ambient temperature unless stated otherwise.

Elemental analyses were obtained using an Elementar vario Micro cube CHNS analyser.

Melting points (uncorrected) were determined using a Stanford Research Systems EZ Melt at a heating rate of 20 °C/min. Clearing points are reported.

DSC analyses were carried out at a heating rate of 5 °C/min using a Mettler-Toledo DSC 823e.

Mass spectra were recorded on an Advion Expression L benchtop mass spectrometer (*m/z* 10–2000) equipped with an Advion Expression CMS detector using sample solutions.

2 Structure elucidation

X-ray structure determination: X-ray quality crystals were selected in Fomblin YR-1800 perfluoroether (Alfa Aesar) at ambient temperature. The samples were cooled to 123(2) K during measurement. The data were collected on a Bruker D8 Quest diffractometer using Mo K_α radiation (λ = 0.71073 Å). The structures were solved by iterative methods (SHELXT)[4] and refined by full matrix least squares procedures (SHELXL).[5] Semi-empirical absorption corrections were applied (SADABS).[6] All non-hydrogen atoms were refined anisotropically, hydrogen atoms were included in the refinement at calculated positions using a riding model. The unit cells of compound **1a**·2$B(C_6F_5)_3$ and **2**$GaCl_4$ contain solvent molecules which have been treated as a diffuse contribution to the overall scattering without specific atom positions by SQUEEZE/PLATON. These were removed from the model but included in the empirical formula.

Table S2. Crystallographic details.

Compound	**1a**·2B(C_6F_5)$_3$	**2**$GaCl_4$
Chem. Formula	$C_{94}H_{58}B_2F_{30}N_8P_2 \cdot 4\ CH_2Cl_2$	$C_{58}H_{58}Cl_6Ga\ N_8P_2 \cdot 0.25\ (CH_2Cl_2)$
Formula weight [g/mol]	2292.76	1299.97
Colour	yellow	orange
Crystal system	monoclinic	monoclinic
Space group	$P2_1/c$	$P2_1/n$
a [Å]	14.456(2)	29.735(2)
b [Å]	32.340(3)	13.1430(12)
c [Å]	22.283(2)	30.456(3)
α [°]	90	90
β [°]	105.160(3)	91.986(4)
γ [°]	90	90
V [Å^3]	10055(2)	11895(2)
Z	4	8
$\rho_{calcd.}$ [g/cm^3]	1.514	1.452
μ [mm^{-1}]	0.363	1.287
T [K]	123(2)	123(2)
Measured reflections	363630	545299
Independent reflections	21960	28705
Reflections with $I > 2\sigma(I)$	15659	20891
R_{int}	0.0839	0.1219
$F(000)$	3952	5239
$R_1(R[F^2>2\sigma(F^2)])$	0.0532	0.0387
w$R_2(F^2)$	0.1538	0.0957
GooF	1.039	1.014
No. of Parameters	1233	1414
CCDC #	2249841	2249842

Compound	**PacmanBPhOBu**
Chem. Formula	$C_{66}H_{76}B_2Cl_2N_8O_2$
Formula weight [g/mol]	1105.86
Colour	orange
Crystal system	monoclinic
Space group	$P2_1/c$
a [Å]	23.421(2)
b [Å]	12.1257(11)
c [Å]	21.7352(19)
α [°]	90
β [°]	104.658(2)
γ [°]	90
V [Å^3]	5971.7(9)
Z	4
$\rho_{calcd.}$ [g/cm^3]	1.230
μ [mm^{-1}]	0.16
T [K]	123(2)
Measured reflections	108101
Independent reflections	10521
Reflections with $I > 2\sigma(I)$	5553
R_{int}	0.148
$F(000)$	2352
$R_1(R[F^2>2\sigma(F^2)])$	0.078
$wR_2(F^2)$	0.253
GooF	1.06
No. of Parameters	861
CCDC #	2249843

3 Syntheses of compounds

3.1 Pacman phosphane tris(pentafluorophenyl)borane adduct 1a·2B(C_6F_5)$_3$

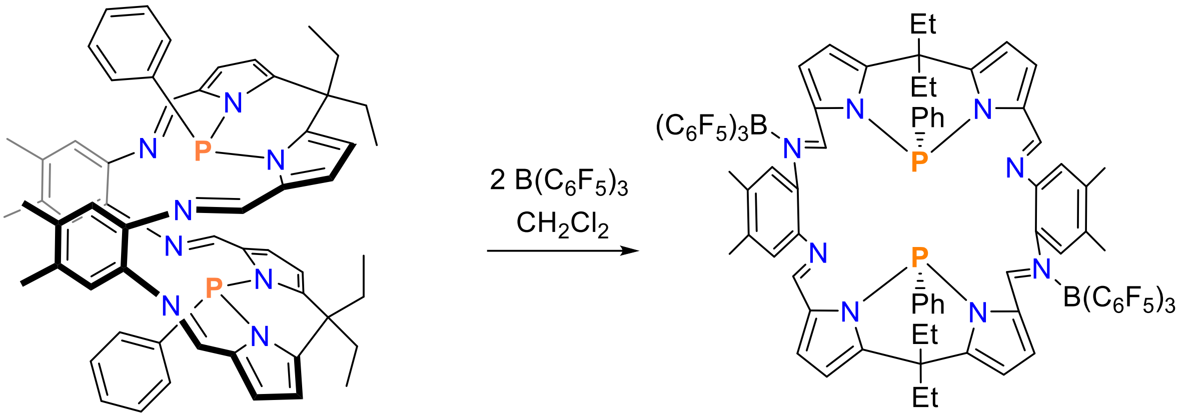

A 25 mL Schlenk flask is filled with colourless B(C_6F_5)$_3$ (215 mg, 0.42 mmol) and orange **1a** (217 mg, 0.21 mmol). The solids are dissolved in dichloromethane (12 mL) and left to stand without stirring overnight, resulting in the formation of yellow crystals. The supernatant solution is removed with a syringe and kept as a second fraction. The crystals are rinsed with dichloromethane (0.5 mL) at −80 °C. The crystals are dried *in vacuo* (1×10^{-3} mbar) for 2 h at 60 °C. The solution of the second fraction is concentrated *in vacuo* (1×10^{-3} mbar) and left to stand in a water bath overnight at 5 °C. The crystals of the second fraction are rinsed twice with dichloromethane (0.5 mL) at −80 °C and dried *in vacuo* (1×10^{-3} mbar) for 2 h at 60 °C. Yield: 223 mg (0.11 mmol, 55%).

In the NMR spectra, alongside the signals of the reported di-adduct, additional broadened signals of a putative mono-adduct which is formed in an equilibrium reaction can be observed.

Mp.: 254 °C (dec.); **EA** calc. (incl. 0.5 eq. CH_2Cl_2) (found) in %: C 56.88 (56.19), H 2.98 (2.92), N 5.62 (5.66); **^{31}P{^{1}H} NMR** (CD_2Cl_2, 202.5 MHz): δ = 61.0 ppm (s); **^{1}H NMR**

(CD_2Cl_2, 500.1 MHz) δ = 9.03 (br s, 2 H, iminic C*H* with borane), 7.55 (d, *J* = 2.3 Hz, 2 H; iminic C*H* without borane), 7.45 (t, $^3J(^1H,^1H)$ = 7.4 Hz, 2 H, phenyl *para*-C*H*), 7.10 (t, $^3J(^1H,^1H)$ = 7.4 Hz, 4 H, phenyl *meta*-C*H*), 6.97 (d, $^3J(^1H,^1H)$ = 4.0 Hz. 2 H, pyrrolic C*H* near imin without borane), 6.77 (t, $^3J(^1H,^1H)$ = 4.6 Hz, 2 H, porrolic C*H* near imin with borane), 6.58 - 6.67 (m, 4 H, phenyl *ortho*-C*H*), 6.48 (d, $^3J(^1H,^1H)$ = 4.0 Hz, 2 H, pyrrolic C*H* near Et-groups without borane), 6.27 (s, 2 H, aromatic C*H* with borane), 6.03 (d, $^3J(^1H,^1H)$ = 4.6 Hz, 2 H, pyrrolic C*H* near Et-groups with borane), 5.39 (s, 2 H, aromatic C*H* without borane), 2.28 - 2.39 (m, 2 H, *exo*-R-CH*H*-CH_3 without borane), 2.06 (s, 6 H, Ar-CH_3 without borane), 2.01 (m, 2 H, *exo*-R-C*H*H-CH_3 with borane), 1.94 (s, 6 H, Ar-CH_3 with borane), 1.71 - 1.82 (m, 4 H, *endo*-R-CH_2-CH_3), 0.60 (t, $^3J(^1H,^1H)$ = 7.2 Hz, 6 H, *exo*-R-CH_2-CH_3) 0.21 ppm (t, $^3J(^1H,^1H)$ = 7.3 Hz, 6 H, *endo*-R-CH_2-CH_3); **$^{19}F\{^1H\}$ NMR (**CD_2Cl_2, 470.6 MHz) δ = −123.4 (m,1 F, *ortho*-C*F*), −125.5 (m, 1 F, *o*-C*F*), −126.4 (m, 1 F, *ortho*-C*F*), −130.8 (m, 1 F, *ortho*-C*F*), −133.5 (br s, *otho*-C*F* of presumed mono adduct), −134.3 (m, 1 F, *ortho*-C*F*), −135.9 (m, 1 F, *ortho*-C*F*), −158.8 (br t, $^3J(^{19}F, ^{19}F)$ = 21 Hz, 1 F, *para*-C*F*), −159.8 (br t, $^3J(^{19}F, ^{19}F)$ = 21 Hz, 1 F, *para*-C*F*), −159.7 (br t, $^3J(^{19}F, ^{19}F)$ = 20 Hz, *para*-C*F* of presumed mono adduct), −159.9 (br t, $^3J(^{19}F, ^{19}F)$ = 21 Hz, 1 F, *para*-C*F*), −164.8 - −164.3 (m, 1 F, *meta*-C*F*), −164.8 - −164.3 (m, 1 F, *meta*-C*F*), −165.9 - −165.5 (m, 1 F, *meta*-C*F*), −165.9 - −165.5 (m, *meta*-C*F* of presumed mono adduct), −166.1 (m, 1 F, *meta*-C*F*), -166.4 (m, 1 F, *meta*-C*F*), −166.9 ppm (m, 1 F, *meta*-C*F*); **$^{13}C\{^1H\}$ NMR** (CD_2Cl_2, 125.76 MHz): δ = 150.6 (s, pyrrolic *C*-C-Et_2 with borane), 150.3 (br s, iminic *C*H without borane), 144.7 (s, pyrrolic *C*-C-Et_2 without borane), 141.0 (s, aromatic *C* with borane), 139.6 (s, aromatic *C* without borane), 137.1 (s, aromatic *C*-CH_3 with borane), 135.0 (s, pyrrolic *C* without borane), 131.7 (s, aromatic *C*-CH_3 without borane), 131.1 (s, phenyl *para*-*C*H), 130.7 (s, phenyl *ortho*-*C*H), 129.9 (br s, pyrrolic *C*H near imin with borane), 129.5 (br s, phenyl *meta*-*C*H), 129.3 (s, aromatic *C*H with borane), 125.8 (s, pyrrolic *C* with borane), 124.3 (s, aromatic *C*H without borane), 122.2 (s, pyrrolic *C*H near imin without borane), 111.3 (s, pyrrolic *C*H near Et-groups with borane), 110.8 (s, pyrrolic *C*H near Et-groups without borane), 46.3 (s, *C*-Et_2), 43.1 (s, *endo*-*C*H_2-CH_3), 35.4 (s, *exo*-*C*H_2-CH_3), 19.0 (s, Ar-*C*H_3), 19.0 (s, Ar-*C*H_3), 9.5 (s, *exo*-CH_2-*C*H_3) 9.2 ppm (br s,

endo-CH_2-CH_3);); **^{15}N NMR** (CD_2Cl_2, 50.7 MHz): δ = − 87.4 (s, iminic *N* without borane), − 194.3 (pyrrolic *N* with borane), − 229.8 ppm (pyrrolic *N* without borane); **^{11}B NMR (**CD_2Cl_2, 128.3 MHz) δ = −7.3 ppm (br s, *B*); **IR** (ATR, 32 scans, cm^{-1}): $\tilde{\nu}$ = 2882 (w), 2923 (w), 2941 (w), 2935 (w), 2966 (w), 600 (w), 608 (w), 589 (w), 837 (w), 653 (w), 1179 (w), 1193 (w), 503 (w), 1344 (w), 1350 (w), 1235 (w), 575 (w), 470 (w), 1643 (w), 462 (m), 519 (m), 629 (m), 872 (m), 884 (m), 1482 (m), 1377 (m), 1039 (m), 410 (m), 449 (m), 420 (m), 486 (m), 936 (m), 851 (m), 1573 (m), 1622 (m), 1410 (m), 783 (m), 773 (m), 742 (m), 563 (m), 1515 (s), 1315 (s), 676 (s), 1280 (s), 695 (s), 1084 (vs), 1451 (vs), 975 cm^{-1} (vs); **Raman** (633 nm, 10 s, 20 Scans, cm^{-1}): $\tilde{\nu}$ = 3150 (2), 3125 (2), 3095 (2), 3063 (2), 3030 (2), 2990 (2), 2978 (2), 2970 (2), 2967 (2), 2953 (2), 2936 (2), 2927 (2), 2891 (2), 2880 (2), 2861 (2), 1870 (2), 1648 (3), 1621 (10), 1608 (5), 1577 (6), 1511 (4), 1501 (3), 1487 (4), 1459 (3), 1413 (8), 1399 (5), 1387 (3), 1370 (3), 1344 (3), 1318 (4), 1314 (5), 1289 (4), 1275 (6), 1265 (5), 1242 (4), 1226 (3), 1208 (3), 1192 (3), 1179 (4), 1161 (3), 1137 (3), 1101 (3), 1086 (4), 1077 (3), 1058 (3), 1038 (5), 1026 (3), 1002 (4), 980 (5), 966 (4), 937 (4), 908 (3), 886 (3), 874 (3), 869 (3), 852 (3), 835 (4), 800 (3), 788 (3), 767 (3), 754 (3), 745 (3), 739 (3), 733 (3), 707 (3), 698 (3), 682 (3), 678 (3), 672 (2), 660 (2), 651 (3), 641 (3), 629 (3), 619 (3), 610 (3), 591 (3), 584 (5), 566 (3), 548 (3), 541 (3), 513 (2), 494 (4), 477 (3), 473 (3), 467 (3), 449 (4), 437 (3), 421 (3), 403 (3), 391 (3), 373 (3), 357 (3), 350 (3), 328 (3), 323 (3), 309 (3), 299 (3), 288 (4), 278 (3), 239 (3), 222 (3), 200 (3), 188 cm^{-1} (3); **MS:** (ESI$^+$, m/z, CH_2Cl_2): 929.4 [PacPPh]$^+$.

Suitable crystals for single crystal X-ray crystallography were received as described above.

Figure S1. NMR, IR and Raman spectra of **1a**·2B(C_6F_5)$_3$ (solvent signals indicated by asterisks).

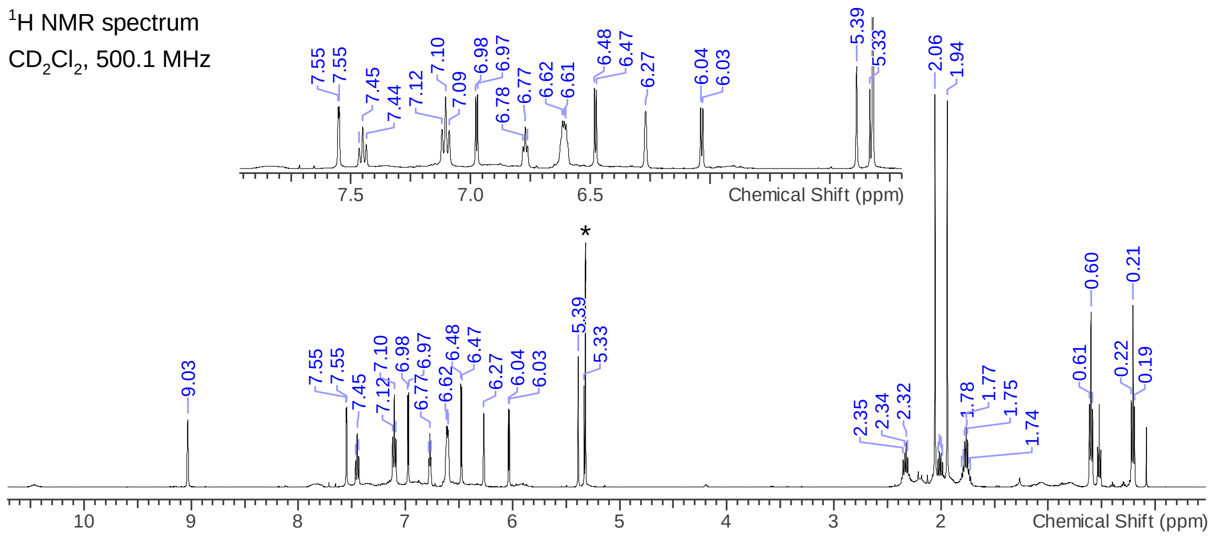

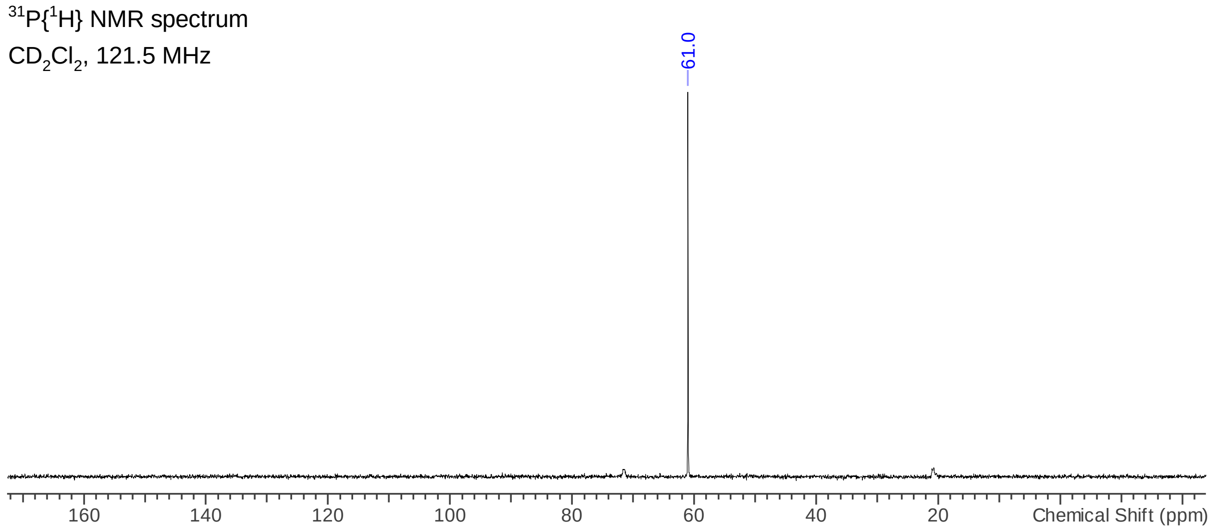

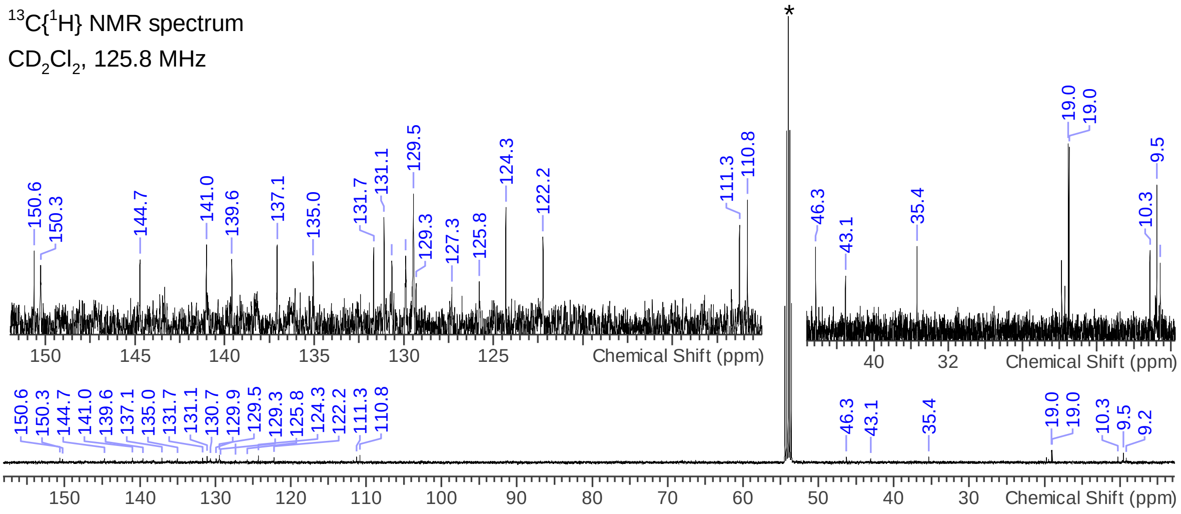

Figure S1 continued.

$^{19}F\{^{1}H\}$ NMR spectrum
CD_2Cl_2, 470.6 MHz

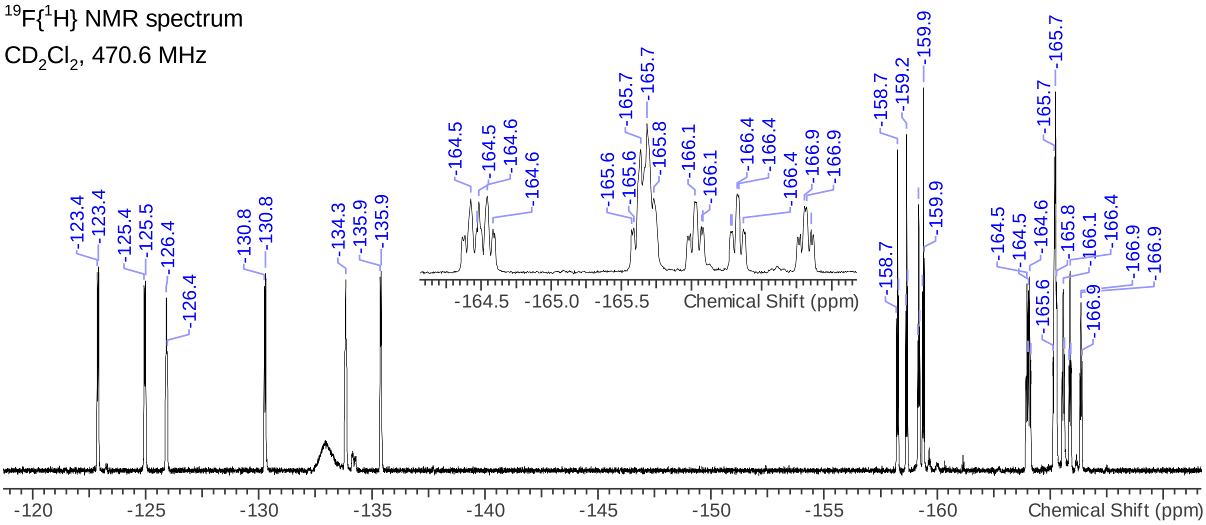

$^{11}B\{^{1}H\}$ NMR spectrum
CD_2Cl_2, 128.3 MHz

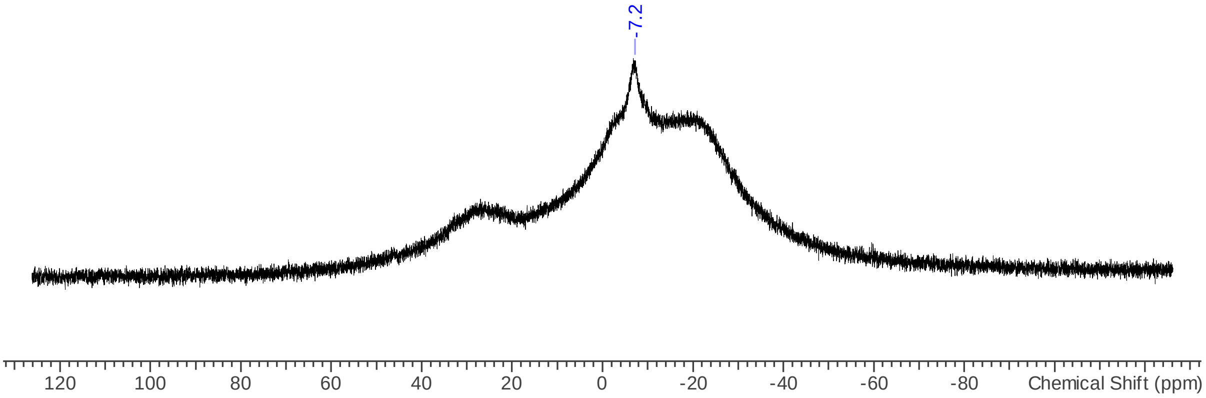

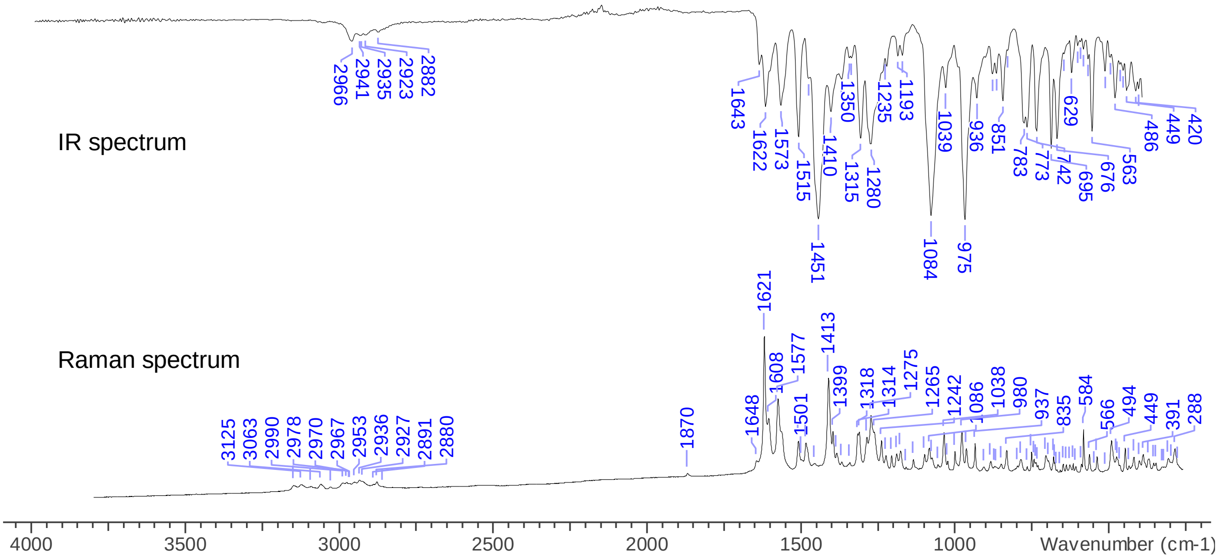

3.2 Pacman phosphane $GaCl_3$ adduct 2GaCl4

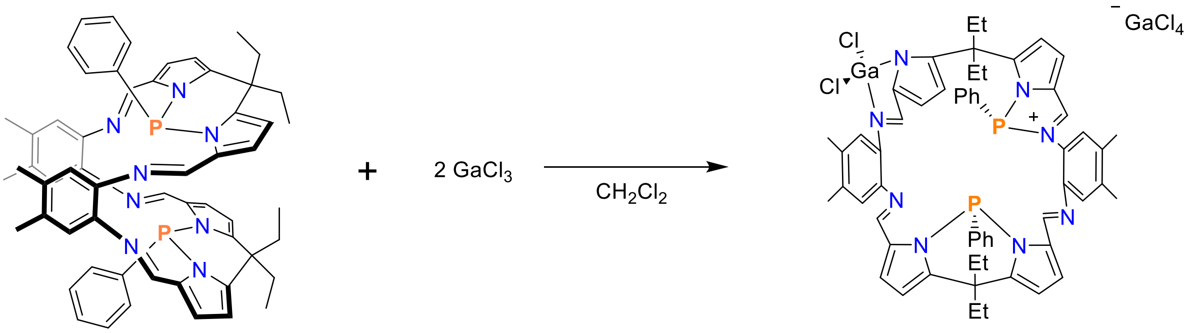

A 10 mL Schlenk tube is filled with orange crystalline **1a** (520 mg, 0.50 mmol) and colourless crystalline $GaCl_3$ (180 mg, 1.02 mmol). The solids are dissolved in dichloromethane (10 mL). The solution is stirred for 1 h at ambient temperature, resulting in an orange solution. The solution is layered with *n*-heptane (5 mL) and left to stand for 4 d, resulting in the formation of orange crystals. The supernatant solution is removed via a syringe. The crystals are rinsed with dichloromethane (0.5 mL) at −80 °C and dried *in vacuo* (1×10^{-3} mbar) for 1 h. Yield: 254 mg (0.2 mmol, 40%).

In the NMR spectra of the redissolved crystals of **2**$GaCl_4$, additional broadened signals can be observed alongside the signals of **2**$GaCl_4$. These are thought to form during dynamic processes of **2**$GaCl_4$ in solution.

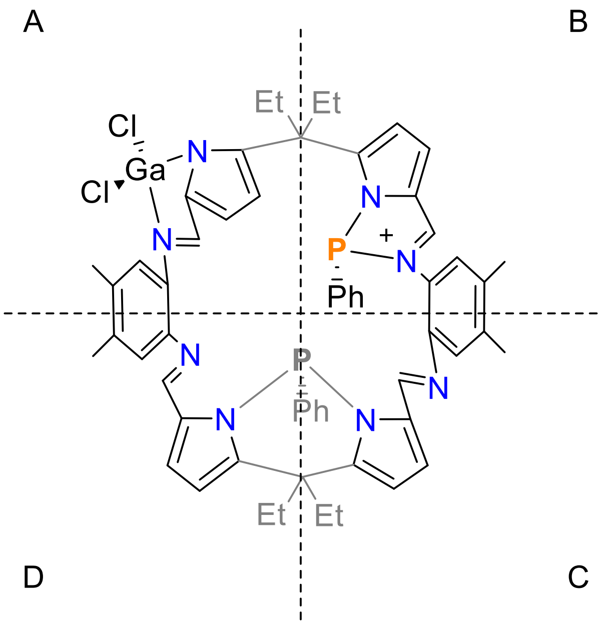

Mp.: 176 – 187 °C (dec.); **EA** calc. (found) in %: C 54.37 (53.83), H 4.56 (4.90), N 8.75 (8.83); **^{31}P{^{1}H} NMR** (CD_2Cl_2, 202.5 MHz): δ = 70.3 (s ppm (s, 1 P), 52.2 (s, 1 P); **^{1}H NMR (**CD_2Cl_2, 500.1 MHz): δ = 8.99 (d, J = 2.1 Hz, 1 H, iminic C*H* quadrant A), 8.93 (d, J = 4.1 Hz, 1H, iminic C*H* quadrant B), 8.52 (dd, J = 8.8, J = 1.8 Hz, 1H, iminic C*H* quadrant C), 8.40 (s, 1 H, iminic C*H* quadrant D), 7.63 (s, 1 H, aromatic C*H* quadrant A), 7.61 (dd, ^{3}J(^{1}H. ^{1}H) = 4.3 Hz, J = 3.2 Hz, 1 H, pyrrolic C*H* near imin quadrant B), 7.52 (d, ^{3}J(^{1}H, ^{1}H) = 4.3 Hz, 1 H, pyrrolic C*H* near imin quadrant C), 7.34 (s, 1 H, aromatic C*H* quadrant B), 7.29 – 7.33 (m, overlapping signals of phenyl groups), 7.24 (m, 1 H, pyrrolic C*H* near imin quadrant D), 7.15 – 7.25 (m, overlapping signals of phenyl groups), 7.14 (d, ^{3}J(^{1}H, ^{1}H) = 3.8 Hz, 1 H, pyrrolic C*H* near imin quadrant A), 7.13 (s, 1 H, aromatic C*H* quadrant C), 6.97 (d, ^{3}J(^{1}H, ^{1}H) = 4.3 Hz, 1 H, pyrrolic C*H* near Et-groups quadrant B), 6.64 (d, ^{3}J(^{1}H, ^{1}H) = 3.8 Hz, 1 H, pyrrolic C*H* near Et-groups quadrant D), 6.61 (s, 1 H, aromatic C*H* quadrant D), 6.44 (d, ^{3}J(^{1}H, ^{1}H) = 4.3 Hz, 1 H, pyrrolic C*H* near Et-groups quadrant C), 6.07 (d, ^{3}J(^{1}H, ^{1}H) = 3.8 Hz, 1 H, pyrrolic C*H* near Et-groups quadrant A), 2.30 (s, 3 H, Ar-CH_3 quadrant A), 2.27 (s 3 H, Ar-CH_3 quadrant B), 2.24 (s, 3 H, Ar-CH_3 quadrant D), 2.13 – 2.22 (m, 2 H, *exo*-R-CH_2-CH_3 half CD), 2.13 – 2.22 (m, 2 H, *endo*-R-CH_2-CH_3 half AB), 2.10 (s, 3 H, Ar-CH_3 quadrant C), 1.98 (m, 2 H, *endo*-R-CH_2-CH_3 half CD), 1.22 – 1.33 (m, 2 H, *exo*-R-CH_2-CH_3 half AB), 0.98 (t, ^{3}J(^{1}H. ^{1}H) = 7.3 Hz, 3 H, *exo*-R-CH_2-CH_3 half CD) 0.90 (t, ^{3}J(^{1}H, ^{1}H) = 7.3 Hz, 3 H, *endo*-R-CH_2-CH_3 half AB), 0.58 (t, ^{3}J(^{1}H, ^{1}H) = 7.5

Hz, 3 H, *endo*-R-CH_2-CH_3 half CD), 0.35 (t, $^3J(^1H, ^1H)$ = 7.2 Hz, 3 H, *exo*-R-CH_2-CH_3 half AB); **^{13}C{^{1}H} NMR** (CD_2Cl_2, 125.8 MHz): δ = 158.29 (s, quaternary *C*), 157.1 (d, *J* = 5 Hz, iminic *C*H quadrant A), 156.2 (s, quaternary *C*), 154.0 (s, quaternary *C*), 150.9 (d, *J* = 3 Hz, iminic *C*H quadrant D), 148.1 (s, quaternary *C*), 143.2 (d, *J* = 6 Hz, iminic *C*H quadrant C), 142.6 (s, iminic *C*H quadrant B), 140.6 (s, quaternary *C*), 139.1 (s, quaternary *C*), 138.0 (s, quaternary *C*), 137.4 (s, quaternary *C*), 136.7 (s, quaternary *C*), 135.2 (s, quaternary *C*), 134.9 (m, quaternary *C*), 134.0 (d, *J* = 7 Hz, quaternary *C*), 132.5 (s, quaternary *C*), 132 (d, *J* = 24 Hz, phenyl *C*H), 131.7 (s, quaternary *C*), 131.5 (s, phenyl *C*H), 131.2 (s, phenyl *C*H), 130.8 (d, *J* = 23 Hz, phenyl C*H*), 129.8 (d, *J* = 7 Hz, phenyl *C*H), 129.5 (d, *J* = 8 Hz, phenyl *C*H), 128.0 (s, quaternary *C*), 126.3 (s, aromatic *C*H quadrant A), 125.8 (s, pyrrolic *C*H near imin quadrant A), 125.8 (s, pyrrolic *C*H near imin quadrant D), 124.3 (s, pyrrolic *C*H near imin quadrant B), 123.6 (d, *J* = 7 Hz, pyrrolic *C*H near Et-groups quadrant B), 122.7 (s, aromatic *C*H quadrant D), 121.9 (s, pyrrolic *C*H near imin quadrant C), 120.4 (s, pyrrolic *C*H near et-groups quadrant A), 120.4 (s, aromatic *C*H quadrant C), 118.3 (s, aromatic *C*H quadrant B), 112.4 (s, pyrrolic *C*H near Et-groups quadrant C), 111.9 (s, pyrrolic *C*H near Et-groups quadrant D), 48.3 (d, *J* = 2 Hz, *C*-Et_2 half AB), 46.2 (s, *C*-Et_2 half CD), 42.4 (s, *endo*-CH_2-CH_3 half CD), 36.2 (s, *exo*-CH_2-CH_3 half CD), 31.4 (s, *endo*-CH_2-CH_3 half AB), 29.9 (m, *exo*-CH_2-CH_3 half AB), 20.5 (s, Ar-CH_3 quadrant B), 20.0 (s, Ar-CH_3 quadrant C), 19.7 (s, Ar-CH_3 quadrant A), 19.7 (s, Ar-CH_3 quadrant D), 10.7 (s, *exo*-CH_2-CH_3 half CD), 10.1 (s, *endo*-CH_2-CH_3 half AB), 9.6 (s, *endo*-CH_2-CH_3 half CD), 8.5 ppm (s, *exo*-CH_2-CH_3 half CD); **^{15}N NMR** (CD_2Cl_2, 50.7 MHz): δ = −89.0 (s, iminic *N* quadrant D), −132.3 (s, iminic *N* quadrant C), −170.9 (s, pyrrolic *N* quadrant B), −191.4 (s, iminic *N* quadrant A), −201.6 (s, pyrrolic *N* quadrant A), −213.9 (s, pyrrolic *N* quadrant C), −238.0 (s, pyrrolic *N* quadrant D); **IR** (ATR, 32 scans, cm^{-1}): $\tilde{\nu}$ = 2958 (m), 2935 (m), 2873 (m), 1595 (s), 1548 (s), 1517 (m), 1503 (m), 1480 (m), 1451 (s), 1437 (s), 1404 (m), 1393 (m), 1379 (m), 1358 (w), 1344 (w), 1325 (m), 1288 (s), 1266 (s), 1241 (vs), 1214 (s), 1187 (s), 1160 (m), 1134 (m), 1103 (m), 1080 (s), 1062 (vs), 1041 (s), 1002 (s), 963 (m), 944 (m), 911 (s), 901 (m), 880 (m), 864 (m), 851 (m), 843 (m), 829 (s), 794 (s), 777 (s), 742 (s), 734 (s), 721 (m), 690 (s), 668 (m), 645 (m), 620 (m), 589 (m), 569 (w), 546

(s), 515 (m), 488 (s), 470 (m), 447 (m), 433 (s), 418 cm^{-1} (s); **Raman** (633 nm, 5 s, 10 Scans, cm^{-1}): $\tilde{\nu}$ = 3125 (2), 3118 (2), 3098 (2), 2974 (2), 2941 (2), 2919 (2), 1893 (2), 1827 (2), 1797 (2), 1763 (2), 1601 (6), 1576 (6), 1556 (5), 1503 (9), 1483 (6), 1455 (10), 1442 (7), 1407 (3), 1392 (5), 1341 (7), 1323 (3), 1306 (3), 1293 (4), 1269 (10), 1247 (5), 1212 (3), 1193 (3), 1177 (3), 1134 (2), 1124 (2), 1104 (2), 1092 (2), 1078 (2), 1062 (3), 1044 (5), 1001 (2), 969 (3), 947 (2), 910 (2), 883 (2), 867 (2), 841 (2), 828 (3), 789 (3), 741 (2), 722 (2), 713 (2), 668 (2), 657 (2), 646 (3), 637 (2), 623 (2), 619 (2), 590 (2), 577 (3), 548 (1), 522 (2), 505 (2), 491 (2), 477 (2), 469 (1), 450 (1), 431 (2), 421 (1), 399 (2), 367 (2), 363 (1), 342 (2), 319 (2), 290 (1), 253 (1), 233 cm^{-1} (2); **MS:** (ESI^+, m/z, CH_2Cl_2): 1069.3 $[K]^+$, 929.4 $[PacPPh]^+$.

Suitable crystals for single crystal X-ray crystallography were received as described above.

Figure S2. NMR, IR and Raman spectra of **2**$GaCl_4$ (solvent signals indicated by asterisks).

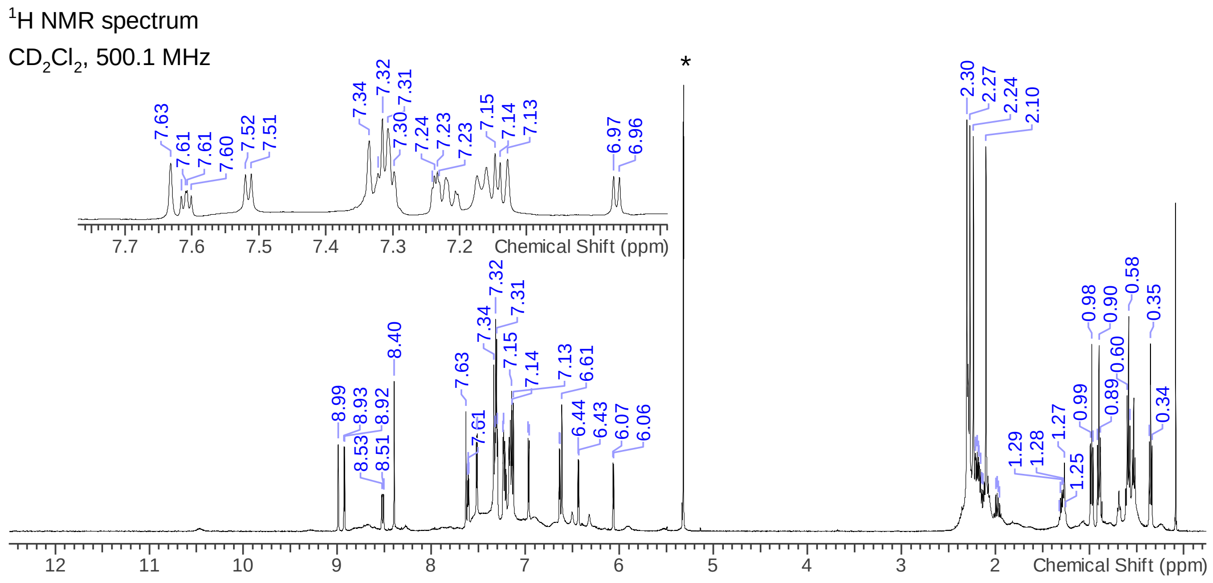

Figure S2 continued.

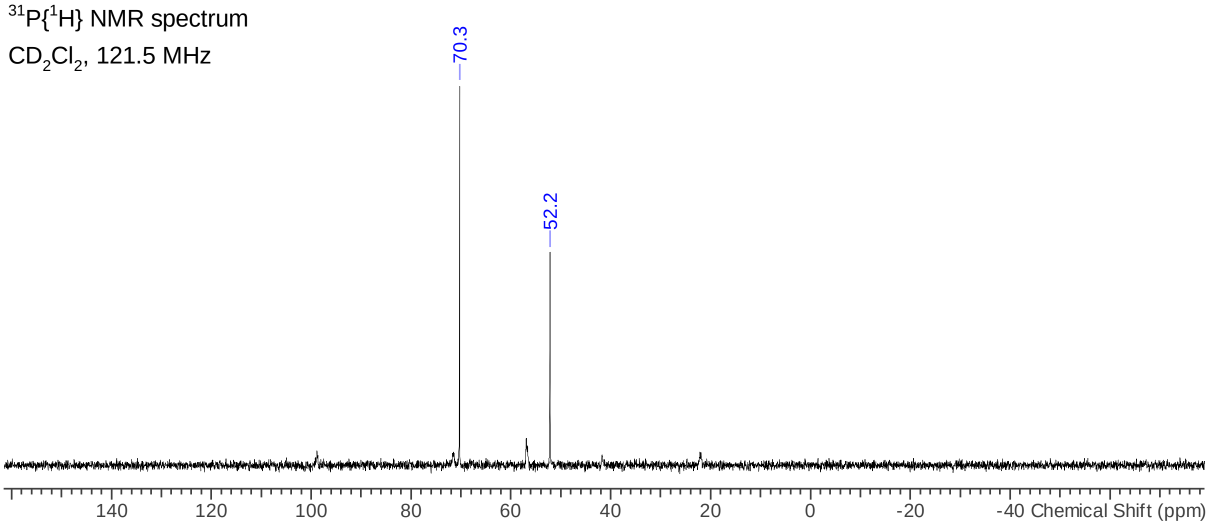

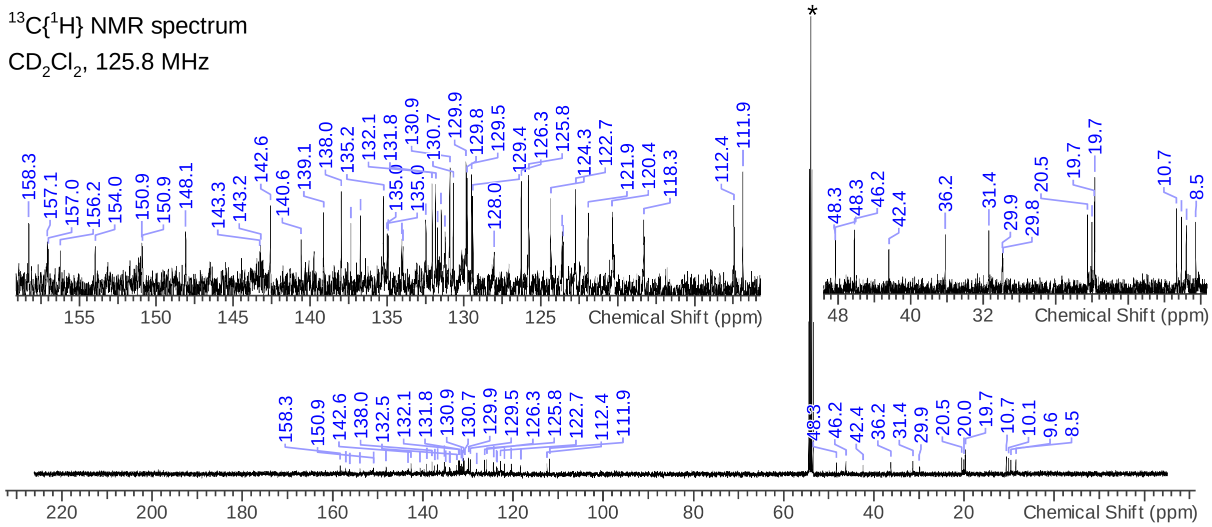

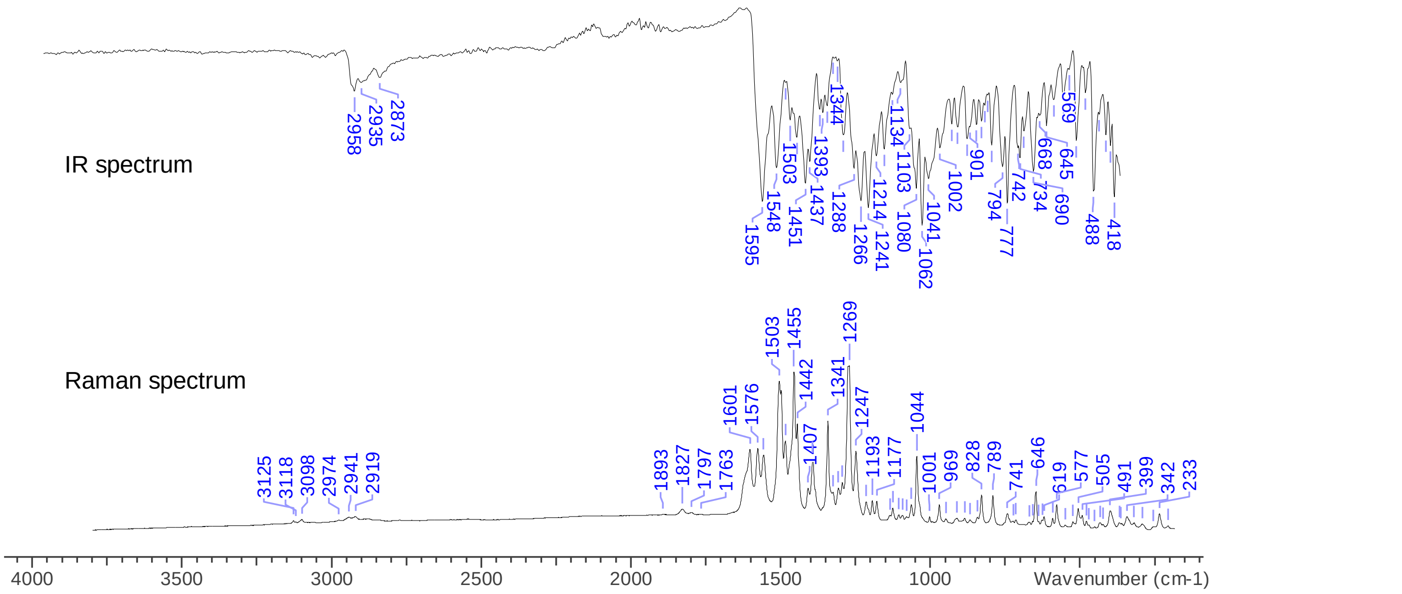

3.3 Pacman $PhBCl_2$ adduct PacBPhOBu

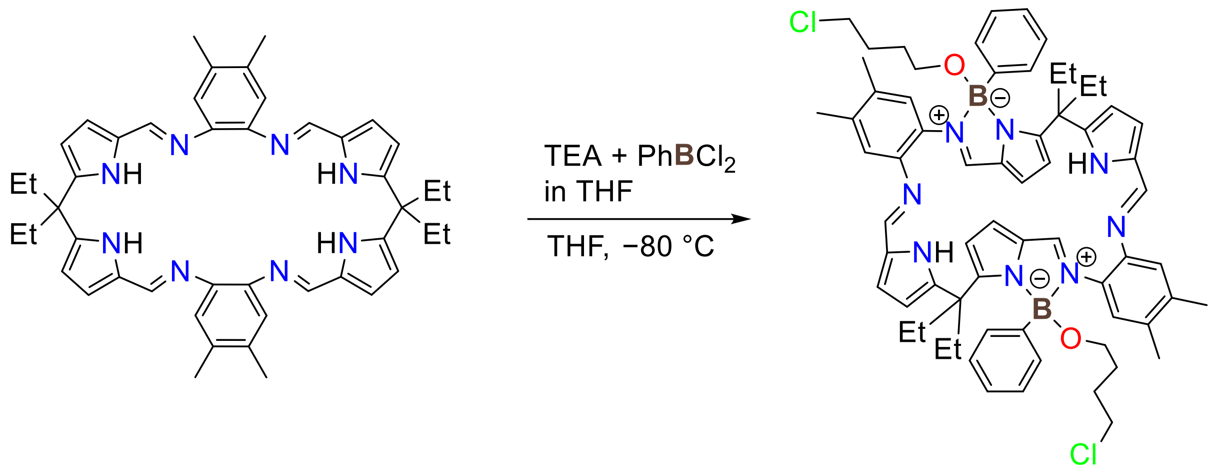

Pacman ligand **Pac** (472.0 mg, 0.558 mmol) is dissolved in THF (20 mL) giving an orange/red solution and is subsequently cooled to −80 °C in an isopropanol/nitrogen bath. In another flask $PhBCl_2$ (220 mg, 1.38 mmol) is added to TEA (0.3 mL, 2 mmol). The mixture is transferred into a syringe and THF (15 mL) is added to the mixture in the syringe. The mixture is mixed by swaying and added dropwise to the solution of **Pac** in THF over 10 min, leading to a color change to red. The solution is stirred for 30 min at −80 °C and subsequently allowed to slowly warm to RT while stirring continues overnight. Volatiles of the resulting orange/red solution are removed *in vacuo* (1×10^{-3} mbar) and the residue dried *in vacuo* (50 °C, 1×10^{-3} mbar) in a water bath for 30 min. Toluene (10 mL) is added to the solids and stirred for 65 min at 350 rpm. The solution is filtered (pore 4) resulting in a clear red solution. The filtrate is concentrated *in vacuo* and left to stand at RT for a month, resulting in the formation of crystals of **PacBPhOBu**. The supernatant is removed using a syringe and put in a flask for a second fraction. The dark brown crystals of fraction one is cooled to −80 °C in an isopropanol/nitrogen bath, washed with cold toluene (near freezing point, 3 × 4 mL) and dried *in vacuo* (50 °C, 1×10^{-3} mbar) for 1 h in a water bath. The ^1H NMR spectrum of dried fraction one shows lots of signals and further attempts to isolate **PacBPhOBu** are made. Dichloromethane (3 mL) is added to fraction one, subsequently concentrated *in vacuo* and left to stand overnight in a warm water bath (30 °C) to slowly cool to RT. No crystallization can be observed. The solvent is removed *in vacuo* (1×10^{-3} mbar)

and the residue dried *in vacuo* (50 °C, 1 × 10^{-3} mbar) in a water bath for 10 min. PhF (5 mL) is added to the solids but no dissolution occurs. The solvent is removed *in vacuo* (1 × 10^{-3} mbar) and the residue dried *in vacuo* (50 °C, 1 × 10^{-3} mbar) in a water bath for 10 min. DCB (15 mL) is added to the solids, resulting in a dark red suspension of fine powder and solids left on the inner glass wall of the flask. The flask is put in an ultrasonic bath for 2 min until the inner glass wall is solid free, resulting in a dark red suspension. The suspension is heated to 50 °C in a warm water bath and filtered (pore 4 frit), resulting in a clear red solution. The solution is heated to 50 °C in a water bath and left to slowly cool over the weekend. No crystallization can be observed. The solution is concentrated *in vacuo*. Powder-like precipitation is occurring. The solution is heated to 80 °C in a silicon oil bath until all precipitants are gone and the solution is clear. The solution is left to slowly cool over overnight, with powder-like solids visible the next morning. Dichloromethane (0.6 mL) is added, resulting in a clear solution. The flask is cooled to 5 °C overnight in a fridge with no visible crystallization. Isolation of crystals and full characterization proved unsuccessful.

Raman (784 nm, 10 s, 20 scans, cm^{-1}): $\tilde{\nu}$ = 1601 (10), 1570 (5), 1555 (4), 1531 (5), 1479 (3), 1450 (3), 1424 (2), 1411 (1), 1381 (2), 1371 (2), 1333 (6), 1288 (3), 1272 (7), 1254 (4), 1231 (1), 1184 (3), 1142 (1), 1095 (2), 1070 (1), 1048 (3), 1029 (1), 1000 (2), 959 (2), 940 (2), 869 (2), 837 (2), 785 (1), 765 (2), 738 (3), 640 (3), 584 (1), 510 (2), 464 (2), 447 (2), 411 (2), 383 (2), 367 (2), 321 (2), 267 (3), 223 (3), 195 (3), 162 cm^{-1} (3).

Single crystals of **PacBPhOBu** suitable for X-ray crystallography were received through crystal picking from toluene as described above.

Figure S3. NMR and Raman spectra of **PacBPhOBu** (solvent signals indicated by asterisks).

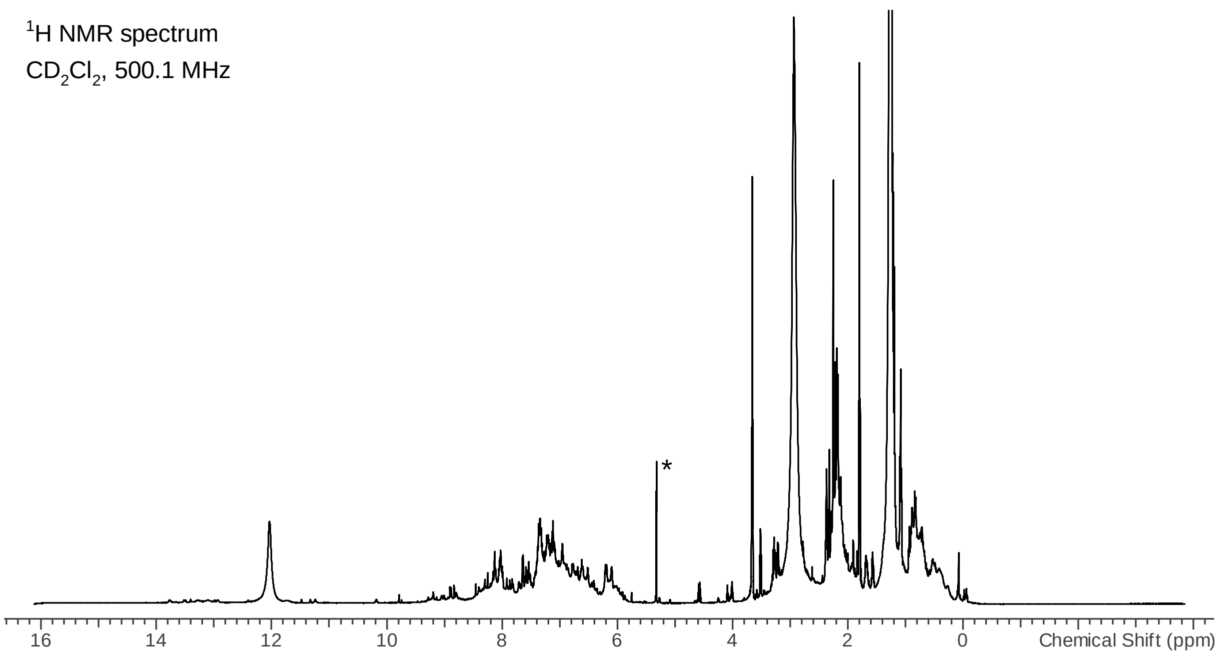

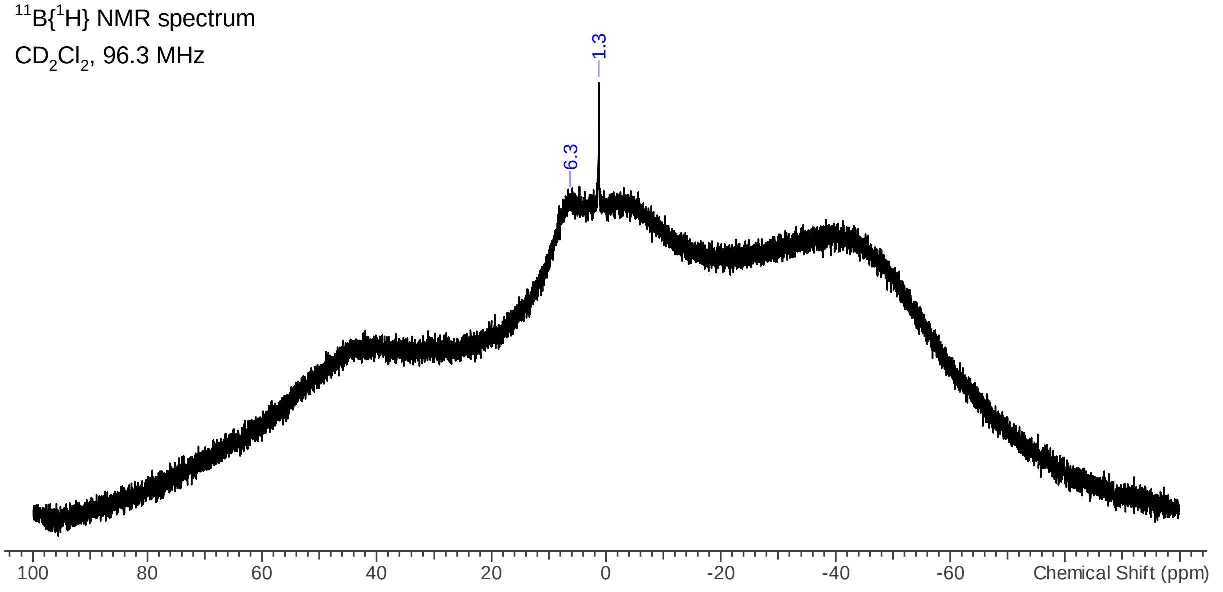

Figure S3 continued.

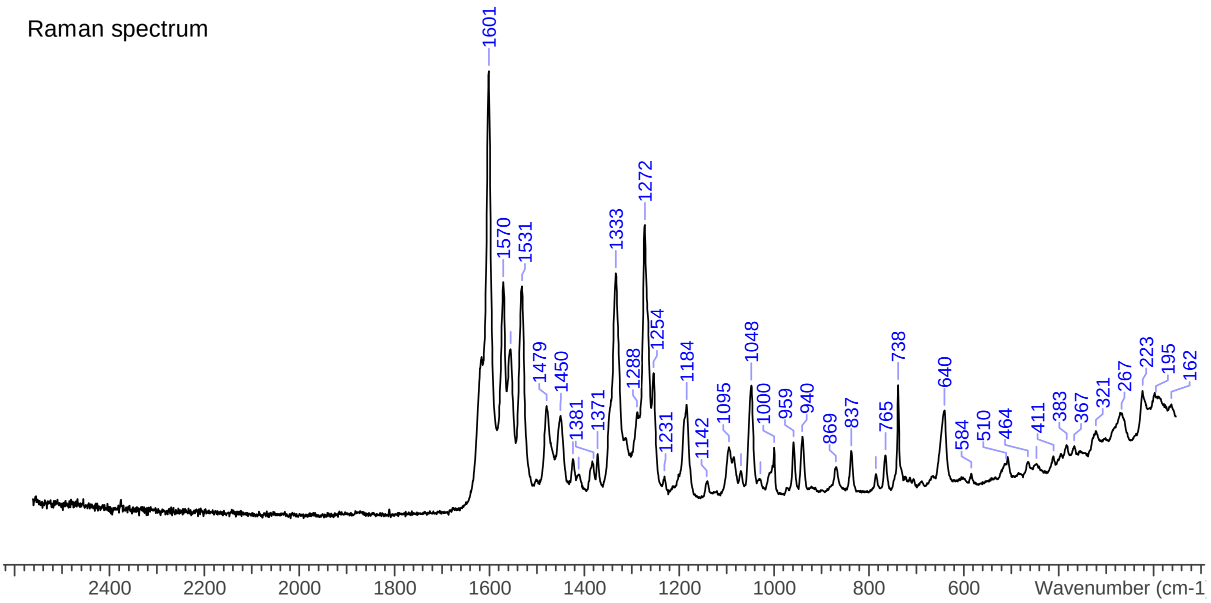

4 Synthesis of non-isolated products

4.1 Reaction of 1a with BPh_3

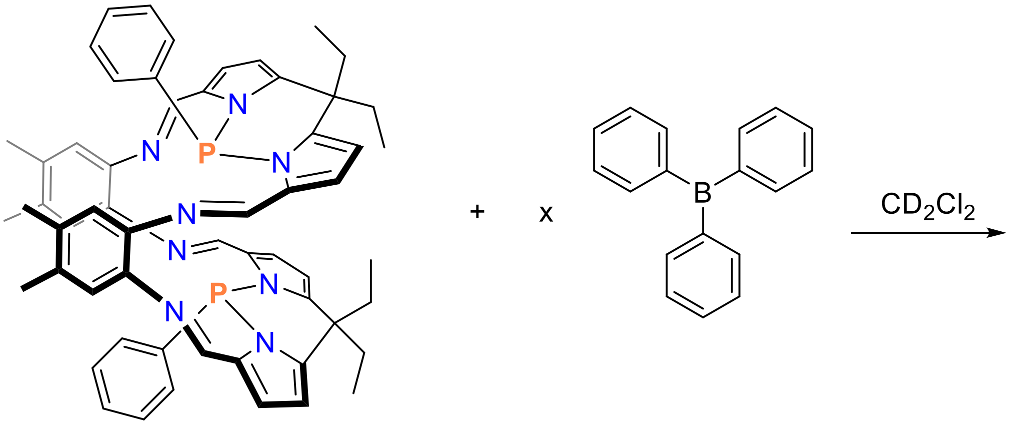

A Yang nmr tube is filled with orange **1a** (25.5 mg, 0.025 mmol) and colourless BPh_3 (6 mg, 0.025 mmol) and dissolved in deuterated dichloromethane (0.5 mL), resulting in a yellow solution. After 24 h another equivalent of BPh_3 (6 mg, 0.025 mmol) is added and another 24 h later four additional equivalents of BPh_3 (24 mg, 0.1 mmol) are added to the solution.

Figure S4. NMR spectra of the reaction of **1a** with BPh_3 (solvent signals indicated by asterisks).

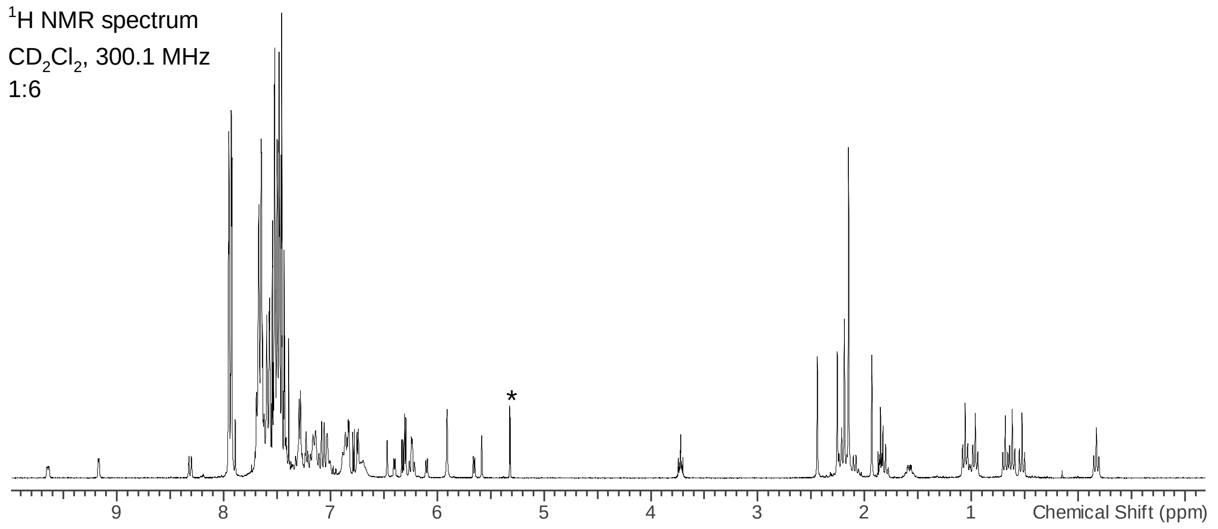

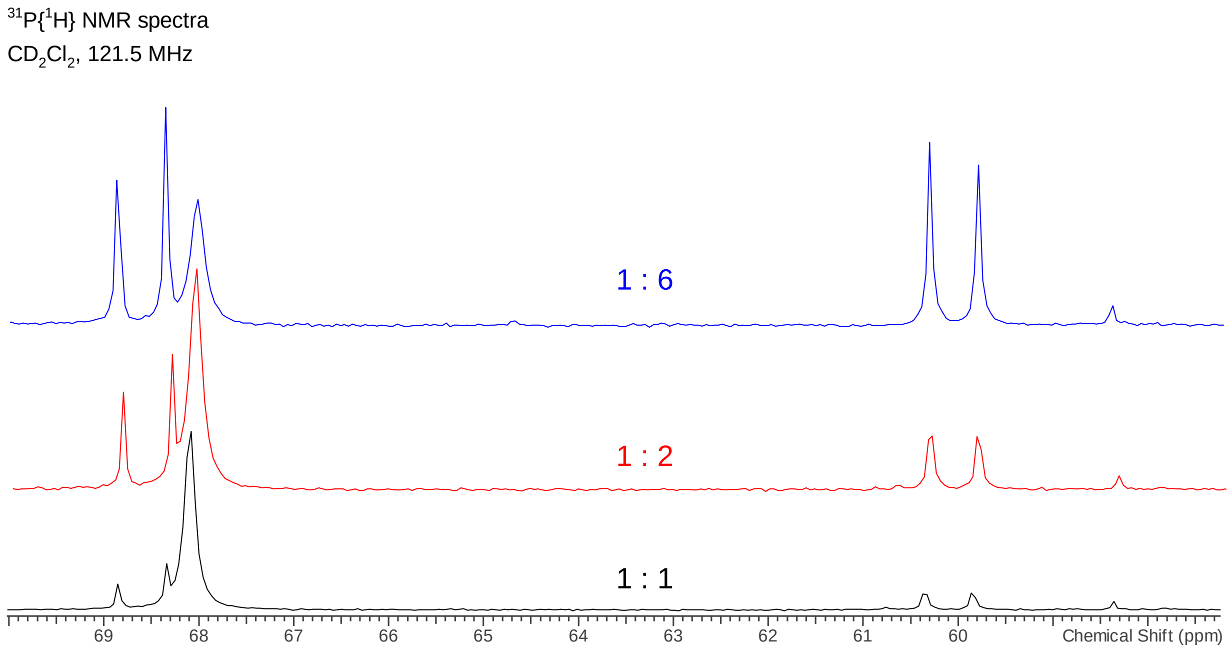

Figure S4 continued.

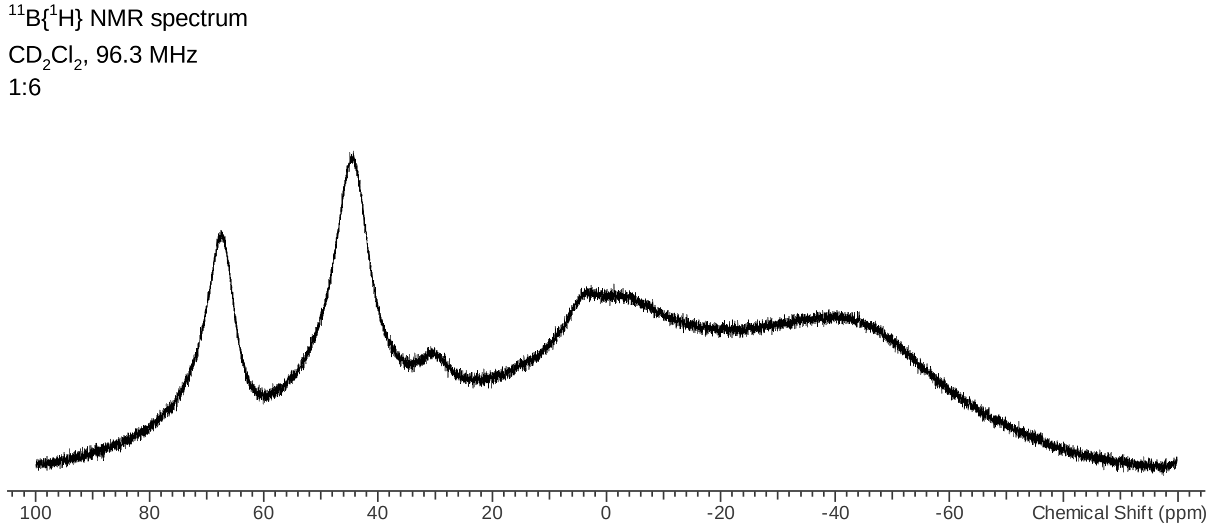

5 Activation attempts

5.1 Activation attempt of CS_2 with a mixture of 1a and BPh_3

A Yang nmr tube is filled with orange **1a** (5 mg, 0.005 mmol) and colourless BPh_3 (6 mg, 0.025 mmol) and dissolved in deuterated dichloromethane (0.5 mL), resulting in a yellow solution. An excess of colourless liquid CS_2 is added at ambient temperature, leading to no reaction.

5.2 Activation attempt of CS_2 with 1a·2B(C_6F_5)$_3$

A Yang nmr tube is filled with yellow **1a**·2B(C_6F_5)$_3$ (19.5 mg, 0.01 mmol) and dissolved in deuterated dichloromethane (0.5 mL), resulting in a yellow solution. An excess of colourless liquid CS_2 is added at ambient temperature, leading to no reaction.

5.3 Activation attempt of EtBr with a mixture of 1a and BPh_3

A Yang nmr tube is filled with orange **1a** (20 mg, 0.02 mmol) and colourless BPh_3 (10 mg, 0.04 mmol) and dissolved in deuterated dichloromethane (0.5 mL), resulting in a yellow solution. An excess of colourless liquid EtBr is added at ambient temperature, leading to no reaction.

5.4 Activation attempt of EtBr with 1a·2B(C_6F_5)$_3$

A Yang nmr tube is filled with yellow **1a**·2B(C_6F_5)$_3$ (23 mg, 0.01 mmol) and dissolved in deuterated dichloromethane (0.5 mL), resulting in a yellow solution. An excess of colourless liquid EtBr is added at ambient temperature, leading to no reaction.

5.5 Activation attempt of C_2H_2 with a mixture of 1a and BPh_3

A Yang nmr tube is filled with orange **1a** (20 mg, 0.02 mmol) and colourless BPh_3 (10 mg, 0.04 mmol) and dissolved in deuterated dichloromethane (0.5 mL), resulting in a yellow solution. The tube is degassed using the *freeze-pump-thaw* method and is filled with one atmosphere of C_2H_2, leading to no reaction.

6 Computational details

6.1 General remarks

Computations were carried out using Gaussian09[7] or ORCA 5.0.1[8] and the standalone version of NBO 6.0.[9–13]

Structure optimizations employed the DFT functional PBE[14,15] in conjunction with Grimme's dispersion correction D3(BJ)[16,17] and the def2-TZVP basis set[18] (notation PBE-D3/def2TZVP). The resolution of identity (RI) approximation was employed, using the appropriate Coulomb fitting basis of the Weigend group.[19] All structures were fully optimized and confirmed as minima by frequency analyses.

Please note that all computations were carried out for single, isolated molecules in the gas phase (ideal gas approximation). There may well be significant differences between gas phase and condensed phase.

6.2 Isomer search

An isomer search was conducted using xTB version 6.5.1.[20] Calculations were carried out with the semiempirical tight-binding based quantum chemistry method GFN2-xTB.[20,21] The starting structure was optimized first at the GFN2-xTB level of theory. For the isomer search the low-energy chemical space was explored using the computer code CREST[22,23]. An evaluation of the found ensemble of isomers was performed with the CENSO algorithm[24]. This included a cheap pre-screening and a pre-screening (B97-D3/def2-SV(P) level of theory)[25] with the final level b97-3c + SMD[ch2cl2] + GmRRHO(GFN2[alpb]-bhess) // b97-3c[SMD].

Note in the following tables only the energetically preferred species are listed.

6.3 Summary of calculated data

6.3.1 BR_3 adducts

Table S3. Results of the isomer search for a given starting material, and the corresponding lowest Gibbs free energy (***G*****best**) for the most thermodynamically favoured isomer. #crest = number of unique conformers < 6 kcal/mol, #censo = number of unique conformers < 3 kcal/mol.

**starting materials**		**#crest**	**#censo**	G_{best} **in a.u.**
BH_3		1	1	-26.581118
BPh_3		2	1	-719.258401
$B(C_6F_5)_3$		2	1	-2207.415372
exo-exo-Pacman-P	**1a**	178	15	-3363.616733
endo-exo-Pacman-P	**1b**	159	2	-3363.612911
**Monoadducts**		**#crest**	**#censo**	G_{best} **in a.u.**
exo-exo-N-BH_3	**1a·N-BH_3**	109	25	-3390.238581
exo-exo-N-BPh_3	**1a·N-BPh_3**	30	3	-4082.880384
exo-exo-N-$B(C_6F_5)_3$	**1a·N-$B(C_6F_5)_3$**	27	4	-5571.043463
**Monoadducts**		**#crest**	**#censo**	G_{best} **in a.u.**
exo-exo-P-BH_3	**1b·N-BH_3**	118	1	-3390.201234
exo-exo-P-BPh_3	**1b·N-BPh_3**	282	25	-4082.854235
exo-exo-P-$B(C_6F_5)_3$	**1b·N-$B(C_6F_5)_3$**	133	run	-5570.927745
**Diadducts**		**#crest**	**#censo**	G_{best} **in a.u.**
exo-exo-NN-BH_3	**1a·NN-2BH_3**	196	40	-3416.863190
exo-exo-NN-BPh_3	**1a·NN-2BPh_3**	56	5	-4802.142557
exo-exo-NN-$B(C_6F_5)_3$	**1a·NN-2$B(C_6F_5)_3$**	27	3	-7778.46492
**Monoadducts**		**#crest**	**#censo**	G_{best} **in a.u.**
endo-exo-N-BH_3	**1b·N-BH_3**	75	13	-3390.235984
endo-exo-N-BPh_3	**1b·N-BPh_3**	20	10	-4082.869008
endo-exo-N-$B(C_6F_5)_3$	**1b·N-$B(C_6F_5)_3$**	21	9	-5571.032695
endo-exo-P-Bh_3	**1b·P-BH_3**	150	10	-3390.202317
endo-exo-P-BPh_3	**1b·P-BPh_3**	356	9	-4082.861659
endo-exo-P-$B(C_6F_5)_3$	**1b·P-$B(C_6F_5)_3$**	179	2	-5571.019090

Table S4. Difference of Gibbs free energy ΔG_{rel} in relation to the lowest Gibbs free energy calculated ΔG_{best} for the monoadducts of the given Lewis acid and either the nitrogen or the phosphorous atom of the exo-exo or endo-exo Pacman isomers.

	**rel. Energies**	BH_3	
	**Monoadducts**	**G_{best} in a.u.**	**ΔG_{rel} in kcal/mol**
exo-exo	N	-3390.238581	0.00
exo-exo	P	-3390.201234	23.44
endo-exo	N	-3390.235984	1.63
endo-exo	P	-3390.202317	22.76
	**rel. Energies**	BPh_3	
	**Monoadducts**	**G_{best} in a.u.**	**ΔG_{rel} in kcal/mol**
exo-exo	N	-4082.880384	0.00
exo-exo	P	-4082.854235	16.41
endo-exo	N	-4082.869008	7.14
endo-exo	P	-4082.861659	11.75
	**rel. Energies**	$B(C_6F_5)_3$	
	**Monoadducts**	**G_{best} in a.u.**	**ΔG_{rel} in kcal/mol**
exo-exo	N	-5571.043463	0.00
exo-exo	P	-5571.010823	20.48
endo-exo	N	-5571.032695	6.76
endo-exo	P	-5571.019090	15.29

Table S5. Calculated Gibbs free energy ΔG at 298 K for the listed reactions.

**borane + pacman = monoadduct**	**ΔG(298) in a.u.**	**ΔG(298) in kcal/mol**
N-BH_3	-0.040730	-25.56
N-BPh_3	-0.005250	-3.29
N-$B(C_6F_5)_3$	-0.011358	-7.13
**monoadduct + borane = diadduct**	**ΔG(298) in a.u.**	**ΔG(298) in kacl/mol**
NN-BH_3	-0.043492	-27.29
NN-BPh_3	-0.003772	-2.37
NN-$B(C_6F_5)_3$	-0.006085	-3.82
**2borane + pacman = diadduct**	**ΔG(298) in a.u.**	**ΔG(298) in kcal/mol**
NN-BH_3	-0.084222	-52.85
NN-BPh_3	-0.009022	-5.66
NN-$B(C_6F_5)_3$	-0.017443	-10.95
**monoadduct = 1/2diadduct + 1/2pac**	**ΔG (298) in a.u.**	**ΔG(298) in kcal/mol**
NN-BH_3	-0.001381	-0.87
NN-BPh_3	0.000739	0.46
NN-$B(C_6F_5)_3$	0.002636	1.65

6.3.2 $GaCl_3$ adducts

Table S6. Results of the isomer search for a given starting material, and the corresponding lowest Gibbs free energy (***G*****best**) for the most thermodynamically favoured isomer. #crest = number of unique conformers < 6 kcal/mol, #censo = number of unique conformers < 3 kcal/mol.

starting materials	#crest	#censo	G_{best} in a.u.
$GaCl_3$	1	1	-3306.146242
**1a**	178	15	-3363.616733
**1a**·$GaCl_3$, mono-N-adduct	227	14	-6669.805638
**1a**·2$GaCl_3$, di-NN-adduct	98	7	-9975.997535
**2**$GaCl_4$	101	10	-9975.999271

Table S7. Calculated Gibbs free energies **Δ*G*** for reactions of **1a** and $GaCl_3$.

reaction	Δ*G* in a.u.	Δ*G* in kcal/mol
**1a** + $GaCl_3$ = **1a**·$GaCl_3$	-0.042663	-26.77
**1a**·$GaCl_3$ + $GaCl_3$ = **1a**·2$GaCl_3$	-0.045655	-28.65
**1a** + 2$GaCl_3$=**1a**·2$GaCl_3$	-0.088318	-55.42
**1a**·2$GaCl_3$ + 2$GaCl_3$ = Ga-salt	-0.090054	-56.51

Table S8. Absolute and relative Gibbs free energies of all considered gallium species.

	G_{best} in a.u.	ΔG_{rel} in a.u.	ΔG_{rel} in kcal/mol	
Pac+2$GaCl_3$	-9975.909220	0.00	0.00	
Mono + $GaCl_3$	-9975.95188	-0.042662759	-26.77	
diadduct	-9975.997535	-0.088317503	-55.42	0.00
Ga-salt	-9975.999271	-0.090053676	-56.51	**-1.09**

6.4 NBO analysis

Level of theory: PBE0-D3/def2-SVP[26] // single point @ xray structure

locX in % = localization of an NBO at atom X

sp^{λ}(X) = hybrid at atom X

6.4.1 Selected NBO data of 1a·2B(C_6F_5)$_3$

Table S9. Natural charge, electron localization and natural bonding orbital hybridization for selected atoms and bonds of **1a·2B(C_6F_5)$_3$**.

atom	charge in e		h(lone pair)
B1	-0.36709	P1	sp0.61
B2	-0.36708	P2	sp0.01
**bonds**	**locB in %**	**h(B)**	**h(N)**
B1-N	20	sp3.95	sp1.75
B2-N	20	sp3.95	sp1.75
	**loc P in %**	**h(P)**	**h(B)**
N1-P1	21	sp15.8	sp2.55
N2-P1	24	sp6.15	sp2.47
N3-P2	21	sp15.8	sp2.55
N4-P2	24	sp6.15	sp2.47

6.4.2 Selected NBO data of 2$^+$

Table S10. Natural charge, electron localization and natural bonding orbital hybridization for selected atoms and bonds of **2$^+$**.

	charge in e	h(lone pair)	
P43	1.19685	sp0.58	
P63	1.21098	sp0.77	
N18		sp2.76	
	**locP in %**	**h(N)**	**h(P)**
N29-P43	15	sp2.4	sp8.2
P43-N58	8	sp3.56	sp26
P43-N59	20	sp2.73	sp6.23
	**locP in %**	**h(P)**	**h(N)**
P63-N73	23	sp8.8	sp2.4
N56-P63	24	sp6	sp2.43

Table S11. Calculated donor acceptor energies between selected orbitals.

donor σ	acceptor σ*	kcal/mol
N29-P43	P43-N58	81
N29-P43	P43-N59	33
P43-N58	N29-P43	48
P43-N58	P43-N59	24
P43-N59	P43-N58	41
P43-N59	N29-P43	34

6.5 Summary of optimized molecular structures in the single crystal

Table S12. Summary of calculated data, including electronic energies and thermal corrections.

Compd.	PG	Opt. method	E_{tot}[a]	ΔG[b]
**1a**·2B(C_6F_5)$_3$	C_1	PBE-D3 def2-TZVP	−7777.5082	1.1451
**2**$GaCl_4$	C_1		−6207.9516	0.9063
**PacBPhOBu**	C_1		−4116.5346	1.1483

[a] Total SCF energy in a.u.; [b] thermal correction to Gibbs energy in a.u. (298 K unless stated otherwise)

7 XYZ Structure of the "best" Isomer

```
BH3
  4
G(CENSO)=         -26.58111761  G(xTB)=         -2.73382428        !CONF1
B    0.0000002355   0.0001312941  -0.0000306353
H    0.0001857967   1.1915916146   0.0000102119
H    1.0315911467  -0.5959919853   0.0000102196
H   -1.0317771540  -0.5956690909   0.0000102149
```

```
BPh3
  34
G(CENSO)=        -719.25840111  G(xTB)=        -47.15962551        !CONF1
B    0.0006494062  -0.0009613262   0.0008456685
C   -0.2274014738   1.5414137513   0.0002354466
C    0.6405324545   2.4089308199   0.6818847507
C    0.4298234747   3.7794959791   0.6980621077
C   -0.6402103160   4.3254993434  -0.0008313047
```

```
C   -1.5056056692   3.4920801630  -0.6993645186
C   -1.3095756567   2.1193499491  -0.6821347293
C   -1.2217939904  -0.9688064820   0.0002082955
C   -2.4100315645  -0.6441870689   0.6737621114
C   -3.4932306033  -1.5099245688   0.6904144103
C   -3.4289027650  -2.7150265724   0.0011192264
C   -2.2714180258  -3.0546917510  -0.6891515406
C   -1.1795946575  -2.1999171511  -0.6731959368
C    1.4506395542  -0.5739606274   0.0018208583
C    1.7657793561  -1.7663341034   0.6725654741
C    3.0578325789  -2.2697002078   0.6869608925
C    4.0677792942  -1.6090204254  -0.0021869174
C    3.7812820628  -0.4352200352  -0.6890144572
C    2.4944526407   0.0811912599  -0.6705512647
H    2.2878283358   1.0011297415  -1.2019224379
H    4.5640402892   0.0794362759  -1.2309587092
H    5.0743930458  -2.0070221283  -0.0035172423
H    3.2782961275  -3.1809415074   1.2276641422
H    0.9870550197  -2.2963876981   1.2053167583
H   -2.4778217998   0.2951305974   1.2069984019
H   -0.2814033173  -2.4815712932  -1.2071645504
H   -2.2193172392  -3.9900164576  -1.2311357296
H   -4.2775082350  -3.3870107007   0.0018456795
H   -4.3911500961  -1.2439727689   1.2329654499
H    1.4837428333   1.9990314017   1.2225249575
H    1.1030159810   4.4245843818   1.2476271719
H   -1.9976199220   1.4815211839  -1.2216757717
H   -2.3370311871   3.9137722158  -1.2491092124
H   -0.7988735111   5.3962516305  -0.0011611696
```

```
B(C6F5)3
  34
G(CENSO)=        -2207.41537183  G(xTB)=         -110.60549684         !CONF1
B    0.0010741401  -0.0087214209  -0.0025431496
C    1.5515135807   0.1594504895   0.0033346938
C    2.3838711829  -0.6328494385  -0.7860216930
C    3.7581158585  -0.4808714843  -0.8109784475
C    4.3481247015   0.4816006249  -0.0052451875
C    3.5596835650   1.2855453774   0.8051250215
C    2.1873011808   1.1185654386   0.7907068333
C   -0.6310426609  -1.4338039169  -0.0057276922
C   -1.7436652184  -1.7480759447  -0.7852390483
C   -2.3186771573  -3.0054391708  -0.7970160894
C   -1.7883947212  -4.0003374341   0.0113417300
C   -0.6849908610  -3.7313850023   0.8075889963
C   -0.1233516356  -2.4680576276   0.7798609544
C   -0.9158032117   1.2529783956  -0.0029129488
C   -2.0683845681   1.3310624275   0.7777656283
C   -2.8835840006   2.4480395653   0.7990790242
C   -2.5644677656   3.5355873628  -0.0006396637
C   -1.4322102031   3.4981729248  -0.8010799569
C   -0.6291310411   2.3727227795  -0.7825914850
F   -2.3370972373  -5.2145050064   0.0216708826
F   -3.3743306449  -3.2749028415  -1.5734600454
F   -2.2838149430  -0.8221908360  -1.5971395185
F    0.9318774090  -2.2516269284   1.5848392504
F   -0.1836034920  -4.6921086964   1.5921874540
F    1.4672301078   1.9097009832   1.6059685321
```

```
F    1.8587685442  -1.5667849073  -1.5986920442
F    4.5223590587  -1.2454181436  -1.5992199953
F    5.6719114031   0.6340333562  -0.0096483909
F    4.1342638803   2.2065638672   1.5873441343
F   -2.4120362295   0.3090290164   1.5809604860
F   -3.9700348224   2.4930807227   1.5785268605
F   -3.3452886172   4.6152721763  -0.0004940363
F   -1.1322530499   4.5462831538  -1.5763014913
F    0.4481965621   2.3794628859  -1.5874045729
```

```
GaCl3
  4
G(CENSO)=       -3306.14624227  G(xTB)=         -15.07511019        !CONF1
Ga  -0.0007185729   0.0006787784   0.0013723981
Cl  -0.8883668909  -1.9421231534  -0.0004314808
Cl   2.1288172301   0.1968540317  -0.0004315996
Cl  -1.2398684155   1.7445556822  -0.0004315462
```

```
1a
  126
G(CENSO)=       -3363.61673311  G(xTB)=        -181.54588644        !CONF123
C   -1.5151899017  -0.9871708993  -2.9508115065
C   -0.1197074871  -0.8401839436  -2.7094754318
C   -2.2488858168  -1.0154104223  -4.1300362611
N   -2.4122659012  -1.1997224727  -1.8976174803
H    0.1648736353  -0.6089369832  -1.6815738234
N    0.7832946897  -0.9878693487  -3.6169116727
C   -3.5890170337  -1.2279657098  -3.7911813917
H   -1.8398569999  -0.8733458372  -5.1164481661
C   -3.6689758865  -1.3524752472  -2.4070011173
P   -1.8028811208  -1.3200562881  -0.1902293639
C    2.1265708136  -0.8545560662  -3.2504427463
H   -4.4229516506  -1.2741483917  -4.4710489323
C   -4.9017140311  -1.4923795239  -1.5703484729
N   -3.3730166096  -1.8619077807   0.4428366759
C    3.0101424982  -1.8293718217  -3.7232590624
C    2.6484936003   0.1712411018  -2.4326918480
C   -4.6082207537  -1.9043074680  -0.1599527487
C   -5.8586812299  -2.5186823553  -2.2219788185
C   -5.6353756958  -0.1190297076  -1.5385004823
C   -3.5099550667  -2.2791393741   1.7723590901
H    2.6007219325  -2.6007181611  -4.3634368137
C    4.3524171904  -1.8684920122  -3.3772586122
C    4.0013641950   0.1297402766  -2.0907829489
N    1.8907593040   1.2324534521  -1.9296753349
C   -5.5267111041  -2.3424910212   0.7865037340
H   -6.7931901570  -2.5100495959  -1.6623341797
H   -6.1046082256  -2.1514874450  -3.2173786090
C   -5.3248639499  -3.9338554391  -2.3119784224
H   -5.9056524487   0.1283758048  -2.5655440238
H   -6.5666590649  -0.2586676496  -0.9884976681
C   -4.8364444507   1.0108310614  -0.9247027359
C   -2.3884297131  -2.3631959921   2.6282161302
C   -4.8454956745  -2.5844022589   1.9834682068
C    4.8614981587  -0.8626932973  -2.5364771528
```

```
C    5.2336501210  -2.9635069046  -3.8897521971
H    4.3830025473   0.9216601653  -1.4622532284
C    1.3359460194   2.0926655594  -2.7064801384
H   -6.5789758252  -2.4808436990   0.6075988557
H   -6.0491552904  -4.5823432145  -2.8045152896
H   -5.1284185670  -4.3512783386  -1.3255147102
H   -4.3994239883  -3.9787098076  -2.8840746814
H   -3.9090021115   1.1906484230  -1.4659078271
H   -4.5810393712   0.8054034088   0.1143205264
H   -5.4116639595   1.9354328117  -0.9438406632
H   -2.5290553030  -2.8074282381   3.6143105535
N   -1.2513941868  -1.9246495047   2.2156330835
H   -5.2601300980  -2.9434784163   2.9109489322
C    6.2999783432  -0.8489604982  -2.1254285850
H    4.6877746744  -3.6307947530  -4.5524842218
H    5.6421387957  -3.5646681184  -3.0752780223
H    6.0885840663  -2.5688837399  -4.4403760310
H    1.4140969419   2.0411786858  -3.7937958872
C    0.6153831209   3.1847089121  -2.1717251439
C   -0.0520844562  -2.1744860309   2.8656055532
H    6.5130219409  -0.0055380026  -1.4729189499
H    6.9680220830  -0.7822099382  -2.9852321396
H    6.5732633427  -1.7608915252  -1.5922055356
C    0.0640809851   4.2777286802  -2.8222237589
N    0.4381468633   3.3698243058  -0.7931097922
C    0.8728582974  -1.1305634478   3.0616363336
C    0.2980040714  -3.4670569659   3.2595525109
H    0.0734731759   4.4316685390  -3.8888385477
C   -0.4565449608   5.1313406453  -1.8453474590
C   -0.2247325101   4.5594648164  -0.6015208713
P    0.7406985866   2.0262540560   0.3484315490
C    2.1043338227  -1.4347629506   3.6449842170
N    0.5540731307   0.1541992878   2.6379628077
C    1.5339464280  -3.7675487441   3.8166720827
H   -0.4085654002  -4.2650730267   3.0709694987
H   -0.9500012633   6.0738782166  -2.0095771647
C   -0.6757430569   5.0932298067   0.7208981991
C    2.5332457124   2.2567793059   0.7236171657
N    0.1658458729   2.9667475608   1.7965122183
H    2.8178711621  -0.6318369814   3.7762261816
C    2.4583957857  -2.7274426654   4.0132715697
C    0.6636468206   1.1456747848   3.4493062799
C    1.8761910223  -5.1817627430   4.1642093685
C   -0.2640994289   6.5778119607   0.8512822069
C   -2.2351098038   5.0399753699   0.7655082150
C   -0.1588691262   4.2995994858   1.8764917778
C    3.1538998905   3.5005760257   0.6736166490
C    3.2666098401   1.1373718554   1.1088080813
C    0.4151791610   2.4962015059   3.0899770628
C    3.8136628712  -3.0013436335   4.5834788865
H    0.9091463521   0.9946753370   4.5044160157
H    2.1203744848  -5.2928342993   5.2215603243
H    2.7471704404  -5.5315280637   3.6077255931
H    1.0493707202  -5.8517063165   3.9410384753
H   -0.7607275494   7.1270123458   0.0525917081
H   -0.6839762732   6.9611506523   1.7806392803
C    1.2252271319   6.8502034969   0.8063112607
H   -2.5491688371   5.4878455322   1.7087665571
H   -2.6018383163   5.6880322849  -0.0313279704
C   -2.8345952082   3.6579382862   0.6149136174
```

```
C   -0.1195720672   4.6808263930   3.2103233743
H    2.5919198812   4.3725528833   0.3723067867
C    4.4959460059   3.6239733593   1.0033678741
C    4.6046029760   1.2650992568   1.4530979812
H    2.7964708304   0.1653086899   1.1363477958
C    0.2488302905   3.5618259029   3.9643837757
H    3.7528414178  -3.4465883541   5.5774567100
H    4.4005153735  -2.0892670210   4.6621089891
H    4.3728074102  -3.7051806582   3.9646823296
H    1.7519113986   6.3475741102   1.6153814238
H    1.4169741549   7.9187871165   0.9015763775
H    1.6636590850   6.5216600952  -0.1349780196
H   -3.9216942876   3.7194205852   0.6050312299
H   -2.5509656551   2.9958820323   1.4309888155
H   -2.5233049701   3.1890702826  -0.3172517853
H   -0.3649590127   5.6580034792   3.5893331037
H    4.9736376709   4.5937782719   0.9536829000
C    5.2241220987   2.5068866985   1.3967083432
H    5.1659806842   0.3900304690   1.7526309376
H    0.3453883033   3.5007123415   5.0362833487
H    6.2698365814   2.6051979198   1.6568137787
C   -0.9528713358  -2.9454295402  -0.4015617786
C    0.4327591968  -2.9822237361  -0.2655619671
C   -1.6421576167  -4.1043803582  -0.7435638533
C    1.1230870974  -4.1666265175  -0.4749375840
H    0.9734850548  -2.0864697177   0.0081330743
C   -0.9510100564  -5.2908879653  -0.9417553416
H   -2.7170835359  -4.0821523714  -0.8539986959
C    0.4324247422  -5.3235502983  -0.8120639739
H    2.1994443537  -4.1845158921  -0.3707541258
H   -1.4932467926  -6.1903308536  -1.2020908498
H    0.9694503864  -6.2485463082  -0.9737225042
```

```
1b
126
G(CENSO)=       -3363.61291092  G(xTB)=        -181.54477540        !CONF127
C   -1.1835364624  -3.1386945468  -0.3616572364
C   -0.1655694977  -2.8576591753  -1.3077257426
C   -1.5882215228  -4.4042555612   0.0440039556
N   -2.0116387728  -2.2064062291   0.2767319908
H    0.1780741182  -3.7340017359  -1.8643859840
N    0.3303796384  -1.6930811952  -1.5360193773
C   -2.6412890664  -4.2493806295   0.9453630903
H   -1.1412767396  -5.3263891017  -0.2902500007
C   -2.8993277377  -2.8898750490   1.0750431244
P   -1.8120415444  -0.4217865058   0.0476350073
C    1.0751452710  -1.4851425688  -2.6929169515
H   -3.1825741827  -5.0357737511   1.4403907447
C   -3.9442374774  -2.2407705428   1.9306943999
N   -3.4164083789  -0.0403472834   0.7848286269
C    0.5095150078  -1.7996282177  -3.9323930734
C    2.3447588057  -0.8670985055  -2.6705108275
C   -4.2910142519  -0.8666622181   1.4480133580
C   -3.4046773377  -2.0532793613   3.3871573264
C   -5.2132208149  -3.1185168339   1.9615329377
C   -4.0264575388   1.2103012612   0.6297290827
H   -0.4722307919  -2.2530764653  -3.9367696546
C    1.1134352883  -1.4834177581  -5.1396055365
```

C	2.9388801939	-0.5349446074	-3.8963535257
N	2.9322647312	-0.6207402816	-1.4388147439
C	-5.4518701211	-0.1509663147	1.7107529627
H	-2.5089240478	-1.4366321454	3.3203522161
H	-4.1470403794	-1.4619351204	3.9236572373
C	-3.1120144434	-3.3166271838	4.1709328165
H	-4.9473446230	-4.0874513210	2.3750124766
H	-5.9067340075	-2.6768664492	2.6766105451
C	-5.9023750149	-3.3182199498	0.6252491304
C	-3.3857013886	2.3281878189	0.0494826878
C	-5.2921363398	1.1369857166	1.1939596246
C	2.3561216522	-0.8233043293	-5.1208129614
C	0.4185673938	-1.7914566115	-6.4275326640
H	3.8875312817	-0.0152563865	-3.8910205859
C	4.1957635449	-0.3906675194	-1.2901180417
H	-6.3118585465	-0.5307467272	2.2342168027
H	-2.6550136506	-3.0590787307	5.1258433413
H	-2.4251614806	-3.9769975013	3.6477000649
H	-4.0155391385	-3.8798280474	4.3913909183
H	-5.2504742882	-3.8147175235	-0.0916027373
H	-6.2215558740	-2.3748759556	0.1863132511
H	-6.7882383275	-3.9410202777	0.7480401063
H	-3.9983431684	3.2212983791	-0.0887324721
N	-2.1379646502	2.2926519279	-0.2607687777
H	-5.9970679692	1.9512701850	1.2338075071
C	3.0297121472	-0.4198731657	-6.3951965370
H	0.1997674046	-0.8818130009	-6.9897338639
H	-0.5225515851	-2.3070086115	-6.2518489927
H	1.0294433682	-2.4178946610	-7.0792133910
H	4.9270419056	-0.4892740545	-2.0935325580
C	4.6962424076	-0.0266602068	-0.0157014980
C	-1.5227733427	3.3083205877	-0.9731213967
H	3.9739370817	0.0832479259	-6.2002515362
H	2.4066059272	0.2556820681	-6.9835628565
H	3.2357858862	-1.2822455660	-7.0312308778
C	5.9939392562	0.0173795840	0.4776332849
N	3.8384588788	0.2331957111	1.0483091150
C	-0.1768490721	3.6401238771	-0.6934351002
C	-2.1449214246	3.9174628088	-2.0696210785
H	6.8856215162	-0.1578513641	-0.1012804923
C	5.9147949958	0.2823772568	1.8516708217
C	4.5749408663	0.4031875688	2.2001547209
P	2.0865205970	0.4121530883	0.8557401249
C	0.4681745085	4.5530284499	-1.5337197761
N	0.5660366554	3.0633157378	0.3221234226
C	-1.5040367906	4.8440538823	-2.8778014327
H	-3.1540755526	3.6130830280	-2.3156414649
H	6.7442416089	0.3681529547	2.5315332166
C	3.9744797098	0.6032423651	3.5681454650
C	1.6386530303	-1.1955171222	1.6510457147
N	1.9772460440	1.4696696948	2.2988599470
H	1.5041316295	4.7817333343	-1.3181881790
C	-0.1632072009	5.1738714748	-2.5995241904
C	0.0752626803	2.8663258087	1.4975322613
C	-2.2176754871	5.4509445153	-4.0443687245
C	3.8040919632	-0.7636524522	4.3033393851
C	4.9060481869	1.5131433496	4.4074496449
C	2.6217106491	1.2590987108	3.4947255772
C	2.3111486690	-2.3654371421	1.3017112976
C	0.6022898986	-1.2580477368	2.5779305141

```
C    0.8197022591   2.2196194888   2.5310969257
C    0.5822648344   6.1525309566  -3.4513813719
H   -0.8940696076   3.2655196728   1.7988986416
H   -3.2384889088   5.0832811767  -4.1178378881
H   -1.7133543990   5.2268762239  -4.9857758328
H   -2.2592140142   6.5387537307  -3.9687753793
H    3.4243633742  -0.5278968030   5.2984967801
H    3.0184429850  -1.3198415363   3.8006819870
C    5.0341948085  -1.6404699547   4.4174414860
H    4.5027485084   1.5459007530   5.4194149037
H    5.8768679690   1.0297191019   4.4890026201
C    5.0822127050   2.9236942262   3.8782044321
C    1.8668002604   1.8414781280   4.4972266702
H    3.1149524095  -2.3338372339   0.5818708182
C    1.9664827106  -3.5743410522   1.8872686885
C    0.2785461408  -2.4643090981   3.1818523119
H    0.0701435202  -0.3604695875   2.8610198949
C    0.7348477870   2.4278377223   3.8998380839
H    1.6013090839   6.2856121143  -3.0963383150
H    0.1004074776   7.1315034457  -3.4571747434
H    0.6308414551   5.8265836204  -4.4915491835
H    4.7856544651  -2.5412718006   4.9789285486
H    5.8556825125  -1.1508481055   4.9378748760
H    5.3959745521  -1.9555016203   3.4407154422
H    5.4817079376   2.9271771060   2.8650335284
H    5.7764688668   3.4807141364   4.5074495827
H    4.1393390014   3.4679172439   3.8654552705
H    2.1361966842   1.8924376345   5.5389424686
H    2.4982333580  -4.4746861436   1.6088135367
C    0.9541891042  -3.6263701399   2.8364582350
H   -0.4983269037  -2.4945653966   3.9296231302
H   -0.0201530106   3.0216199455   4.3896549634
H    0.6926292011  -4.5662435455   3.3044052505
C   -2.3633682157  -0.3369649567  -1.7152511110
C   -3.5035452090  -1.0040431982  -2.1528389537
C   -1.6232466833   0.4229604060  -2.6149224638
C   -3.9002730881  -0.9088534730  -3.4793205282
H   -4.0839503721  -1.5972685878  -1.4594561898
C   -2.0260488773   0.5264914854  -3.9375008442
H   -0.7300378246   0.9293668711  -2.2821743434
C   -3.1638411488  -0.1400546740  -4.3734428456
H   -4.7865150831  -1.4320328299  -3.8136420910
H   -1.4417698882   1.1179140770  -4.6295281029
H   -3.4746607652  -0.0641815008  -5.4071938907
```

```
1a-N-BH3
  130
G(CENSO)=        -3390.23858071  G(xTB)=         -184.31065553         !CONF7
C   -0.4725435091  -1.8541383075  -2.2609071413
C    0.3880550325  -0.7473214264  -2.3422423309
C   -1.4010976099  -2.3975715038  -3.1597872159
N   -0.5104555350  -2.6566512453  -1.0981439036
H    0.9992110301  -0.5406894918  -1.4780920986
N    0.5622805930   0.0766405273  -3.3443655531
C   -1.9962612756  -3.4903481300  -2.5495976794
H   -1.5951405088  -2.0265451743  -4.1470618091
C   -1.4361008602  -3.6335494831  -1.2777867669
P    0.2547036766  -2.1177735454   0.4953033758
```

C	1.5385470024	1.1100668016	-3.1465169506
H	-2.7465156475	-4.1329350039	-2.9766966176
C	-1.8116147535	-4.6587544738	-0.2518121816
C	2.0037841479	-2.3442937212	-0.0549640875
N	-0.0193415587	-3.7029517092	1.2563185240
C	2.8861397898	0.8498851495	-3.3156716125
C	1.1100565018	2.4028915145	-2.8378761823
C	-0.8237693321	-4.7440020881	0.8657408469
C	-1.9595292786	-6.0381442599	-0.9332534919
C	-3.2006060801	-4.2739733914	0.3470141623
C	2.4550005376	-3.5480343692	-0.5886117272
C	2.8678129731	-1.2531963906	0.0139621819
C	0.6513621809	-4.0702756715	2.4266663088
H	3.1915126113	-0.1589943213	-3.5595856247
C	3.8439642803	1.8518686325	-3.1781066679
C	2.0618612384	3.4152478197	-2.7344873121
N	-0.2167930458	2.5922363722	-2.4837683327
C	-0.6508147967	-5.7798441511	1.7757607323
H	-2.3093226285	-6.7435014264	-0.1809357090
H	-2.7629017529	-5.9643937681	-1.6645523534
C	-0.7036181246	-6.5674723323	-1.5958861823
H	-3.9014269119	-4.2087120740	-0.4852930689
H	-3.5256067111	-5.1054311598	0.9730074058
C	-3.2206447822	-2.9868426371	1.1448655173
H	1.7870375424	-4.3961112037	-0.6570590393
C	3.7618767812	-3.6609210639	-1.0403430606
C	4.1719702608	-1.3661907235	-0.4449811746
H	2.5224156522	-0.3140385008	0.4244942865
C	1.4255482146	-3.1141192026	3.1192607678
C	0.2718558424	-5.3652982001	2.7427044374
C	3.4194062713	3.1618175769	-2.8977906433
C	5.2961644033	1.5250504960	-3.3243184561
H	1.7273805027	4.4089470952	-2.4686564093
C	-1.0933114837	3.3187952893	-3.0765308834
H	-1.1532806085	-6.7305678884	1.7373360827
H	-0.3520237860	-5.9030134192	-2.3840966225
H	-0.8953069280	-7.5401248558	-2.0483663353
H	0.1067687576	-6.6924054210	-0.8794899902
H	-2.6411946743	-3.0658984007	2.0628548064
H	-4.2412450887	-2.7269717622	1.4232130776
H	-2.8236041842	-2.1536845366	0.5702329602
H	4.1078335803	-4.5998384742	-1.4520581952
C	4.6218600130	-2.5704259577	-0.9706567880
H	4.8344844104	-0.5124920746	-0.3939447593
H	1.9716115470	-3.4222542852	4.0106124030
N	1.4162816869	-1.9030135955	2.6845280631
H	0.6146834840	-5.9206939065	3.6001078934
C	4.4096520609	4.2726728007	-2.7419945119
H	5.8518698878	1.7559113241	-2.4137596206
H	5.7606879244	2.1023583030	-4.1251401774
H	5.4421157075	0.4701208464	-3.5436041089
H	-0.8980718646	3.8705255646	-3.9942403510
C	-2.3842206274	3.3293743705	-2.5057891097
H	5.6389281030	-2.6595089514	-1.3291147863
C	2.2098457488	-0.8883141571	3.2075473609
H	3.9132396868	5.2158665518	-2.5269880945
H	5.0085008250	4.4065240046	-3.6438249357
H	5.1111303168	4.0709069982	-1.9309155580
C	-3.5943048839	3.8678594751	-2.9123732772
N	-2.6152376520	2.5748195739	-1.3496278799

C	1.6319470712	0.3622670588	3.4893194831
C	3.5867842010	-1.0548650798	3.3801560776
H	-3.7381087167	4.4890230326	-3.7809859003
C	-4.5683671978	3.4338417127	-2.0072614104
C	-3.9530685488	2.6360005928	-1.0481649043
P	-1.2747476288	1.7414430891	-0.5211924932
C	2.4613240582	1.3850019076	3.9452296083
N	0.2475050661	0.5689805307	3.4328273906
C	4.4117683184	-0.0233877857	3.8050479842
H	4.0202129620	-2.0133353131	3.1245794872
H	-5.6199258343	3.6600719005	-2.0417253297
C	-4.6183881903	1.8446297568	0.0343209447
C	-0.4112579857	3.1966486040	0.2241013778
N	-2.3055292996	1.3886098791	0.9588469748
H	2.0065033172	2.3402128776	4.1754334502
C	3.8347903127	1.2275752169	4.0889898110
C	-0.3217698426	0.7349293000	2.2926294651
C	5.8874373358	-0.2397205126	3.9318238999
C	-5.7549071039	2.6783663185	0.6696320094
C	-5.2627681549	0.5818330304	-0.6157864664
C	-3.6621646192	1.4079053627	1.1027908541
C	-1.1118952059	4.3146321137	0.6667085737
C	0.9710896988	3.1436777526	0.3848392683
C	-1.7323233466	0.8977582325	2.1367202486
C	4.6798244265	2.3819130036	4.5271230267
H	0.2751530630	0.7492704635	1.3808484230
H	6.1579720870	-1.2635007540	3.6834493195
H	6.2399294048	-0.0382301117	4.9445937736
H	6.4497284578	0.4228619814	3.2714214066
H	-6.4778684316	2.9125951889	-0.1105844898
H	-6.2791762563	2.0360536469	1.3759617985
C	-5.3119355392	3.9529643226	1.3599160829
H	-5.7115178626	-0.0001729129	0.1896306831
H	-6.0771319203	0.9216619369	-1.2572182460
C	-4.3069418441	-0.2823264866	-1.4127370453
C	-3.9704922649	0.9232342548	2.3716897018
H	-2.1863861348	4.3617219624	0.5527523433
C	-0.4347926949	5.3722643922	1.2562678522
C	1.6462274022	4.1998792958	0.9795578241
H	1.5233445356	2.2787522552	0.0437873003
C	-2.7697211084	0.6183793468	3.0183776548
H	4.0735155344	3.2648992017	4.7148464398
H	5.4239378047	2.6428311059	3.7724895546
H	5.2330366950	2.1544934800	5.4395618825
H	-4.6220304530	3.7495232911	2.1773244736
H	-6.1715550509	4.4769275905	1.7775230009
H	-4.8185319304	4.6332003304	0.6670645221
H	-3.9581823876	0.2216443149	-2.3124941440
H	-4.7925789764	-1.2071303491	-1.7225957693
H	-3.4327586090	-0.5577975366	-0.8272113505
H	-4.9631663962	0.8122241539	2.7744145082
H	-0.9859090383	6.2398738060	1.5944398739
C	0.9454957709	5.3172636208	1.4141640749
H	2.7204847434	4.1503181253	1.0988241997
H	-2.6460161675	0.2165048306	4.0103076728
H	1.4715867343	6.1423238171	1.8760969972
B	-0.1771159256	0.0693414949	-4.7352314189
H	-1.3806122483	0.1623470968	-4.5471921916
H	0.2375134027	1.0103582821	-5.3810959824
H	0.0705347863	-0.9866000842	-5.3006166190

```
1a-N-BPh3
  160
G(CENSO)=       -4082.88038413  G(xTB)=        -228.72660032        !CONF1
C   -4.3048771871  -0.1851571768   0.1878881498
C   -4.0305324803   0.1419892788  -1.1604676803
C   -5.3328619371   0.3045313071   0.9862465009
N   -3.5479995675  -1.0712402403   0.9694444973
H   -4.6915958391   0.8932671969  -1.5960056797
N   -3.0854107108  -0.4112281330  -1.8389217397
C   -5.1983174292  -0.2670921111   2.2519230324
H   -6.0914798691   0.9961866096   0.6575513029
C   -4.0907404947  -1.1079625128   2.2290435988
P   -1.9287575248  -1.6428284611   0.4123992098
C   -2.7647662223   0.0322421949  -3.1160206291
H   -5.8338984235  -0.1022560093   3.1048573673
C   -3.5381479371  -1.8872766328   3.3839908819
C   -2.4617128047  -3.0787670251  -0.6289531219
N   -1.6508300498  -2.6506052716   1.8953227810
C   -2.5948724276  -0.9041240165  -4.1374151697
C   -2.5326556564   1.3928953438  -3.4187881501
C   -2.4710036137  -2.8499907736   2.9757516422
C   -4.6875325734  -2.6283361230   4.1041626800
C   -2.9151816576  -0.8848607389   4.4067045606
C   -1.7709329071  -3.3409655918  -1.8067647278
C   -3.5018418787  -3.9181961434  -0.2437904164
C   -0.6551505626  -3.6346929894   1.9079256021
H   -2.7724336457  -1.9429184615  -3.9008508756
C   -2.2214153178  -0.5509933106  -5.4261174155
C   -2.1569809456   1.7450004794  -4.7172831749
N   -2.4988943438   2.3148902261  -2.3881924567
C   -2.0021959491  -3.9529830254   3.6800969478
H   -4.2668359648  -3.1319866073   4.9733677821
H   -5.3686920528  -1.8761834540   4.5001521948
C   -5.4541253462  -3.6216645391   3.2551717456
H   -3.6931752239  -0.1653635490   4.6630072451
H   -2.6869149318  -1.4441116972   5.3149647923
C   -1.6746767297  -0.1609684173   3.9257346434
H   -0.9749855524  -2.6834416569  -2.1219731228
C   -2.1076764240  -4.4378178957  -2.5862247751
C   -3.8487815176  -5.0077527836  -1.0304228977
H   -4.0450645690  -3.7268323846   0.6705864564
C    0.4344621056  -3.6028856118   1.0031268989
C   -0.8768306715  -4.4467660147   3.0096209591
C   -1.9961314924   0.8061212688  -5.7247675392
C   -2.0381512388  -1.6081336811  -6.4686795877
H   -1.9260134363   2.7832631498  -4.9178566667
C   -2.9211366715   3.5306293283  -2.4213678904
H   -2.4192450867  -4.3329879681   4.5965633465
H   -5.9293445186  -3.1393897214   2.4021430267
H   -6.2393666460  -4.0953318057   3.8440442646
H   -4.8070414234  -4.4109842985   2.8760862822
H   -0.8275106283  -0.8331226655   3.8190736441
H   -1.3895421599   0.6161350455   4.6335186567
H   -1.8321575999   0.3193820430   2.9621517003
H   -1.5621232575  -4.6331538268  -3.5002151029
C   -3.1499655673  -5.2723748816  -2.2023024594
H   -4.6627600665  -5.6522324896  -0.7252199384
H    1.1591292318  -4.4181956551   1.0512610439
```

N	0.5575728967	-2.5999662572	0.2126071679
H	-0.2510639845	-5.2729442083	3.3053024825
C	-1.5475306742	1.2313361459	-7.0871057369
H	-2.7016459570	-1.4550146052	-7.3211320911
H	-2.2363900163	-2.5978029295	-6.0639476330
H	-1.0213875687	-1.6067841200	-6.8647976493
H	-3.5047797614	3.9421220764	-3.2429342181
C	-2.6113865300	4.3286350365	-1.3006585928
H	-3.4166416886	-6.1251438471	-2.8128050344
C	1.5247052759	-2.4601969185	-0.7653757417
H	-2.2568932149	0.9282495834	-7.8584119222
H	-0.5906868933	0.7783778631	-7.3519331082
H	-1.4317302409	2.3110447485	-7.1447758615
C	-2.9559432019	5.6163129464	-0.9201471594
N	-1.8277594837	3.7745993888	-0.2825514221
C	2.2961273511	-1.2930870386	-0.8239687712
C	1.6700691805	-3.3982729306	-1.7938109939
H	-3.5621710549	6.2949770774	-1.4971420169
C	-2.3874455160	5.8447870423	0.3377891217
C	-1.6922538689	4.7019056170	0.7194276266
P	-1.1313310833	2.1441564774	-0.4744792005
C	3.1046846735	-1.0652877920	-1.9246876807
N	2.3326618771	-0.3588214767	0.2720031485
C	2.4931556753	-3.1786542964	-2.8891191455
H	1.0770176442	-4.3013973192	-1.7440340429
H	-2.4741521391	6.7417127992	0.9264509779
C	-1.0374715836	4.4421846287	2.0386546228
C	0.1884620973	2.5339462163	-1.7155215933
N	-0.0296942034	2.3643036670	1.0029949377
H	3.6838277687	-0.1558233371	-1.9556308031
C	3.2089386423	-1.9720403751	-2.9708736893
C	1.2613004124	0.3884582093	0.4000323744
B	3.6918062859	-0.2975706106	1.1719255089
C	2.5915177894	-4.2051191820	-3.9731616118
C	-0.2371043082	5.6901368387	2.4844719844
C	-2.1566139685	4.1919967442	3.0944835128
C	-0.1186129795	3.2581504940	2.0138413827
C	0.5711420585	1.5481095136	-2.6215886674
C	0.8248370455	3.7715802381	-1.7407983388
C	0.9645500452	1.4104643832	1.3212978580
C	4.0787646178	-1.6605887766	-4.1469511954
H	0.4964512991	0.1992964135	-0.3410673745
C	3.4084993632	-0.9968576456	2.6171155733
C	4.8508194837	-1.1884522261	0.4314475810
C	4.1617311298	1.2631386349	1.2436752551
H	2.2833706266	-3.7986728248	-4.9377025905
H	1.9653561631	-5.0679840292	-3.7586329681
H	3.6163431232	-4.5574214381	-4.0993658848
H	-0.9336377795	6.5228229964	2.5705529208
H	0.1295008781	5.5029795264	3.4926155251
C	0.9157280138	6.0723978749	1.5778689341
H	-1.6665820915	3.9389071679	4.0347389375
H	-2.6751109210	5.1383371575	3.2512740087
C	-3.1527302334	3.1153823009	2.7150686184
C	0.7933390437	2.8912372626	3.0125937429
H	0.0861090947	0.5820532886	-2.6128138252
C	1.5787653822	1.7978051951	-3.5422463926
C	1.8276229602	4.0214379484	-2.6658837468
H	0.5409271536	4.5426463426	-1.0386030151
C	1.4719318757	1.7649622997	2.5776794657

```
H    4.8608631641  -2.4097119481  -4.2796453079
H    4.5624983813  -0.6940026955  -4.0286671645
H    3.5084126029  -1.6373315716  -5.0773458351
C    2.1539920906  -1.4036830129   3.0778355729
C    4.4938206984  -1.2808345995   3.4601490309
C    5.9361662960  -0.6259710733  -0.2432876954
C    4.7955846614  -2.5884506050   0.4572264840
C    4.8501332506   1.7941121894   2.3379755765
C    3.9722227567   2.1276732223   0.1586363859
H    1.6362887921   5.2622526524   1.4780930157
H    1.4449760120   6.9351942508   1.9815749289
H    0.5692648217   6.3369139438   0.5801225618
H   -2.6528547324   2.1957715545   2.4221646277
H   -3.7866845363   3.4252389398   1.8857187380
H   -3.8029514694   2.8779768444   3.5564249454
H    0.9424541669   3.4084982339   3.9448580982
H    1.8723150608   1.0229439864  -4.2370690626
C    2.2087784780   3.0345376580  -3.5673020904
H    2.3162679823   4.9865758024  -2.6781719547
H    2.2324804061   1.2299988375   3.1094882328
C    1.9818093875  -2.0358544085   4.3054014477
C    4.3382958262  -1.9036900626   4.6908930497
C    6.8956866948  -1.4047853154  -0.8838219084
C    5.7362871389  -3.3794534577  -0.1846005859
C    5.3177962973   3.1033672972   2.3583603418
C    4.4298975112   3.4385326404   0.1635015821
H    2.9952768799   3.2286740221  -4.2843807627
C    3.0736263775  -2.2853358313   5.1239478001
C    6.7949398880  -2.7881620508  -0.8669378415
C    5.1082476754   3.9376525572   1.2685349708
H    6.0341811784   0.4511786957  -0.2857956695
H    3.9845907941  -3.0743865752   0.9855312651
H    7.7205183229  -0.9276995174  -1.3991855070
H    7.5345944632  -3.3999059080  -1.3684061952
H    5.6476411913  -4.4586211735  -0.1537612858
H    5.4960968252  -1.0316570304   3.1338655350
H    5.2063485242  -2.1023119078   5.3076569188
H    2.9442531406  -2.7795722711   6.0787800345
H    0.9905530396  -2.3372388278   4.6189122547
H    1.2789178671  -1.2362786036   2.4675002875
H    5.0130619546   1.1809146308   3.2142734617
H    5.8408417115   3.4736535867   3.2317870690
H    5.4659478845   4.9596808636   1.2797364512
H    4.2523046039   4.0731443486  -0.6955937645
H    3.4367464453   1.7781298507  -0.7148657753
```

```
1a-N-B(C6F5)3
  160
G(CENSO)=        -5571.04346296  G(xTB)=         -292.21105228         !CONF2
C   -0.1516578545  -1.0460609321   1.4661987794
C   -0.4389443982  -0.4976540712   0.2113371778
C   -0.7509217460  -0.9300195002   2.7304614536
N    0.9629796052  -1.9084646266   1.6208111674
H    0.3811645706  -0.5062516293  -0.4918365336
N   -1.5514340956   0.0083248589  -0.2881330415
C   -0.0235779936  -1.6925969665   3.6215047415
H   -1.5930809627  -0.3123964016   2.9702534593
C    1.0207039614  -2.3045320052   2.9103209687
```

P	2.3309735267	-1.9861507822	0.3377838878
C	-1.3828813640	0.5178006551	-1.6379372574
B	-3.0414276303	-0.0430959972	0.3745410382
H	-0.2214695270	-1.8153501697	4.6725323570
C	2.0151816600	-3.2693469047	3.4770002685
C	1.3838543661	-2.9895382123	-0.8949125859
N	3.1493318737	-3.3115787532	1.2070069383
C	-1.9367559200	-0.1671387352	-2.7087726957
C	-0.6849210257	1.7114533794	-1.8811309387
C	-3.2188654556	-1.5355218617	1.0783672959
C	-3.3265023835	1.1918914248	1.4419588974
C	-4.1188386464	0.2364567428	-0.8573458031
C	2.9139224711	-3.8621103175	2.4405253936
C	1.2503279354	-4.3930009294	4.2176428766
C	2.9048942952	-2.5183193575	4.5119902797
C	1.1026511852	-2.4243598702	-2.1366620604
C	0.9698027890	-4.2901994525	-0.6223732768
C	4.1639544044	-4.0421424241	0.5789036088
H	-2.4370090556	-1.1008133087	-2.5222335949
C	-1.8832187115	0.3078843825	-4.0125623745
C	-0.6799071171	2.2144288451	-3.1876041684
N	0.0819519122	2.3011459589	-0.8949567285
C	-3.7063280146	-1.7835166963	2.3590901809
C	-2.8394901725	-2.6928502831	0.4009853775
C	-4.6453550580	1.4875621292	1.7836988286
C	-2.3942086891	2.0555899462	1.9972425233
C	-4.1764877503	1.5015313382	-1.4414418842
C	-5.0153400215	-0.6743191798	-1.4032050345
C	3.7716605816	-4.9463403454	2.5940528134
H	1.9853021767	-5.0503582256	4.6803724987
H	0.6996763530	-3.9346007626	5.0377469871
C	0.3071433814	-5.1974540176	3.3459932256
H	2.2422377547	-2.0608016837	5.2465692838
H	3.4955184542	-3.2665332189	5.0411364193
C	3.8185790861	-1.4726507561	3.9054096380
H	1.4252659253	-1.4172112639	-2.3621966812
C	0.4194770529	-3.1556609906	-3.0977547918
C	0.2959199053	-5.0222179450	-1.5886916058
H	1.1777924592	-4.7361430849	0.3400687360
C	4.6142434259	-3.6088034942	-0.6842560175
C	4.5474065330	-5.0622432090	1.4355681970
C	-1.2635223585	1.5443992006	-4.2521912604
C	-2.4927218162	-0.4875135177	-5.1234042906
H	-0.1566943418	3.1435747121	-3.3663686232
C	-0.0006095935	3.5348308486	-0.5506416247
C	-3.8070382735	-3.0478565277	2.9236321650
F	-4.0763485291	-0.7852567958	3.1915811865
C	-2.9206800160	-3.9711021061	0.9235061318
F	-2.3739448679	-2.6231989681	-0.8634150529
C	-5.0230723041	2.5600319843	2.5701063441
F	-5.6551896078	0.6888834620	1.3670118347
C	-2.7268651232	3.1401638675	2.7996241978
F	-1.0757724150	1.8864154801	1.7872504758
C	-4.9825648169	1.8271378360	-2.5171711818
F	-3.3961722936	2.5071148294	-0.9871103529
C	-5.8392455338	-0.3915452467	-2.4836644211
F	-5.1461920323	-1.9255289435	-0.9113292054
H	3.8285189230	-5.5723316920	3.4677757903
H	0.8411523549	-5.7175784424	2.5522519835
H	-0.4467423375	-4.5649957508	2.8797114482

```
H   -0.2144455445  -5.9479283668   3.9390259563
H    4.3115610835  -0.8933205969   4.6850874421
H    3.2657399982  -0.7738853165   3.2817294150
H    4.5943876127  -1.9238588872   3.2887413709
H    0.2080956780  -2.7094974807  -4.0599501717
C    0.0194609602  -4.4569997301  -2.8280941943
H   -0.0193945233  -6.0336689997  -1.3704029238
H    5.3781029517  -4.1699048047  -1.2188867066
N    4.0889192271  -2.5211786243  -1.1316551298
H    5.3169785304  -5.7886019827   1.2329038904
C   -1.2057244046   2.1261048787  -5.6290304319
H   -3.2667246010   0.0773625082  -5.6455133110
H   -2.9465933228  -1.4013405771  -4.7473335851
H   -1.7518264668  -0.7658268207  -5.8749224962
H   -0.7837625281   4.1999082223  -0.9221676769
C    0.9067291976   4.0750948010   0.3958235610
C   -3.4083988537  -4.1545279733   2.2041365197
F   -4.2695546992  -3.2005186931   4.1762614623
F   -2.5537356130  -5.0403933776   0.1982934966
C   -4.0516698037   3.4013402282   3.0845640442
F   -6.3171947632   2.7853051276   2.8567748964
F   -1.7699287499   3.9387275279   3.3042543534
C   -5.8165668688   0.8652268091  -3.0560170964
F   -4.9512786265   3.0590895687  -3.0541876162
F   -6.6683754311  -1.3285943623  -2.9755461807
H   -0.5092887506  -5.0292863549  -3.5790022916
C    4.2325076876  -1.9956589298  -2.4039863536
H   -0.6745436752   1.4690938074  -6.3196647314
H   -0.7014228792   3.0894671655  -5.6295347549
H   -2.2042554754   2.2689396133  -6.0444360567
C    0.8325184865   5.2668712108   1.1010916684
N    2.0019881234   3.3558784779   0.8891653920
F   -3.4946719877  -5.3826800208   2.7314008583
F   -4.3929343549   4.4447554576   3.8541841474
F   -6.5988764338   1.1522464071  -4.1051712962
C    4.2823883034  -0.5935870902  -2.5644269165
C    4.1340001632  -2.8058095322  -3.5373630924
H    0.0686564705   6.0161322265   0.9707152361
C    1.8740907877   5.2728371458   2.0368422183
C    2.5913078821   4.0928883924   1.8855631531
P    2.6889136933   1.9749979023  -0.0693372062
C    4.2094713274  -0.0787594700  -3.8599079564
N    4.3363667736   0.2856420425  -1.4904396249
C    4.0717579177  -2.2836099385  -4.8204645652
H    4.0383360456  -3.8744951588  -3.3949603098
H    2.0720734862   6.0358575978   2.7697620267
C    3.6849357269   3.5674797865   2.7589983219
C    3.2265377960   3.0458725103  -1.4800159624
N    4.2126138437   1.9211430077   0.8830117856
H    4.2431488667   0.9944034431  -3.9762005117
C    4.1061772276  -0.8858010779  -4.9838930324
C    5.1895000625   0.1290501342  -0.5369745128
C    3.9195570273  -3.1952976174  -5.9969270307
C    4.6060347868   4.7187331954   3.2267377049
C    3.0302520232   2.9515957382   4.0368238619
C    4.5068493319   2.5082255029   2.0889115234
C    2.6856970166   2.7988953055  -2.7370575476
C    4.1067304802   4.1093093526  -1.3100532128
C    5.1833007682   0.9429156254   0.6185403410
C    4.0057382682  -0.2674952864  -6.3425952819
```

```
H    5.9769054270  -0.6248714892  -0.5792654973
H    2.9891794670  -3.0045485647  -6.5347746840
H    3.9163255894  -4.2380688079  -5.6883511096
H    4.7265910791  -3.0623269426  -6.7189977548
H    3.9913701612   5.4349415395   3.7704972098
H    5.3006575134   4.3097619203   3.9596319827
C    5.3759587203   5.4294128526   2.1327114094
H    3.8360866627   2.5240444404   4.6334140125
H    2.6119518146   3.7753314432   4.6165139728
C    1.9608570241   1.9108793054   3.7758993785
C    5.6666474301   1.9227978939   2.5849606818
H    2.0196662423   1.9619280451  -2.8817560410
C    3.0080334514   3.6159775222  -3.8108912680
C    4.4409863913   4.9181828410  -2.3870883575
H    4.5337690359   4.3104217124  -0.3386132104
C    6.0873730871   0.9523518445   1.6741732163
H    4.0304805336   0.8179405689  -6.2825931277
H    3.0799191937  -0.5540737335  -6.8440323786
H    4.8210139638  -0.5863334490  -6.9938565295
H    6.0389193671   4.7488521672   1.6001751103
H    5.9922852156   6.2212292291   2.5581940973
H    4.7086329280   5.8881957442   1.4053802407
H    2.2910763818   1.1595523006   3.0616126601
H    1.0524762730   2.3603144001   3.3837093949
H    1.6998631613   1.3898550943   4.6963220172
H    6.1474543822   2.1908621345   3.5100597012
H    2.5786312038   3.4157506100  -4.7838707012
C    3.8891856345   4.6759589720  -3.6396951445
H    5.1307429204   5.7400715336  -2.2464452836
H    6.9575601367   0.3203294924   1.7464796615
H    4.1455422783   5.3105537407  -4.4778950808
```

```
1a-P-BH3
  130
G(CENSO)=       -3390.20123441  G(xTB)=        -184.29450418        !CONF89
C   -1.6047861149   2.2970233493   0.7971823720
C   -0.4462434181   1.6096450697   0.3131259850
C   -1.9914237818   3.6141887772   0.9922290997
N   -2.6567497524   1.4930754092   1.2733450658
H   -0.4415200342   0.5649374867   0.6174873189
N    0.6041431693   1.9052670589  -0.3476305656
C   -3.2626302273   3.6079415043   1.5870275636
H   -1.3946317047   4.4794096928   0.7595571285
C   -3.6662375462   2.2876495322   1.7428163561
P   -2.7047426683  -0.2505248765   0.8902976480
C    1.0302732851   3.0441270345  -0.9796637942
H   -3.8246446439   4.4724345820   1.8952225913
C   -4.8886436407   1.7333205990   2.4124261263
C   -2.6560046515  -0.0369386046  -0.9277366309
N   -4.3817232139  -0.4815926180   1.2546628632
C    0.2116960852   3.9249719047  -1.6909110113
C    2.4372226679   3.2122760589  -1.0428358102
C   -5.2577143241   0.3710702965   1.8927303466
C   -6.0793601067   2.6971696477   2.2357480308
C   -4.5958203622   1.5332293121   3.9386827799
C   -3.7649813697   0.5370748270  -1.5459838484
C   -1.5358154209  -0.3663653035  -1.6829969549
C   -5.0266123581  -1.6998498896   0.9963309363
```

H	-0.8579724877	3.7688252556	-1.6683981995
C	0.7315472977	4.9576325420	-2.4569109832
C	2.9497445724	4.2666280688	-1.8036291539
N	3.2054015526	2.2858649547	-0.3517602969
C	-6.4584827070	-0.3062587055	2.0335726809
H	-6.9064254122	2.3166133698	2.8338165139
H	-5.8104729293	3.6528862870	2.6791809071
C	-6.5337401893	2.9152851912	0.8048651208
H	-5.5129016601	1.1434396196	4.3815821055
H	-3.8475787021	0.7462981730	4.0260736700
C	-4.1322684125	2.7529332052	4.7095595645
H	-4.6381728433	0.8072259980	-0.9697560092
C	-3.7513656378	0.7672308521	-2.9135761288
C	-1.5253136937	-0.1231622013	-3.0478419618
H	-0.6693582223	-0.7967917649	-1.2116154272
C	-4.3619227537	-2.7111043063	0.2637204617
C	-6.3200099827	-1.5850924958	1.4743967114
C	2.1296830371	5.1401639792	-2.5038330484
C	-0.1848739468	5.8568019476	-3.2248354938
H	4.0215194160	4.3952689891	-1.8751748995
C	4.4344832745	2.4590128821	-0.0018747035
H	-7.3460875117	0.0977589118	2.4882685177
H	-5.7382286043	3.3312617317	0.1894314361
H	-7.3691504990	3.6145850042	0.7777884583
H	-6.8652193583	1.9888123731	0.3390576740
H	-3.1815732267	3.1310369677	4.3392432996
H	-3.9929206342	2.4854085233	5.7570845398
H	-4.8538573267	3.5672206139	4.6771032841
H	-4.6173482372	1.2105578912	-3.3862714620
C	-2.6317994164	0.4399197562	-3.6680768447
H	-0.6493338559	-0.3833662507	-3.6250885762
H	-4.9382495716	-3.5802402224	-0.0514907263
N	-3.1207825179	-2.5359962332	-0.0429937569
H	-7.0758308558	-2.3498273270	1.4058193823
C	2.7288591860	6.2446069059	-3.3163599088
H	-0.0942757745	6.8951830829	-2.9016328990
H	-1.2230452349	5.5583757830	-3.0997278147
H	0.0409741183	5.8455384163	-4.2922025560
H	5.0018799312	3.3699625178	-0.1965817314
C	5.0794830944	1.4572619866	0.7574201301
H	-2.6217329194	0.6272859668	-4.7335000467
C	-2.4751145238	-3.2324115119	-1.0617877745
H	3.8134369691	6.2477258599	-3.2371310342
H	2.3675140829	7.2224459359	-2.9943908502
H	2.4713700812	6.1534820903	-4.3729696406
C	6.3297017809	1.4615159596	1.3633038157
N	4.4038544754	0.3113050371	1.1915077818
C	-1.0775977847	-3.4509341774	-1.0232751831
C	-3.1628476333	-3.5349520045	-2.2417880972
H	7.0762184081	2.2279477699	1.2339902392
C	6.4066620890	0.3321525726	2.1825445624
C	5.2045133799	-0.3600676573	2.0800795605
P	2.7566396397	-0.0921990065	0.6144161650
C	-0.4627218208	-3.9636734495	-2.1709625316
N	-0.3465878433	-3.2521285020	0.1391434632
C	-2.5433814028	-4.0508748161	-3.3705698655
H	-4.2163435643	-3.2929416102	-2.2923606320
H	7.2296559695	0.0548947569	2.8183771126
C	4.7424174155	-1.4870402763	2.9472864945
C	3.1958433916	-0.6111399369	-1.1083798793

```
N    2.8111712414  -1.7944003117   1.3212434962
H    0.6010218952  -4.1583605616  -2.1262112827
C   -1.1550176265  -4.2664329508  -3.3350335003
C    0.7918349768  -2.6413182128   0.0896776841
C   -3.3378921060  -4.3324439310  -4.6069816766
C    5.8755381628  -2.5285478794   3.1089116127
C    4.4272321770  -0.8983665298   4.3551405162
C    3.5146853168  -2.1723130351   2.4320341460
C    4.2700415345  -1.4635353122  -1.3501549068
C    2.3914303347  -0.2015654429  -2.1685600845
C    1.7125955971  -2.6625846726   1.1797870891
C   -0.4249662926  -4.7971220615  -4.5277452664
H    1.0990589629  -2.1094451943  -0.8124079274
H   -3.2697793934  -5.3812907888  -4.9001487598
H   -2.9779226433  -3.7499314941  -5.4566575705
H   -4.3894150905  -4.0955104532  -4.4624647881
H    6.7320762900  -2.0297656679   3.5616342213
H    5.5421800428  -3.2693668752   3.8343711736
C    6.2955475824  -3.2146276024   1.8246670083
H    4.1454567117  -1.7299304559   5.0014881595
H    5.3535540693  -0.4852141047   4.7550111666
C    3.3388557354   0.1568424656   4.3696036240
C    2.8978574609  -3.2867368225   2.9915279509
H    4.8960174642  -1.7916924316  -0.5320798787
C    4.5378429516  -1.9012739819  -2.6387293599
C    2.6574659447  -0.6457024550  -3.4564765953
H    1.5600810380   0.4597391932  -1.9739310092
C    1.7771954195  -3.5866788422   2.2174759880
H   -0.5397508756  -4.1397782301  -5.3911799912
H   -0.8045691150  -5.7740910339  -4.8308626389
H    0.6385639696  -4.8988742882  -4.3252888342
H    5.4682860679  -3.7618731498   1.3744457602
H    7.0960979214  -3.9282269548   2.0183264214
H    6.6625802996  -2.4989746979   1.0902848958
H    3.6200517189   1.0376595567   3.7937311031
H    3.1397988000   0.4835565352   5.3899227150
H    2.4038112064  -0.2247035685   3.9597453577
H    3.2550334284  -3.8296736905   3.8503171144
H    5.3754859014  -2.5622726579  -2.8183031164
C    3.7289781966  -1.4968230397  -3.6946056823
H    2.0267486901  -0.3261901452  -4.2753785900
H    1.1009899548  -4.4161771397   2.3401997394
H    3.9343508229  -1.8446559440  -4.6985415818
B   -1.6319603426  -1.0424682716   2.3142105965
H   -0.4635775205  -0.8821105637   2.0940771612
H   -2.0342010947  -0.3194319129   3.2003498957
H   -1.9632556149  -2.1883814829   2.4421749786
```

```
1a-P-BPh3
160
G(CENSO)=       -4082.85423534  G(xTB)=         -228.70181477         !CONF57
C    1.5837589750  -3.1602309038  -0.2280411906
C    1.9407880517  -2.2658559701   0.8260541796
C    2.3245194934  -4.0464488735  -1.0114659227
N    0.2520968069  -3.2370638726  -0.6628323599
H    1.1383052380  -1.6179738824   1.1639533196
N    3.0528760415  -2.0613396494   1.4310676097
C    1.4484723933  -4.6394281672  -1.9223716966
```

H	3.3802740619	-4.2306406033	-0.9187862989
C	0.1724241394	-4.1225624451	-1.6875142042
P	-1.0211211213	-2.0472753382	0.0161433023
C	4.2349863425	-2.7486904586	1.1836103300
H	1.7029030602	-5.3639219538	-2.6781002945
C	-1.0695397941	-4.3787536210	-2.4872365310
C	-1.1823416269	-2.9378248370	1.6360876555
N	-2.3287616961	-2.8476668971	-0.8875209040
C	4.4474428271	-4.0155811974	1.7268313935
C	5.2882220664	-2.1067214992	0.4997879049
C	-2.3072048749	-3.8190895692	-1.8545257025
C	-1.2402460838	-5.8989183943	-2.7153878950
C	-0.8962056281	-3.7013226681	-3.8805539491
C	-1.6443704298	-4.2489882791	1.7028951478
C	-0.7949523011	-2.2918756836	2.8082978011
C	-3.6597002438	-2.5002866290	-0.6279790874
H	3.6286837469	-4.4847021405	2.2582692776
C	5.6573439540	-4.6831685869	1.6033623181
C	6.4996184362	-2.7997165810	0.3642691339
N	5.0275683294	-0.8609130557	-0.0723171352
C	-3.6271906359	-4.0878206824	-2.2103305121
H	-2.0865918528	-6.0493048237	-3.3848083969
H	-0.3646764963	-6.2519868373	-3.2587984353
C	-1.4264625996	-6.7197035164	-1.4549856276
H	-0.0393971311	-4.1698929342	-4.3656835489
H	-1.7744438610	-3.9485882032	-4.4784600262
C	-0.7059793325	-2.1985086155	-3.8351782328
H	-1.9411712256	-4.7658230353	0.8013066540
C	-1.7268287744	-4.9020247861	2.9244417401
C	-0.8736263537	-2.9471785848	4.0290332801
H	-0.4329407253	-1.2738660380	2.7751056566
C	-3.8890526280	-1.4614886455	0.2975583100
C	-4.4690207202	-3.2713870599	-1.4475744086
C	6.7085047461	-4.0602518084	0.9016883933
C	5.8291918886	-6.0461563808	2.1957987459
H	7.3043167849	-2.3490023106	-0.2011747904
C	5.9620273098	-0.0971980829	-0.5294009924
H	-3.9332248469	-4.8035929778	-2.9536703794
H	-0.5793552799	-6.6144497536	-0.7782320117
H	-1.5212689508	-7.7775000908	-1.7006967715
H	-2.3248700949	-6.4274437337	-0.9133130667
H	-1.5689964131	-1.6931264278	-3.4030460332
H	-0.5665246721	-1.8031402322	-4.8411062741
H	0.1703188577	-1.9191870448	-3.2510923393
H	-2.0910788648	-5.9202162501	2.9635742802
C	-1.3411449236	-4.2531157532	4.0914209185
H	-0.5704056291	-2.4332845300	4.9315874824
H	-4.8977453673	-1.1334542599	0.5352192839
N	-2.8277488420	-0.9284434824	0.7958740652
H	-5.5446134451	-3.2247363765	-1.4841156291
C	8.0267021060	-4.7488721859	0.7248626990
H	6.6380347253	-6.0659939183	2.9289997114
H	6.0844702145	-6.7865657134	1.4352793350
H	4.9198395700	-6.3781350796	2.6926339716
H	7.0261611414	-0.3043116535	-0.4041272231
C	5.6616341746	1.0503905547	-1.3020614988
H	-1.4031116480	-4.7637574817	5.0434287389
C	-2.7318058185	0.0243282221	1.7990542423
H	8.4902260689	-4.9859195518	1.6845623294
H	8.7233910468	-4.1276735372	0.1658352078

H	7.9218729607	-5.6949042862	0.1905730854
C	6.5317100985	1.8566431925	-2.0236801716
N	4.3593490451	1.4395747300	-1.6337226995
C	-1.8090898880	1.0772699703	1.6340129514
C	-3.3642523907	-0.1454114667	3.0269261552
H	7.6062446309	1.7755681046	-2.0032830490
C	5.7620035692	2.7216666191	-2.8098797476
C	4.4235792044	2.4535810521	-2.5625554652
P	2.9560750311	0.8679802671	-0.6975062955
C	-1.5551599669	1.9029387630	2.7306585616
N	-1.2915891564	1.3466906048	0.3746387466
C	-3.1186513931	0.6924775614	4.1080468877
H	-4.0329280941	-0.9882256497	3.1457691897
H	6.1307155674	3.4429833913	-3.5185999046
C	3.2372664178	2.9658467316	-3.3144033546
C	3.3088788329	1.7800939236	0.8728926472
N	1.8201145558	2.0859403099	-1.3967246962
H	-0.8689294798	2.7289006400	2.5969263859
C	-2.1896270375	1.7354829345	3.9564582575
C	-0.0335389107	1.5589762267	0.2018277546
C	-3.8169407399	0.4657388816	5.4109788215
C	3.4120108984	4.4708202219	-3.6302105779
C	3.1625426921	2.2030024949	-4.6725681569
C	1.9484050953	2.7607627793	-2.5828013182
C	3.5058948441	3.1598393443	0.8761814319
C	3.3146695684	1.0795159664	2.0759663643
C	0.4649273245	2.0812501298	-1.0293278305
C	-1.8916790440	2.6697248182	5.0860831038
H	0.6823504551	1.3607677319	1.0026209446
H	-3.1106409869	0.2273724642	6.2084968582
H	-4.5272366943	-0.3548161915	5.3412306715
H	-4.3619415736	1.3527159494	5.7358502439
H	4.3149213114	4.5868729405	-4.2287218306
H	2.5861500562	4.7688714035	-4.2747749678
C	3.4700633099	5.3805449406	-2.4196371463
H	2.3364869899	2.6351945241	-5.2379359238
H	4.0756384026	2.4249748335	-5.2259835470
C	2.9764439667	0.7040362948	-4.5538241578
C	0.6898764815	3.2047696444	-2.9690299138
H	3.4918493218	3.7107303687	-0.0529360796
C	3.7097321049	3.8319212264	2.0707845921
C	3.5181164807	1.7571836645	3.2718140704
H	3.1698794839	0.0060068392	2.0694025956
C	-0.2293757103	2.7804332212	-2.0077983689
H	-2.7811535583	3.2259246156	5.3877330163
H	-1.1258766920	3.3901874758	4.8086444523
H	-1.5447579430	2.1356966681	5.9718115517
H	2.5574822358	5.3163547941	-1.8285024436
H	3.5889219044	6.4185756880	-2.7296785580
H	4.3089229181	5.1325833245	-1.7712355800
H	3.8243283312	0.2249602180	-4.0665010600
H	2.8709857952	0.2567528900	-5.5422568096
H	2.0801103788	0.4532949746	-3.9858607003
H	0.4813491380	3.8036350527	-3.8393531614
H	3.8627603294	4.9030299178	2.0652124835
C	3.7136717414	3.1313757769	3.2728253745
H	3.5208789812	1.2076224666	4.2041284839
H	-1.2793952530	3.0095169353	-1.9654276733
H	3.8683882707	3.6578274073	4.2056559889
B	-4.7328538808	3.1638059057	0.0998337878

```
C    -5.3559476049   3.2514834728   1.5263851600
C    -5.4198726199   4.4685893437   2.2251226049
C    -5.8837516282   2.1192805761   2.1676728720
C    -5.9855498242   4.5531610056   3.4879958823
H    -5.0356196828   5.3670940091   1.7609926039
C    -6.4320619825   2.1921319991   3.4382847263
H    -5.8399616139   1.1610461178   1.6704764766
C    -6.4869393832   3.4113610703   4.1018415187
H    -6.0323717100   5.5067557557   3.9976377265
H    -6.8114111632   1.2981526617   3.9152843399
H    -6.9193923031   3.4721679021   5.0923551041
C    -5.2159781717   2.0737332861  -0.9071553864
C    -4.3214273954   1.4830381958  -1.8134279634
C    -6.5506278451   1.6409777317  -0.9482647008
C    -4.7308311230   0.4987093150  -2.6995389736
H    -3.2838028355   1.7839805516  -1.8023097280
C    -6.9737717255   0.6801383892  -1.8550388643
H    -7.2725046914   2.0794427503  -0.2717435319
C    -6.0614537073   0.0997179146  -2.7279249927
H    -4.0146556713   0.0435206952  -3.3710603190
H    -8.0126191743   0.3771287738  -1.8773886064
H    -6.3857626968  -0.6617411047  -3.4255958678
C    -3.6394864965   4.1849360877  -0.3446532282
C    -2.6584078818   4.6505514872   0.5434108358
C    -3.6039265772   4.6801955148  -1.6572257363
C    -1.6796432069   5.5451656553   0.1391841613
H    -2.6486686248   4.2817992252   1.5596833022
C    -2.6545418981   5.6095034228  -2.0563470843
H    -4.3440666128   4.3425482641  -2.3711940286
C    -1.6824090387   6.0368615548  -1.1606527559
H    -0.9171850052   5.8646493093   0.8377354228
H    -2.6603454439   5.9883280273  -3.0701398846
H    -0.9246806089   6.7423708323  -1.4764881975
```

```
1a-P-B(C6F5)3
160
G(CENSO)=        -5571.01082315  G(xTB)=         -292.17408454          !CONF16
C     3.2400422858  -1.7475830598   2.4292092394
C     3.2457257221  -0.3199541413   2.4331340888
C     4.2520118803  -2.7038438656   2.5202574560
N     2.0314155589  -2.4389829645   2.2632336249
H     2.2865075202   0.1351786866   2.2058211042
N     4.1851248441   0.5313246852   2.6214199708
C     3.6581520761  -3.9614470578   2.3895298186
H     5.2982673640  -2.4948460427   2.6598782672
C     2.2834236620  -3.7715381161   2.2310414972
P     0.4572293909  -1.4901697781   1.9408009555
C     5.5036789658   0.2129806523   2.9256981804
H     4.1638434482  -4.9126391678   2.4014331736
C     1.2457774248  -4.8127364997   1.9331356819
C     0.2716802801  -0.9474798880   3.7059043613
N    -0.5164355829  -2.9780722808   1.9932261617
C     5.8757794440  -0.0525365172   4.2434226712
C     6.5004683366   0.3049651521   1.9327822195
C    -0.1576073828  -4.2994027413   2.0505476095
C     1.4402682441  -6.0290701152   2.8665559797
C     1.4526536858  -5.2998058779   0.4655867578
C     0.0809686615  -1.8670775917   4.7328835396
```

```
C    0.3681491273   0.4078460128   4.0130945267
C   -1.9131265855  -2.8879638048   2.0066320965
H    5.0937797912  -0.1100164129   4.9901411435
C    7.1980786964  -0.2371296266   4.6199467397
C    7.8319868649   0.1023963853   2.3237980358
N    6.0856591220   0.5069705534   0.6160676070
C   -1.3296888925  -5.0512850543   2.1009887596
H    0.7421887272  -6.8068113162   2.5592657054
H    2.4351247542  -6.4315779137   2.6804064096
C    1.2718604445  -5.7370054492   4.3444168671
H    2.4518782078  -5.7324137839   0.4070546926
H    0.7436251583  -6.1090617452   0.2867118650
C    1.2914481390  -4.2252151864  -0.5905359824
H    0.0112363235  -2.9224229066   4.5101111227
C   -0.0205242313  -1.4369684052   6.0481382847
C    0.2694353710   0.8367746256   5.3292751494
H    0.5145769497   1.1346324620   3.2266777417
C   -2.4801727922  -1.6011730320   1.8923611336
C   -2.4212555444  -4.1763835956   2.0766449750
C    8.2013676313  -0.1540540571   3.6348162440
C    7.5421288054  -0.5217732835   6.0474575121
H    8.6124091012   0.1275541085   1.5756355044
C    6.9105142076   0.7444811985  -0.3480644444
H   -1.3744086363  -6.1257964581   2.1447104173
H    1.4400823955  -6.6390126849   4.9325531580
H    0.2684656007  -5.3794621719   4.5704036601
H    1.9796035999  -4.9835218875   4.6871767759
H    0.2814948077  -3.8167203861  -0.6056375018
H    1.4919366139  -4.6344716579  -1.5802254512
H    1.9824652591  -3.3986626501  -0.4317151952
H   -0.1709058862  -2.1607810093   6.8383137552
C    0.0738518823  -0.0835926193   6.3501373795
H    0.3441760913   1.8923669170   5.5543756542
H   -3.5585323520  -1.4638998797   1.8827998468
N   -1.6303512423  -0.6417907553   1.7670931533
H   -3.4670051739  -4.4353325574   2.0945704137
C    9.6431503812  -0.3488681031   3.9893900026
H    8.0829934835  -1.4636886471   6.1513423772
H    6.6486155693  -0.5797204264   6.6647084257
H    8.1883271848   0.2515742307   6.4669526784
H    7.9777488848   0.9203170677  -0.2044559680
C    6.4843978192   0.7187766039  -1.6969473774
H   -0.0024991648   0.2509273910   7.3763460358
C   -1.8377648806   0.7281589565   1.7433421019
H    9.8278452244  -1.3367184047   4.4151667877
H    9.9759809975   0.3753041199   4.7349350323
H    10.2810892217  -0.2399103766   3.1151104381
C    7.2560570345   0.7681597032  -2.8504382866
N    5.1727409496   0.4275858599  -2.0837355819
C   -1.1087535171   1.4874086425   0.8043855378
C   -2.5600110092   1.3792617757   2.7386581883
H    8.3192438955   0.9431119900  -2.8728664367
C    6.4196347041   0.4892055384  -3.9371953684
C    5.1366723492   0.2796434634  -3.4514919921
P    3.8188722664   0.5665650339  -0.9334122144
C   -1.0996300803   2.8744268184   0.9483462032
N   -0.5486051781   0.8462157080  -0.2924537302
C   -2.5760843610   2.7648297200   2.8516714851
H   -3.0758435937   0.7766497022   3.4750263927
H    6.7151090640   0.4077631767  -4.9690474108
```

```
C    3.9556932751  -0.2523353956  -4.1965041916
C    3.8359250295   2.4083001038  -0.7571153823
N    2.5441265452   0.4906954533  -2.2155734281
H   -0.5543312815   3.4592520923   0.2189977270
C   -1.8150661041   3.5257388744   1.9477950843
C    0.6731340749   1.0424011168  -0.6447633018
C   -3.3659391731   3.4210554045   3.9399654547
C    3.8749601746   0.3843598668  -5.6040114829
C    4.1601502651  -1.7879134982  -4.3828387434
C    2.6623234000  -0.0312597061  -3.4775933873
C    3.7764944488   2.9708616586   0.5148138744
C    3.8450834160   3.2353910429  -1.8784314883
C    1.1842674968   0.5027191072  -1.8637500780
C   -1.7750480843   5.0184821382   2.0430450710
H    1.3512877017   1.6240647081  -0.0167020585
H   -2.7246024022   3.9838137553   4.6207417976
H   -3.9103143148   2.6863905063   4.5285606077
H   -4.0910612941   4.1318601462   3.5407200815
H    4.7889629710   0.1329596436  -6.1415309821
H    3.0672301079  -0.1102586643  -6.1418577093
C    3.6590786768   1.8840485423  -5.6251654207
H    3.3316876051  -2.1515756274  -4.9914574484
H    5.0696765563  -1.9308782670  -4.9666699602
C    4.2326472659  -2.5800073639  -3.0937271295
C    1.3899217272  -0.3408840095  -3.9429197228
H    3.7858467275   2.3267230449   1.3850752187
C    3.7223462797   4.3515008679   0.6603828096
C    3.7931477460   4.6120956459  -1.7293955158
H    3.8890599826   2.8054356005  -2.8687991454
C    0.4758511386  -0.0161682542  -2.9401384558
H   -1.4114955779   5.3501341943   3.0167765867
H   -2.7669671086   5.4563654835   1.9176652598
H   -1.1248529698   5.4440002554   1.2822054713
H    3.5809191732   2.2404578510  -6.6522522602
H    4.4840989109   2.4168095777  -5.1550960187
H    2.7409712364   2.1648825202  -5.1106259753
H    5.0924772803  -2.2992209239  -2.4875755902
H    4.3157425033  -3.6453525061  -3.3069745147
H    3.3378326864  -2.4371396705  -2.4893252658
H    1.1622352889  -0.7359839111  -4.9186122106
H    3.6752195207   4.7835644177   1.6513728395
C    3.7278626995   5.1733911980  -0.4582141199
H    3.8015339396   5.2478015576  -2.6048867952
H   -0.5945854110  -0.0984954019  -2.9837781785
H    3.6827658126   6.2485032547  -0.3431322080
B   -4.5230717430   0.6913766310  -2.0488604840
C   -3.4587750063   1.1038488149  -3.1113299899
C   -2.5967873105   2.1885012622  -2.9254683366
C   -3.3126026698   0.4196946985  -4.3210899555
C   -1.6359882253   2.5523453601  -3.8497739898
C   -2.3791327612   0.7747476598  -5.2775894571
C   -1.5337545526   1.8464242826  -5.0377351811
C   -4.8868046940  -0.8169490226  -1.8420730200
C   -6.2087832143  -1.2380728778  -1.7118403682
C   -3.9197119754  -1.8177447969  -1.7704230914
C   -6.5586889388  -2.5617748358  -1.5178149814
C   -4.2324603021  -3.1484447855  -1.5602633463
C   -5.5614468479  -3.5215255897  -1.4357345090
C   -5.2468520512   1.7703849385  -1.1817569167
C   -5.6331614172   3.0078904922  -1.6986398676
```

```
C    -5.5588022682   1.5521330939   0.1610299551
C    -6.2633541951   3.9757952296  -0.9394924641
C    -6.1760981167   2.5035335203   0.9526410831
C    -6.5302442837   3.7224859045   0.3975188970
F    -5.2369081570   0.3931845531   0.7568487098
F    -6.4366231837   2.2603586152   2.2425095775
F    -7.1303479695   4.6479444821   1.1455416891
F    -6.6242856524   5.1469680486  -1.4775819013
F    -5.4195308056   3.2987706767  -2.9929646021
F    -2.6176192183  -1.5104723853  -1.8783300182
F    -3.2717553137  -4.0769598624  -1.4805441055
F    -5.8804404740  -4.8018118498  -1.2416499896
F    -7.8424830964  -2.9273324709  -1.4139514393
F    -7.2144119338  -0.3490539795  -1.8022590365
F    -2.6532848007   2.9185967603  -1.8037770036
F    -0.8005965238   3.5695159654  -3.6091260251
F    -0.6137103712   2.1856950115  -5.9390073460
F    -2.2799853150   0.0969566663  -6.4279403446
F    -4.1159809990  -0.6143764854  -4.6211991088
```

```
1b-N-BH3
  130
G(CENSO)=        -3390.23598384  G(xTB)=        -184.30500456         !CONF12
C    -3.5993770690   0.3414557456  -2.4575465469
C    -2.5177418295  -0.3726603770  -3.0302105800
C    -4.6882904036   0.9282130253  -3.0850564978
N    -3.7210382822   0.5836933692  -1.0842813695
H    -2.6855190533  -0.7807330517  -4.0295514353
N    -1.4022775391  -0.5458274252  -2.4072266971
C    -5.4709478692   1.5359895398  -2.0972195270
H    -4.8646624559   0.9259288416  -4.1483189132
C    -4.8764048044   1.3005809493  -0.8660278349
P    -2.6732541356  -0.2269547278   0.1131074941
C    -0.5042938653  -1.5158671676  -2.8320708460
H    -6.3829706040   2.0844678372  -2.2531654073
C    -5.3454068740   1.7021941610   0.5034108412
N    -3.8816104463  -0.1525153154   1.4195155323
C    -0.9289096385  -2.8362218237  -3.0247203614
C     0.8744499708  -1.2447402536  -2.9185619044
C    -5.0112696053   0.6302656067   1.5058189731
C    -6.8688728540   1.9532080273   0.4916995825
C    -4.6268094307   3.0139793336   0.9575032078
C    -3.8751335802  -1.0496871426   2.4921861480
H    -1.9852059681  -3.0474471171  -2.9190940268
C    -0.0489603014  -3.8816260581  -3.2636430149
C     1.7577594919  -2.3023546195  -3.1382163795
N     1.4168579728   0.0120619167  -2.6763978570
C    -5.7109823358   0.2440532391   2.6395769930
H    -7.1419020039   2.3333395030   1.4757844575
H    -7.0670126895   2.7692753904  -0.1992827128
C    -7.7532866379   0.7718593941   0.1334464496
H    -5.0485943894   3.2801315909   1.9276731428
H    -3.5798294167   2.7834251896   1.1295981086
C    -4.7212343779   4.1896766834   0.0048511289
C    -2.8108981385  -1.9472520005   2.7621441327
C    -5.0146854270  -0.8083996880   3.2454209035
C     1.3302893983  -3.6087375378  -3.3206726925
C    -0.5629005696  -5.2775027451  -3.4193457469
```

H	2.8136658631	-2.0766502094	-3.1731650777
C	1.0320147174	1.1399225832	-3.1787172841
H	-6.6366484908	0.6754386180	2.9776606977
H	-7.6430338481	-0.0510804847	0.8356607660
H	-7.5333349330	0.3866351451	-0.8596390400
H	-8.8004151240	1.0745901741	0.1435736287
H	-5.7466293824	4.5143105568	-0.1638042683
H	-4.2807977200	3.9582985038	-0.9636202833
H	-4.1749170366	5.0382592286	0.4166302496
H	-3.0289990108	-2.7453933783	3.4748217937
N	-1.6476971928	-1.8204291003	2.2211354176
H	-5.2796078179	-1.3318790776	4.1494323548
C	2.3257197045	-4.7032600593	-3.5411006176
H	-0.1697174933	-5.9352783364	-2.6421903583
H	-0.2621614519	-5.7124836940	-4.3733638305
H	-1.6484053758	-5.3081316169	-3.3636264464
H	0.3098392197	1.2300150970	-3.9847100235
C	1.6371331147	2.2829604581	-2.6007107001
C	-0.7128594878	-2.8445317264	2.3490539872
H	3.3405358742	-4.3129810754	-3.5620240849
H	2.1483245186	-5.2285560153	-4.4807213063
H	2.2741240160	-5.4554486038	-2.7519574820
C	1.7805828369	3.6203462445	-2.9342696882
N	2.3628676470	2.0627700192	-1.4289350446
C	0.6059912413	-2.5724707780	2.7234810358
C	-1.0296032347	-4.1618366987	1.9990576645
H	1.3534068242	4.1003488698	-3.7990339412
C	2.6105223004	4.2059057526	-1.9627161542
C	2.9561193871	3.2346312990	-1.0297967979
P	2.2212879000	0.5068740963	-0.6196455101
C	1.5490463722	-3.5874791212	2.7358763753
N	1.0137216456	-1.2468909822	3.1073823843
C	-0.0892702557	-5.1837540990	1.9999144465
H	-2.0387041770	-4.3703729029	1.6680424132
H	2.9316707693	5.2326532828	-1.9401628279
C	3.8684598465	3.3002725034	0.1700708923
C	3.8330126763	-0.2939165170	-1.0279829239
N	2.7124643927	1.2402031813	0.9965774903
H	2.5597220114	-3.3428212698	3.0360109326
C	1.2328443673	-4.8937238074	2.3754963824
C	1.6137348956	-0.5769202722	2.1609490429
C	-0.4838692783	-6.5655788922	1.5830377545
C	5.2680678915	2.7665273413	-0.2829896657
C	4.0557215262	4.7556523346	0.6412608379
C	3.3232460303	2.4249083395	1.2664785242
C	4.4078368947	-1.1713588106	-0.1109318389
C	4.4369848263	-0.1222341772	-2.2722456085
C	2.2737540817	0.6648890214	2.2046008061
C	2.2897271139	-5.9520111334	2.3706314083
H	1.5938689773	-1.0591709244	1.1919445936
H	-1.5337263781	-6.6080503141	1.3032948263
H	-0.3205764599	-7.2886785648	2.3833689786
H	0.1041383309	-6.9085659195	0.7303479231
H	5.1214370486	1.8368982562	-0.8216968504
H	5.6547724411	3.4839036267	-1.0082666472
C	6.2816603280	2.5371855872	0.8206463058
H	4.7918543237	4.7514920546	1.4422200363
H	4.5238758338	5.2940400571	-0.1829190672
C	2.8293713162	5.5171126977	1.1053437881
C	3.3276821195	2.6083414825	2.6507984875

```
H    3.9648412311  -1.3200175848   0.8635879747
C    5.5706840226  -1.8591666579  -0.4313450772
C    5.5997386603  -0.8078140281  -2.5895171810
H    4.0059000452   0.5574040285  -2.9927386455
C    2.6757672343   1.5234119479   3.2334891419
H    2.0323058340  -6.7838882081   3.0280505254
H    3.2474898497  -5.5532530700   2.6966133099
H    2.4267474245  -6.3765915374   1.3744275413
H    6.4931316545   3.4386170145   1.3927201413
H    7.2223222578   2.1982467481   0.3864262419
H    5.9499400397   1.7699375249   1.5183993759
H    2.3984622674   5.0874689169   2.0054533271
H    2.0524943575   5.5489925053   0.3458140464
H    3.1041407676   6.5465080266   1.3367038361
H    3.7571221073   3.4475982522   3.1699242090
H    6.0077327170  -2.5344121912   0.2920296021
C    6.1685973829  -1.6817164982  -1.6713450231
H    6.0607958173  -0.6595218336  -3.5569664861
H    2.5137697268   1.3535708155   4.2818836767
H    7.0738186577  -2.2192521645  -1.9207421268
C   -1.6628859393   1.2290182099   0.6216224953
C   -1.4088129192   1.4690716716   1.9702586095
C   -1.1537338351   2.1017498234  -0.3377435587
C   -0.7062394199   2.5998351076   2.3555602629
H   -1.7826165332   0.7955121169   2.7253970397
C   -0.4500676467   3.2310247965   0.0534766041
H   -1.3307195862   1.9177362250  -1.3865449629
C   -0.2374928191   3.4896427114   1.4000425475
H   -0.5310774373   2.7865462032   3.4059996087
H   -0.0684999571   3.9083642327  -0.6971112835
H    0.2905862110   4.3797256150   1.7052994085
H    1.8580716211  -0.9621584360   5.1992986464
H    0.4495534838   0.3424208248   4.6442101557
H   -0.0476465351  -1.5582489978   5.0755950511
B    0.7965164083  -0.8224920118   4.6051119258
```

```
1b-N-BPh3
  160
G(CENSO)=       -4082.86900780  G(xTB)=        -228.71002777         !CONF10
C    3.3189984105   2.7120267000   0.5471928005
C    2.8981285484   2.9227661443  -0.7885528457
C    3.8822701048   3.5721832933   1.4764371865
N    3.3591309211   1.4185116622   1.0877477498
H    2.8137838998   3.9468291868  -1.1497802199
N    2.7089225428   1.8904598657  -1.5347386492
C    4.2753127817   2.8049535771   2.5786164170
H    4.0038264213   4.6343849100   1.3436010396
C    3.9478840070   1.4777745020   2.3286674059
P    2.6263461650   0.0560435578   0.2209216644
C    2.3095638193   1.8966021159  -2.8597936401
H    4.7484480940   3.1659874690   3.4758648736
C    4.1129359209   0.3133145532   3.2634929270
N    2.8277357262  -1.1034323564   1.6235485085
C    3.0067031285   1.0474436540  -3.7265850665
C    1.2120616181   2.6260471363  -3.3697000486
C    3.7718956468  -0.9958572154   2.6233460966
C    3.0985896189   0.5609923491   4.4324887244
C    5.5481435533   0.2909358812   3.8303182346
```

C	2.5869619132	-2.4660317900	1.3954207802
H	3.8374498480	0.4932136494	-3.3164603907
C	2.6764169548	0.8923575719	-5.0618266285
C	0.8739860401	2.4435285080	-4.7206701459
N	0.3884921733	3.3447893651	-2.5189721419
C	4.1268557214	-2.2711832125	3.0316539060
H	3.3397418753	1.5363229632	4.8549725367
H	2.1147253687	0.6557692975	3.9798283681
C	3.0434922013	-0.4700527817	5.5410076020
H	5.6893519898	1.2026869661	4.4102278924
H	5.6182267400	-0.5275457754	4.5432250719
C	6.6513642866	0.1586482987	2.7996791037
C	1.6626925681	-3.0086124883	0.4610366756
C	3.3962531338	-3.1867814238	2.2612290372
C	1.5751626259	1.6075179094	-5.5721095559
C	3.4490175541	-0.0536378106	-5.9247895374
H	-0.0135149477	2.9366662368	-5.0942432804
C	-0.3524908388	4.3183737264	-2.9419646061
H	4.8038380698	-2.5104164445	3.8333424613
H	2.7648550469	-1.4546928585	5.1729461415
H	2.2917109296	-0.1699349908	6.2714389675
H	3.9897217983	-0.5645302619	6.0711275903
H	6.6650477387	1.0051460162	2.1157968129
H	6.5373998622	-0.7463186574	2.2054767931
H	7.6244182742	0.1131738366	3.2886920212
H	1.7455420888	-4.0885805689	0.3184774268
N	0.7700522730	-2.3138271465	-0.1551743561
H	3.4080732255	-4.2617661113	2.3426115759
C	1.1380064299	1.4351647558	-6.9919988488
H	2.8071276111	-0.8386058158	-6.3286343169
H	4.2488585582	-0.5323386299	-5.3653246891
H	3.8938695470	0.4529264847	-6.7825179244
H	-0.1636493521	4.8416655422	-3.8799713352
C	-1.4931557208	4.6977573729	-2.1980328804
C	0.0266469203	-2.8633376166	-1.1888585541
H	0.2731580596	2.0545939716	-7.2172608291
H	0.8720834046	0.3985288020	-7.2052546937
H	1.9317156702	1.7004129573	-7.6918602670
C	-2.3110676757	5.8169927939	-2.2567559874
N	-1.9798774188	3.8791429693	-1.1763882030
C	-1.3342276299	-2.5580821098	-1.3182448595
C	0.6137851599	-3.6282047819	-2.2047052331
H	-2.1938034894	6.6366146114	-2.9462057048
C	-3.2770533946	5.6885444984	-1.2510077352
C	-3.0606701753	4.4918101491	-0.5804989304
P	-1.2329885330	2.3410982433	-0.7515468026
C	-2.0308313929	-2.9510405004	-2.4465290798
N	-2.0488858091	-1.8975530918	-0.2543738512
C	-0.0825890912	-4.0393753087	-3.3333085421
H	1.6658301167	-3.8666625247	-2.1236182282
H	-4.0619551488	6.3901418298	-1.0299624237
C	-3.8085712115	3.9331490640	0.5989740949
C	-0.3824034403	2.9250576702	0.7770469379
N	-2.7256664239	1.7250455580	0.0352415519
H	-3.0800880175	-2.7024550639	-2.5149200995
C	-1.4322566156	-3.6789661611	-3.4702662316
C	-2.1371300551	-0.5991597792	-0.3900409361
C	0.6148269698	-4.8249748587	-4.3990290549
C	-3.2565036038	4.5177905860	1.9416153377
C	-5.3084794678	4.2930390344	0.4737647392

C	-3.6767356544	2.4396401795	0.6945697618
C	-0.2830771415	2.0657804815	1.8683082606
C	0.1018696854	4.2263857161	0.8798097114
C	-2.8891497822	0.3494239631	0.3311529348
C	-2.2208395201	-4.0648467548	-4.6809928505
H	-1.5598497004	-0.1877243996	-1.2106352558
H	1.6486787405	-5.0263803839	-4.1284604628
H	0.6168678468	-4.2909678063	-5.3508766542
H	0.1230765889	-5.7813257575	-4.5818595269
H	-3.9108407994	4.1419642839	2.7286320438
H	-2.2785214995	4.0807678505	2.1216559038
C	-3.1500709241	6.0255190997	2.0366121257
H	-5.8020641593	3.9581107409	1.3848374359
H	-5.3990885109	5.3766671949	0.4690241001
C	-6.0155336199	3.7155905075	-0.7374027278
C	-4.4579194332	1.5485396849	1.4268870111
H	-0.6508153725	1.0516514349	1.8045847063
C	0.2306615240	2.5292351551	3.0695233550
C	0.6369146829	4.6768639748	2.0769560313
H	0.0360935418	4.9043686296	0.0414947268
C	-3.9685098579	0.2638119009	1.2115606053
H	-1.7859548293	-3.6515955939	-5.5927763729
H	-3.2463301437	-3.7097760653	-4.6112452024
H	-2.2500601751	-5.1472382084	-4.8159582826
H	-2.4583864720	6.4292234425	1.3003398368
H	-2.7723905658	6.2974881317	3.0223015766
H	-4.1087979205	6.5249327551	1.9064677750
H	-5.9909651174	2.6268326037	-0.7385789732
H	-5.5670719591	4.0585337926	-1.6683493068
H	-7.0616976648	4.0204751805	-0.7445030309
H	-5.3142910163	1.8100320112	2.0245803075
H	0.2589949223	1.8706315205	3.9247669079
C	0.6894711067	3.8348993216	3.1786640087
H	1.0019990066	5.6924084331	2.1499343909
H	-4.3826412924	-0.6479125997	1.5920970259
H	1.0899564673	4.1917455703	4.1179551197
C	4.1043579782	-0.5472723918	-0.7232824799
C	3.9205737456	-1.5977820335	-1.6197531445
C	5.3665473627	0.0241389460	-0.6000677992
C	4.9875738037	-2.0863654361	-2.3610423763
H	2.9399280237	-2.0269120195	-1.7554706150
C	6.4304326111	-0.4558199827	-1.3506151504
H	5.5239617366	0.8547568612	0.0709084119
C	6.2462615959	-1.5155958437	-2.2301418839
H	4.8294865936	-2.9050391099	-3.0505576151
H	7.4057652898	0.0014828870	-1.2471509639
H	7.0777540909	-1.8900538220	-2.8125285774
B	-2.6926864079	-2.8453767829	0.9008825812
C	-4.2769754217	-3.0526523059	0.5817376170
C	-4.9892312214	-2.3814804021	-0.4154095487
C	-4.9988899357	-4.0053954310	1.3153474758
C	-6.3360937513	-2.6263810043	-0.6629495422
H	-4.4899553257	-1.6402855474	-1.0247990995
C	-6.3451569622	-4.2557631937	1.0879346732
H	-4.4866474116	-4.5876568330	2.0715229230
C	-7.0259257655	-3.5627089196	0.0936455512
H	-6.8449787086	-2.0835055660	-1.4501481535
H	-6.8621766947	-5.0004763138	1.6806486582
H	-8.0742724055	-3.7582241225	-0.0940382545
C	-2.3664652484	-2.1703986009	2.3469469740

```
C    -3.2334094450  -2.2330413924   3.4412536255
C    -1.1199445201  -1.5758802037   2.5811270284
C    -2.8840106933  -1.7390858180   4.6936077407
H    -4.2191175203  -2.6627446315   3.3195956658
C    -0.7526045113  -1.0876806429   3.8272067635
H    -0.4126964690  -1.5032555432   1.7659990876
C    -1.6363135481  -1.1639806176   4.8960422266
H    -3.5909863636  -1.8020333164   5.5119849836
H     0.2257843219  -0.6461548650   3.9619406499
H    -1.3576200548  -0.7811750183   5.8700465789
C    -1.9650090067  -4.3159888326   0.8070020387
C    -2.3561844684  -5.2456853091  -0.1672713636
C    -0.9517446396  -4.7272183183   1.6755463781
C    -1.7514955879  -6.4868562155  -0.2963241709
H    -3.1511204177  -4.9847653884  -0.8531910592
C    -0.3411234169  -5.9735760815   1.5677273929
H    -0.6181549217  -4.0585223503   2.4575068770
C    -0.7313973244  -6.8582652551   0.5736047830
H    -2.0759047007  -7.1675534752  -1.0740723130
H     0.4437339193  -6.2493639182   2.2616337759
H    -0.2567954940  -7.8274482041   0.4816936647
```

```
1b-N-B(C6F5)3
  160
G(CENSO)=        -5571.03269513  G(xTB)=         -292.19062001         !CONF1
C    -5.1678112229  -1.4750291141  -0.9149601814
C    -5.3877874523  -0.8070517028   0.3140593694
C    -6.1172499078  -1.7712452659  -1.8838652400
N    -3.9480251029  -2.0170931415  -1.3387712911
H    -6.4380144954  -0.7006484926   0.5968946927
N    -4.4459148861  -0.3632045827   1.0688150932
C    -5.4786675761  -2.4703512631  -2.9095571398
H    -7.1573829458  -1.4940404260  -1.8286729047
C    -4.1455771302  -2.6285976598  -2.5539573392
P    -2.4118712465  -1.7923333436  -0.4247242141
C    -4.7186317344  -0.0291865915   2.3911368131
H    -5.9317781479  -2.8485271430  -3.8091855614
C    -3.0605963650  -3.3075889820  -3.3353220978
N    -1.5348393060  -3.0821344431  -1.3447740630
C    -5.2398600530  -1.0033755521   3.2464698089
C    -4.3973312260   1.2331314437   2.9290238239
C    -1.9660446849  -3.7926659249  -2.4398428007
C    -2.4702451955  -2.2348018203  -4.3072040740
C    -3.6481072771  -4.4776272710  -4.1483935613
C    -0.4140719960  -3.7293941996  -0.8142290326
H    -5.4798016228  -1.9685943133   2.8223049021
C    -5.3833808041  -0.8076288662   4.6122346012
C    -4.5314080775   1.4190571977   4.3113091112
N    -3.9557582693   2.2364561163   2.0766218755
C    -1.1285295212  -4.8872218530  -2.6057077494
H    -3.3025862953  -1.8575720065  -4.9024005113
H    -2.1250499605  -1.4024164163  -3.6964479637
C    -1.3462726577  -2.6904462730  -5.2156420694
H    -4.3382961154  -4.0600476843  -4.8811595753
H    -2.8431421547  -4.9223353689  -4.7281190984
C    -4.3427229886  -5.5617332441  -3.3441504132
C     0.3714000622  -3.2227689616   0.2479448088
C    -0.1713206304  -4.8559191860  -1.5867296998
```

C	-5.0019407363	0.4296467440	5.1619152264
C	-5.8713841798	-1.9231233441	5.4803698917
H	-4.2269463221	2.3660855267	4.7369123894
C	-4.1736913216	3.4827668178	2.3352977430
H	-1.1966799488	-5.6121353838	-3.3975004603
H	-1.6519410645	-3.4908934642	-5.8869202067
H	-0.4826349934	-3.0368942549	-4.6506083893
H	-1.0187564740	-1.8575804022	-5.8370007918
H	-3.6717911121	-6.0192537480	-2.6196741573
H	-4.6978891463	-6.3512687489	-4.0063327718
H	-5.2031438081	-5.1770030909	-2.8005168804
H	1.1299824589	-3.8991785736	0.6453892320
N	0.2298236972	-2.0246546058	0.6920306770
H	0.6455664600	-5.5419464315	-1.4351693246
C	-5.0784152567	0.6719319903	6.6366481465
H	-5.0977599113	-2.2493908853	6.1784139939
H	-6.1635554078	-2.7855461828	4.8860948159
H	-6.7275164771	-1.6231929157	6.0857504294
H	-4.8362101379	3.8146037448	3.1355354984
C	-3.5713261416	4.4961692038	1.5516758114
C	0.8877284177	-1.6055435066	1.8368581324
H	-6.0947487225	0.5480182008	7.0134411451
H	-4.7487764478	1.6770851511	6.8885909433
H	-4.4557455814	-0.0323693069	7.1912631236
C	-3.8481191817	5.8520751393	1.4673368228
N	-2.5857623228	4.2115017498	0.6066797401
C	1.5448206201	-0.3716380093	1.8927950845
C	0.7852460927	-2.3410826549	3.0234069612
H	-4.5718938954	6.3754376025	2.0699789893
C	-3.0393565217	6.3899365086	0.4557852806
C	-2.2629452291	5.3699605461	-0.0702583211
P	-1.9329886007	2.5924664637	0.3634222734
C	1.9609942302	0.1268263875	3.1173157434
N	1.7959652267	0.4192180990	0.6990430603
C	1.2300772819	-1.8616278797	4.2455887924
H	0.2762664662	-3.2935108429	2.9822350868
H	-3.0093428394	7.4193015019	0.1442244382
C	-1.2625800752	5.4220596447	-1.1949234512
C	-2.8442987383	2.1564805624	-1.1743992948
N	-0.4121377700	3.2022117872	-0.3589084745
H	2.4397560242	1.0930159733	3.1465733909
C	1.8084163169	-0.5837136829	4.3021074044
C	0.8692018889	1.3351937523	0.5182700901
C	1.0579921709	-2.6882846268	5.4801077561
C	-2.0364483372	5.1883848014	-2.5345314929
C	-0.6093415133	6.8226177354	-1.2443001886
C	-0.1985578930	4.3784569266	-1.0062064212
C	-4.2259768234	2.3313484227	-1.2395441073
C	-2.1691090408	1.6175789800	-2.2655436968
C	0.8060449103	2.4784647471	-0.2906726040
C	2.2756003450	-0.0016816517	5.5981033683
H	-0.0021825763	1.2001573577	1.1489257750
H	0.4305917497	-2.1818150414	6.2152343025
H	2.0136148913	-2.8822652488	5.9690719455
H	0.5972293436	-3.6461921320	5.2510441841
H	-2.6983904672	4.3386271891	-2.4023397410
H	-2.6751704635	6.0609028139	-2.6744450388
C	-1.1824450665	4.9605228953	-3.7663491481
H	0.0501229229	6.8571084036	-2.1086551870
H	-1.4004699849	7.5411658588	-1.4541454976

```
C    0.1577308879   7.2503946373  -0.0067771144
C    1.1468970808   4.4283791276  -1.3768747004
H   -4.7646688458   2.7440216624  -0.4006179081
C   -4.9155742903   1.9955852907  -2.3946405414
C   -2.8616291775   1.3040049362  -3.4253427448
H   -1.1005043194   1.4749651887  -2.2266088102
C    1.7620128464   3.2661975570  -0.9401835539
H    3.0512600977  -0.6182414566   6.0557219470
H    1.4652219492   0.0651183062   6.3256478728
H    2.6836415010   0.9964980621   5.4580823335
H   -0.5830220744   4.0563486460  -3.6789730173
H   -0.5080221523   5.7904487937  -3.9704126274
H   -1.8254709905   4.8416256175  -4.6381306936
H    0.5870607344   8.2404453381  -0.1585732123
H    0.9758936061   6.5703889122   0.2205046511
H   -0.4827372429   7.3011160764   0.8712432824
H    1.6277759182   5.2480185614  -1.8801963509
H   -5.9869873400   2.1376484137  -2.4365815493
C   -4.2351229998   1.4877574950  -3.4928730436
H   -2.3255935230   0.9189676491  -4.2793521723
H    2.8068773633   3.0436432188  -1.0165823578
H   -4.7726250520   1.2348426756  -4.3967906023
C   -2.8168848674  -2.8553556101   1.0310584318
C   -2.4521193629  -2.4241587672   2.3009851835
C   -3.4317190808  -4.0953262835   0.8740495649
C   -2.6867452188  -3.2302244233   3.4054581334
H   -1.9869108690  -1.4582675447   2.4289450550
C   -3.6771609908  -4.8947367552   1.9802158895
H   -3.7177544145  -4.4381839198  -0.1113702496
C   -3.2995576366  -4.4654261392   3.2484920115
H   -2.3972678717  -2.8867694265   4.3892654360
H   -4.1581301835  -5.8554619018   1.8520711842
H   -3.4868587857  -5.0921529321   4.1104700335
B    3.1310094831   0.1153126074  -0.1677906691
C    4.4335235204   0.9031789966   0.4924763670
C    4.3860658213   2.0219423526   1.3125729745
C    5.7263419319   0.4366639113   0.2563225935
C    5.5028272417   2.5988352178   1.9044028635
C    6.8646282050   0.9766810635   0.8249680677
C    6.7549238442   2.0721770289   1.6637519995
C    2.8816432397   0.5634212005  -1.7406459576
C    1.6894728243   0.2626945169  -2.3976838107
C    3.7952470432   1.2468733053  -2.5390448197
C    1.4104615566   0.6083939194  -3.7073083147
C    3.5529250199   1.6183673875  -3.8551104899
C    2.3500924837   1.2989124568  -4.4488097733
C    3.4354528545  -1.5229911370  -0.0375523719
C    3.3001776442  -2.4627320925  -1.0580506139
C    3.9570954459  -2.0651684355   1.1397582231
C    3.6127910769  -3.8087803285  -0.9239349423
C    4.2591780616  -3.4042759763   1.3219919616
C    4.0824373475  -4.2924470969   0.2794919496
F    3.2195651195   2.6428313953   1.5990567276
F    5.3738490052   3.6723301048   2.7041556762
F    7.8456408819   2.6157680689   2.2211717233
F    8.0787276458   0.4661288514   0.5556783330
F    5.9411776535  -0.5912123263  -0.5958050881
F    4.9952684462   1.6546917899  -2.0724848154
F    4.4804550866   2.2958247330  -4.5532895211
F    2.0976751730   1.6428364461  -5.7183411929
```

```
F    0.2477948149   0.2577342593  -4.2808409864
F    0.7251317951  -0.4368963695  -1.7720724184
F    4.2569087690  -1.2833102454   2.1975705072
F    4.7374521333  -3.8444307646   2.4986828642
F    4.3672319211  -5.5926466643   0.4307867559
F    3.4492004329  -4.6526245419  -1.9575824714
F    2.8674664675  -2.1309824480  -2.2927759714
```

```
1b-P-BH3
  130
G(CENSO)=        -3390.20231675  G(xTB)=         -184.30031379         !CONF6
C   -1.4626989620   2.5772551091  -0.4710581169
C   -0.5214684888   2.0250406401  -1.4002232232
C   -1.4784426659   3.7699400724   0.2396028240
N   -2.6306291519   1.8716205627  -0.1015579478
H   -0.7883306531   1.0541397168  -1.8001040791
N    0.5944455977   2.4850501431  -1.8337293137
C   -2.6386925576   3.8000099071   1.0183011120
H   -0.7319052818   4.5400671079   0.1734829240
C   -3.3419427837   2.6264816182   0.7942241474
P   -2.8927538488   0.1483570049  -0.4818809726
C    1.1173667869   3.7344801325  -1.5064232951
H   -2.9702272471   4.6133928106   1.6413711164
C   -4.7307239539   2.3184005177   1.2677165000
N   -4.5042755444   0.0326342133   0.1512987192
C    0.6738144552   4.8794025834  -2.1683021324
C    2.1787439756   3.8475747512  -0.5887014024
C   -5.2438155335   0.9882655771   0.8134304776
C   -5.6772419348   3.4214986782   0.7131033015
C   -4.7769134346   2.3736693736   2.8165273220
C   -5.3107343650  -1.0899340126  -0.0843653345
H   -0.1428618064   4.7747229731  -2.8712326278
C    1.2468882791   6.1267674003  -1.9648080235
C    2.7543538875   5.1091818155  -0.3916012515
N    2.6377217001   2.6933007629   0.0393778320
C   -6.5175012962   0.4752100113   0.9938341461
H   -6.6778469223   3.2219407842   1.0954166014
H   -5.3658859839   4.3719928383   1.1450596518
C   -5.7068049038   3.5199762749  -0.7982623868
H   -4.4606076686   3.3713017257   3.1209342874
H   -5.8187560991   2.2727037537   3.1199586561
C   -3.9308113097   1.3239978389   3.5066852013
C   -4.7711642615  -2.2779856364  -0.6274754280
C   -6.5630356174  -0.8095015889   0.4353185149
C    2.3158357670   6.2445241661  -1.0559144219
C    0.7420568138   7.3167646659  -2.7180979702
H    3.5999053455   5.1938437474   0.2785887254
C    3.1548388790   2.7096994355   1.2190324528
H   -7.3234630980   0.9879112899   1.4898158083
H   -4.7331192120   3.7912937081  -1.2037095449
H   -6.4189461996   4.2809072013  -1.1163520546
H   -6.0046988367   2.5777027423  -1.2570768115
H   -2.8874332097   1.3843089568   3.2000082979
H   -4.2840692714   0.3182499525   3.2849624154
H   -3.9669954374   1.4548094409   4.5878850324
H   -5.4716848795  -3.0905154678  -0.8097446155
N   -3.5002221165  -2.3431161215  -0.8479909685
H   -7.3997162597  -1.4881947247   0.4281185197
```

```
C    2.9888744784   7.5622605551  -0.8268373529
H    0.3834816614   8.0980060946  -2.0457351329
H   -0.0770329649   7.0451734963  -3.3798816792
H    1.5265159643   7.7696669402  -3.3267397487
H    3.2223808317   3.6123664630   1.8293529451
C    3.6760167301   1.5237928159   1.7991190598
C   -2.8515253091  -3.4793473993  -1.3272908163
H    3.4151227811   7.9642738436  -1.7475289250
H    3.7936326678   7.4719109922  -0.1005786358
H    2.2895223117   8.3135411408  -0.4564289281
C    4.4479777384   1.3595253068   2.9378923773
N    3.5385383958   0.2668930518   1.2023706977
C   -1.4898647540  -3.3743139674  -1.6973216139
C   -3.4685475435  -4.7335014213  -1.4365227611
H    4.7444200384   2.1533368792   3.6037617777
C    4.7863636118   0.0010331674   3.0332779116
C    4.2125949269  -0.6661500289   1.9639244321
P    2.5131234033  -0.0002298850  -0.2087326575
C   -0.8491263260  -4.5081142941  -2.2121797164
N   -0.8834962353  -2.1367776608  -1.7376976363
C   -2.8239465522  -5.8513357655  -1.9367017145
H   -4.4955562269  -4.8461550771  -1.1189474422
H    5.3839448898  -0.4546653165   3.8031941489
C    4.2977260760  -2.1192797409   1.5826358528
C    3.7828809684  -0.0455095567  -1.5389207368
N    2.3140811532  -1.7572612780   0.0746523951
H    0.1780528355  -4.4040278444  -2.5349412833
C   -1.4800144324  -5.7318640511  -2.3468919167
C    0.2552114018  -1.7975779404  -1.2569713160
C   -3.5467388677  -7.1565133506  -2.0452644072
C    5.4942537319  -2.2599648150   0.5849588326
C    4.5872424409  -2.9793432697   2.8291576294
C    3.0243841766  -2.5651524182   0.9221966251
C    3.6216716596  -0.9243906944  -2.6095170294
C    4.8308138886   0.8735205004  -1.5635381979
C    1.1775826185  -2.4555715249  -0.3859846002
C   -0.7408966722  -6.9015453051  -2.9143448131
H    0.5592570576  -0.7859104201  -1.5232826823
H   -4.5749627708  -7.0666269445  -1.7025257890
H   -3.0651669950  -7.9372374705  -1.4542991864
H   -3.5697064968  -7.5186602668  -3.0743990147
H    5.3709179398  -1.5170949218  -0.1987404173
H    6.3946039066  -1.9823639210   1.1348056630
C    5.6704645268  -3.6218559933  -0.0568456031
H    4.7050333241  -4.0105143356   2.5033852002
H    5.5639469935  -2.6806808271   3.2092535963
C    3.5649615491  -2.9248389169   3.9509411965
C    2.3645145455  -3.7840068109   1.0005128502
H    2.8171981298  -1.6464324848  -2.6109647202
C    4.5096883493  -0.8993136379  -3.6750830109
C    5.7163683171   0.8950028664  -2.6311953875
H    4.9735373497   1.5593863306  -0.7418976934
C    1.2244952931  -3.7178548645   0.1919505107
H   -1.2176282390  -7.2752830587  -3.8219430115
H   -0.7126471375  -7.7378544421  -2.2140769478
H    0.2846916160  -6.6369964783  -3.1601799875
H    5.8460295790  -4.4116111719   0.6716293159
H    6.5299306794  -3.6005769480  -0.7270422036
H    4.8012805547  -3.9001504592  -0.6505766880
H    3.8862364278  -3.5599968545   4.7765496781
```

```
H    2.5871760760  -3.2752272780   3.6293043914
H    3.4388629080  -1.9177601730   4.3419611562
H    2.6847610466  -4.6331392187   1.5786697969
H    4.3815466018  -1.5949781334  -4.4934751066
C    5.5587244034   0.0104833947  -3.6902198210
H    6.5324453009   1.6052372827  -2.6329901427
H    0.5095312689  -4.5038489854   0.0281468407
H    6.2504092747   0.0292973021  -4.5219032437
C   -1.8757112137  -0.5365365643   0.9065295843
C   -2.4011599781  -1.5305567805   1.7315934666
C   -0.6383756619   0.0140517054   1.2283138127
C   -1.6952691892  -1.9703489914   2.8403930056
H   -3.3698487812  -1.9552609824   1.5349329470
C    0.0628026392  -0.4264644422   2.3417462971
H   -0.1971031972   0.7902116594   0.6291046322
C   -0.4573622106  -1.4243597460   3.1495709848
H   -2.1257217419  -2.7394569542   3.4672288921
H    1.0203649869   0.0181898104   2.5717678850
H    0.0903586318  -1.7663601933   4.0171819160
H   -2.0411063096   0.0237742625  -3.0283708761
H   -3.9220050356  -0.6044265817  -2.7203274230
H   -3.4760884130   1.3540000375  -2.5521104450
B   -3.0810036845   0.2129911727  -2.4682333906
```

```
1b-P-BPh3
160
G(CENSO)=        -4082.86165883  G(xTB)=         -228.70277247         !CONF35
C   -0.0609195148  -2.7540615892  -0.3051898304
C   -0.3511366707  -1.6789228583  -1.2055366399
C   -0.4530860942  -4.0875449132  -0.2696392379
N    0.8739977554  -2.5798594903   0.7311500299
H    0.2862178911  -0.8076329963  -1.0791017723
N   -1.2205149032  -1.5864499720  -2.1435332439
C    0.2549449818  -4.7185480142   0.7611867701
H   -1.1478063045  -4.5513116654  -0.9462387525
C    1.0676874345  -3.7736131912   1.3725121145
P    1.5522178961  -0.9685253475   1.1241314020
C   -2.1329525860  -2.5945995419  -2.4575955267
H    0.2020560233  -5.7609487669   1.0242799498
C    2.0302382717  -3.9325002112   2.5171538169
N    2.9357032950  -1.6282702022   2.0030031301
C   -1.8381699906  -3.4995634171  -3.4759542175
C   -3.3962392155  -2.6586281302  -1.8383028669
C    3.1438321952  -2.9234657309   2.4236161307
C    2.5990552071  -5.3658940799   2.5361388008
C    1.3096576256  -3.6614098191   3.8794939605
C    4.1226449201  -0.9046488318   2.1454269277
H   -0.8631911917  -3.4377229601  -3.9413542349
C   -2.7428458123  -4.4599781925  -3.9077156479
C   -4.3081595074  -3.6219916467  -2.2872074620
N   -3.7078083740  -1.7245890821  -0.8551087729
C    4.4649351168  -3.0215920912   2.8341975180
H    3.2073532610  -5.4650087995   3.4354263455
H    1.7658184844  -6.0515721119   2.6697156701
C    3.4016496238  -5.7943666119   1.3216228115
H    2.0598669463  -3.7936947384   4.6606035152
H    1.0221197343  -2.6139879639   3.9020912924
C    0.0939487355  -4.5146281663   4.1820089227
```

C	4.1672383987	0.4880963362	1.8808115195
C	5.0776380478	-1.7709790814	2.6543082765
C	-4.0122902443	-4.5184586141	-3.3035102135
C	-2.3723182194	-5.3935660940	-5.0165746647
H	-5.2974599582	-3.6402709455	-1.8485570713
C	-4.4993742813	-1.9847822229	0.1277194213
H	4.9291605856	-3.9053333652	3.2358769235
H	3.7230551257	-6.8299502516	1.4340127914
H	4.2930282688	-5.1869116980	1.1859781441
H	2.8146863637	-5.7302465545	0.4082580843
H	0.3355906460	-5.5725146531	4.2679585764
H	-0.6766864517	-4.4085721939	3.4205669059
H	-0.3404722761	-4.2027466396	5.1319021324
H	5.1453990250	0.9702984847	1.9067387275
N	3.0694989524	1.1152042272	1.6228120355
H	6.0926994821	-1.5028678186	2.8975692683
C	-5.0381119300	-5.5070016770	-3.7638058131
H	-1.3509804668	-5.2231927565	-5.3490061190
H	-3.0283215661	-5.2715204154	-5.8804008204
H	-2.4562203402	-6.4376747860	-4.7114070942
H	-4.9321228044	-2.9712896808	0.3030904457
C	-4.8556677283	-0.9596020534	1.0418125597
C	3.0099865174	2.4869247593	1.3976555876
H	-4.6904082536	-6.5345503215	-3.6456183627
H	-5.2741967327	-5.3811264624	-4.8217373825
H	-5.9641839089	-5.4032651851	-3.2028975673
C	-5.8173336197	-0.9347238789	2.0393164370
N	-4.2931255419	0.3180031638	0.9720265788
C	1.9704636975	2.9836558033	0.5859592312
C	3.8991956965	3.4010218049	1.9726621984
H	-6.4443293722	-1.7661941747	2.3168570574
C	-5.8437373852	0.3628803157	2.5738306208
C	-4.8971572356	1.1272795783	1.9107532715
P	-2.8774140562	0.6585681655	-0.0275299432
C	1.9129547289	4.3544819042	0.3343166746
N	1.1650320159	2.0964140543	-0.1159480602
C	3.8287452129	4.7652093695	1.7326632853
H	4.6675759364	3.0298057171	2.6380028090
H	-6.4944914286	0.7162676068	3.3542127165
C	-4.5680087076	2.5868052190	2.0522744456
C	-3.6901325948	1.4861516715	-1.4645519177
N	-2.3839135965	2.1124744229	0.9025188406
H	1.1351207333	4.7177271967	-0.3250119365
C	2.8174211053	5.2521888837	0.8832813694
C	-0.1022016267	1.9640402945	0.0118736457
C	4.8212639208	5.6951757301	2.3574526433
C	-5.3138554036	3.4091946733	0.9490252563
C	-5.0101462293	3.1131538287	3.4316285079
C	-3.0958032605	2.8118800129	1.8418886985
C	-3.0105048034	2.4989087454	-2.1403778740
C	-4.9211723049	1.0513563203	-1.9517506013
C	-1.0449856171	2.5494161319	0.9170388020
C	2.7081255154	6.7112092638	0.5718787039
H	-0.5411454448	1.2477799826	-0.6798592140
H	4.3372090960	6.4423429195	2.9886765707
H	5.3844207154	6.2476402516	1.6033015695
H	5.5353161146	5.1526156391	2.9727696876
H	-5.1090791601	4.4601482978	1.1579866954
H	-4.8506183502	3.1908524034	-0.0093965375
C	-6.8078842449	3.1794905522	0.8359011936

H	-4.8296729763	4.1880890911	3.4372311246
H	-6.0897931084	2.9984398214	3.5013633652
C	-4.3646407523	2.4846620735	4.6530982707
C	-2.2304093907	3.7101666335	2.4494096268
H	-2.0566357708	2.8563617138	-1.7798875867
C	-3.5637727833	3.0807346151	-3.2719777753
C	-5.4717906948	1.6359356023	-3.0833620152
H	-5.4660772861	0.2730906388	-1.4388893093
C	-0.9602177526	3.5471949233	1.8797042743
H	1.8626978519	6.9115506990	-0.0821449133
H	3.6073964164	7.0850675054	0.0792517408
H	2.5805007920	7.3091804259	1.4756252104
H	-7.0368033241	2.1500615985	0.5665125822
H	-7.2179432833	3.8205599561	0.0554561568
H	-7.3397780819	3.4096162757	1.7574782771
H	-4.7643930396	2.9423477544	5.5583128673
H	-3.2873853131	2.6253422709	4.6631919646
H	-4.5585226421	1.4167527903	4.7146521053
H	-2.4934548338	4.4070988708	3.2261618955
H	-3.0292526882	3.8715233019	-3.7811678673
C	-4.7952388138	2.6507444438	-3.7477795401
H	-6.4345107201	1.2990819501	-3.4442239330
H	-0.0721440463	4.1017217084	2.1238285673
H	-5.2262236308	3.1049766102	-4.6300962622
C	0.4313103632	-0.5555517662	2.5339304360
C	0.9001313122	0.1429082830	3.6458272982
C	-0.9243430152	-0.8699254866	2.4526587957
C	0.0300991355	0.5018687996	4.6647807974
H	1.9472457347	0.3891466049	3.7287123934
C	-1.7919924837	-0.5092050774	3.4736959201
H	-1.3074665151	-1.4182359374	1.6052618394
C	-1.3176672066	0.1775734868	4.5826512677
H	0.4091458879	1.0348654089	5.5267081515
H	-2.8396378760	-0.7655242869	3.3985628838
H	-1.9931365306	0.4571403161	5.3791061444
B	3.4967144949	0.6404802511	-2.8131518276
C	3.2251979684	-0.8860586656	-2.6455210672
C	3.6588819103	-1.5715392890	-1.4996834585
C	2.5527786868	-1.6355226819	-3.6236047772
C	3.4240282529	-2.9261923395	-1.3296231830
H	4.1782895375	-1.0277559413	-0.7242607658
C	2.3383035803	-2.9973515931	-3.4723698641
H	2.2105149682	-1.1464377469	-4.5261639306
C	2.7641944649	-3.6441331296	-2.3184410562
H	3.7448604615	-3.4185913054	-0.4235348575
H	1.8282630351	-3.5536485431	-4.2478274027
H	2.5797273965	-4.7026566599	-2.1890240133
C	2.4885154045	1.5448148573	-3.5859149700
C	1.1134461398	1.2613294949	-3.5940255875
C	2.9109544083	2.6863008751	-4.2857748389
C	0.2060883971	2.0823791789	-4.2439709195
H	0.7464654792	0.3971600710	-3.0592161980
C	2.0116827148	3.4987746957	-4.9600729933
H	3.9642738455	2.9329582282	-4.3122451893
C	0.6537991810	3.2036750075	-4.9320382218
H	-0.8493394133	1.8482490416	-4.2158895244
H	2.3660268979	4.3668802359	-5.5007887849
H	-0.0522546155	3.8439089742	-5.4455032968
C	4.7934908261	1.2559001382	-2.1982557247
C	4.8094411731	2.5513962122	-1.6594610902

```
C    5.9927738042   0.5266375477  -2.1510994214
C    5.9540233824   3.0833983815  -1.0848928753
H    3.9018350980   3.1382672332  -1.6649254996
C    7.1485659087   1.0636532141  -1.6056108026
H    6.0217528794  -0.4728324824  -2.5647261546
C    7.1286908775   2.3421672019  -1.0603819172
H    5.9320625155   4.0766869258  -0.6574743020
H    8.0630327702   0.4846544965  -1.5963458074
H    8.0256313257   2.7592574920  -0.6205710556
```

```
 1b-P-B(C6F5)3
160
G(CENSO)=        -5571.01908963  G(xTB)=         -292.15439551         !CONF82
C   -2.0915754505  -2.5886697863   0.8162276110
C   -2.2493032593  -1.8543182765  -0.3982302883
C   -2.6626766333  -3.7786220835   1.2413946318
N   -1.0655271282  -2.2810867630   1.7222644827
H   -1.4496973226  -1.1488313041  -0.6324877462
N   -3.2384491365  -2.0147425000  -1.2053317954
C   -1.9694902097  -4.2056580959   2.3860154223
H   -3.4426495820  -4.3101210396   0.7218432333
C   -0.9808782470  -3.2744733077   2.6619495624
P   -0.4593839993  -0.6053772350   1.8788271859
C   -3.1710556122  -1.4318177816  -2.4725093156
H   -2.1266387137  -5.1288353830   2.9184433069
C    0.1117837948  -3.3123698473   3.6969714854
N    1.0066290347  -1.1345158110   2.7096138931
C   -2.1105821257  -1.6927073292  -3.3404322482
C   -4.2310602129  -0.6308499846  -2.9386118495
C    1.2050265999  -2.3206855739   3.3864349812
C    0.7041642218  -4.7414252408   3.7255643684
C   -0.5320772153  -3.0040812479   5.0856568001
C    2.1966904668  -0.4086761893   2.6919690334
H   -1.3039035735  -2.3221003983  -2.9856730781
C   -2.0702739860  -1.2032466051  -4.6423105564
C   -4.1995035756  -0.1564457709  -4.2497060488
N   -5.1731687037  -0.1885332944  -2.0242910577
C    2.5410752390  -2.3566751706   3.7664272454
H    1.4821649266  -4.7748617752   4.4844792463
H   -0.0797098568  -5.4144883491   4.0716790978
C    1.2641633555  -5.2320689227   2.4051363899
H   -1.0621441514  -2.0602793257   5.0074912954
H   -1.2908439880  -3.7708727787   5.2465627342
C    0.4033826963  -2.9508614241   6.2764359708
C    2.2453781521   0.8988213750   2.1507278566
C    3.1589626216  -1.1740518629   3.3359752393
C   -3.1447891976  -0.4292656967  -5.1125687257
C   -0.8905669508  -1.4953107888  -5.5153655614
H   -4.9965516457   0.4996736087  -4.5757663582
C   -6.4402201879  -0.0913178720  -2.2238320638
H    3.0114835385  -3.1576557763   4.3093489393
H    1.7212963120  -6.2133817528   2.5267358812
H    2.0257161967  -4.5549529849   2.0264801838
H    0.4922961166  -5.3181127386   1.6423210848
H   -0.1725374529  -2.7577717940   7.1819041055
H    1.1361140585  -2.1510920498   6.1806772454
H    0.9439892481  -3.8836214573   6.4286801829
H    3.2316665730   1.3489017475   2.0361194359
```

N	1.1424073365	1.4854268822	1.8177396873
H	4.1841506649	-0.8807173095	3.4918191189
C	-3.1499251929	0.1218031868	-6.5044456517
H	-1.1806055517	-2.0259196305	-6.4238223360
H	-0.1547928551	-2.1024623640	-4.9929864774
H	-0.3961318841	-0.5775854295	-5.8391543362
H	-6.9415759212	-0.4775664028	-3.1104202395
C	-7.1990820719	0.5217262792	-1.2035782013
C	1.0938272575	2.6530463509	1.0656547117
H	-2.2804853948	0.7539448387	-6.6919942849
H	-4.0413354783	0.7178406323	-6.6875336665
H	-3.1222367403	-0.6725462348	-7.2529548638
C	-8.5626904948	0.6714068834	-1.0025469871
N	-6.5415033857	1.0282431290	-0.0772015734
C	-0.1371126929	3.3410387566	0.9725359083
C	2.1673455919	3.1132509320	0.2933660051
H	-9.3302569981	0.3624898696	-1.6927937499
C	-8.7378155742	1.2582760775	0.2556487783
C	-7.4831007711	1.4702334388	0.8175596919
P	-4.7592825257	1.1141132768	-0.0163478532
C	-0.2280829144	4.4253257540	0.0941194744
N	-1.2129829111	3.0082378219	1.7906209947
C	2.0825221087	4.2186632287	-0.5384237515
H	3.0961558197	2.5631699345	0.3135086823
H	-9.6760577021	1.4893102737	0.7298942350
C	-7.1787695578	1.9046385769	2.2156594329
C	-4.5072081241	2.4516035889	-1.2706712503
N	-4.7808949128	2.2348988623	1.4475481380
H	-1.1735618541	4.9490421331	0.0326420529
C	0.8479303102	4.8820490379	-0.6550877111
C	-2.3641566814	2.8024172473	1.2445992590
C	3.2824772635	4.6800675202	-1.3047772778
C	-8.1167336211	3.0649953702	2.6268090923
C	-7.4702194743	0.6977441406	3.1582001391
C	-5.7548730241	2.3372511994	2.3993711274
C	-3.3965205176	2.3879808570	-2.1078491748
C	-5.3642247565	3.5452711405	-1.3490923160
C	-3.5789534379	2.7209569152	1.9855987787
C	0.6853743710	6.0510335190	-1.5733121020
H	-2.4375308106	2.7065721886	0.1619858922
H	3.0807536324	4.7628275849	-2.3722591743
H	4.1203186391	3.9995376872	-1.1719669338
H	3.6069987493	5.6697225076	-0.9760295205
H	-9.1446047253	2.7093304533	2.5608344830
H	-7.9403385211	3.2728879953	3.6815316297
C	-7.9572078011	4.3361402131	1.8171389404
H	-7.2053196059	1.0052076213	4.1698641465
H	-8.5471432043	0.5259929823	3.1500371623
C	-6.7442506080	-0.5809940914	2.7911646475
C	-5.1986365189	2.9170786036	3.5375810118
H	-2.7248127987	1.5437701628	-2.0495361681
C	-3.1444290121	3.4079364355	-3.0140151774
C	-5.1146072573	4.5620167008	-2.2592322238
H	-6.2255614165	3.6073020730	-0.6982212957
C	-3.8484151975	3.1551542507	3.2791553610
H	1.3694378339	6.8622994520	-1.3182663275
H	-0.3284608657	6.4429212192	-1.5373878540
H	0.9017883849	5.7793040796	-2.6080552139
H	-6.9474522075	4.7371686106	1.8934963353
H	-8.6436162041	5.1030223262	2.1754545451

```
H   -8.1718160093   4.1695083744   0.7625215933
H   -5.6728482082  -0.4207413647   2.6893455243
H   -7.1057982067  -0.9946345474   1.8511656661
H   -6.8917968628  -1.3386342345   3.5597866722
H   -5.7337033638   3.1576002236   4.4409298019
H   -2.2767781899   3.3509510584  -3.6573474346
C   -4.0028192115   4.4968774884  -3.0916905874
H   -5.7869171901   5.4080303007  -2.3160789750
H   -3.1296186788   3.6214753163   3.9317667401
H   -3.8064209533   5.2935882359  -3.7971181042
C   -1.4122285733  -0.1206396241   3.3844181150
C   -0.7906029558   0.5813594947   4.4144088411
C   -2.7576184509  -0.4597636301   3.5016389258
C   -1.4966121250   0.9078510555   5.5623190379
H    0.2513748996   0.8524855116   4.3361571871
C   -3.4542837120  -0.1484162244   4.6605094607
H   -3.2552856777  -0.9988232592   2.7074008546
C   -2.8267407661   0.5329009535   5.6938318261
H   -0.9999298287   1.4433722417   6.3602213862
H   -4.4911817448  -0.4361631657   4.7543977586
H   -3.3737227510   0.7765977655   6.5947358343
B    4.4724908019  -1.0232174446  -1.3726667254
C    5.9652497132  -0.6351625311  -1.1010776071
C    7.0162787220  -1.4145446675  -1.5758018611
C    6.3219581847   0.4989736155  -0.3791584252
C    8.3437362701  -1.0950038774  -1.3545837206
C    7.6373742947   0.8377558754  -0.1190434496
C    8.6541393031   0.0372782595  -0.6169502122
C    3.4802143541   0.0278206799  -1.9488685947
C    3.8850650498   0.9984102701  -2.8692093822
C    2.1190345471   0.0247881649  -1.6316234294
C    3.0058735865   1.8945894585  -3.4482747965
C    1.2220136845   0.9279290747  -2.1675938308
C    1.6681121339   1.8605530379  -3.0912129997
C    4.0289762283  -2.4943702005  -1.1014443123
C    3.1717901614  -3.1866248573  -1.9579509521
C    4.5211730256  -3.2224851778  -0.0168630186
C    2.8208715258  -4.5087782753  -1.7602647394
C    4.1871901610  -4.5442805077   0.2124220278
C    3.3323875158  -5.1908706387  -0.6669713679
F    2.0036857424  -5.1377532395  -2.6132458732
F    2.9997221249  -6.4637917756  -0.4596268275
F    2.6705722478  -2.5855733788  -3.0503889482
F    5.3340953397  -2.6406106635   0.8802721149
F    4.6754339169  -5.2025606124   1.2698907359
F    6.7624466809  -2.5135094790  -2.3101517592
F    9.3273419756  -1.8606493050  -1.8431603828
F    9.9289501513   0.3544566498  -0.3848117171
F    7.9399797973   1.9247811543   0.6018312981
F    5.3688798270   1.2999243845   0.1300823053
F    5.1649692672   1.0710188005  -3.2689954956
F    3.4308339487   2.7876709806  -4.3494572292
F    0.8118471193   2.7252172055  -3.6310818890
F   -0.0663454949   0.9119777802  -1.8189645618
F    1.6331379003  -0.8650718444  -0.7546253475
```

```
1a-NN-2BH3
134
```

```
G(CENSO)=       -3416.86319039  G(xTB)=        -187.06913478        !CONF4
C   -1.0678496180  -1.2685306710   2.4087245047
C   -0.3748348905  -0.0487635370   2.4130418164
C   -0.9971946392  -2.3960778940   3.2429030735
N   -2.0522640506  -1.5394516221   1.4325944830
H   -0.6389066363   0.6654639585   1.6496236399
N    0.5665333710   0.3514859700   3.2321085065
C   -1.9039662702  -3.3298188658   2.7712386315
H   -0.3571922889  -2.4954706399   4.0975820218
C   -2.5411592292  -2.7830105714   1.6518511088
P   -2.2545210603  -0.4663804877  -0.0936543532
C    1.0542954780   1.6843132878   3.0286862259
H   -2.0998173843  -4.3024130145   3.1888472938
C   -3.5496170473  -3.4754666008   0.7891467260
C   -3.0436554574   0.9373769021   0.8177325625
N   -3.7125710975  -1.3579011281  -0.5951071264
C    0.3117199265   2.7777575092   3.4384971904
C    2.3205722580   1.8743670255   2.4727806732
C   -4.2316746388  -2.5526240276  -0.1671853963
C   -4.6005914751  -4.1724344034   1.6854890400
C   -2.8110862795  -4.5755371841  -0.0353284564
C   -4.2106728746   0.7681405787   1.5564731301
C   -2.4222787475   2.1837929538   0.7898578522
C   -4.5465786104  -0.8440551318  -1.5947806885
H   -0.6600915878   2.6050412312   3.8809735361
C    0.7926851465   4.0779787640   3.3041540942
C    2.8167751659   3.1704312959   2.3593263701
N    2.9742019510   0.7773867787   1.9276808711
C   -5.4002578811  -2.7906369373  -0.8836877436
H   -5.2773241853  -4.7294393708   1.0393645619
H   -4.0847510228  -4.9190286186   2.2872298087
C   -5.3890068308  -3.2400578096   2.5838576371
H   -2.3423115369  -5.2530304335   0.6787737528
H   -3.5701109887  -5.1486790018  -0.5685271890
C   -1.7747821068  -4.0499542819  -1.0075756196
H   -4.6963355190  -0.1974112718   1.5905389465
C   -4.7545258908   1.8378649128   2.2528237494
C   -2.9638671961   3.2504489723   1.4920640230
H   -1.5133357694   2.3242229979   0.2213904947
C   -4.1149143787   0.3042477633  -2.2872296183
C   -5.5985159573  -1.7282814221  -1.7708466546
C    2.0741687538   4.2749894272   2.7623749711
C   -0.0490946254   5.2367143761   3.7348806115
H    3.7859470059   3.3109428061   1.9001127088
C    4.1144622848   0.3053406473   2.2872529052
H   -6.0268005698  -3.6585077779  -0.7748958889
H   -5.9305278656  -2.4902134441   2.0088630934
H   -4.7411763799  -2.7184147157   3.2868883164
H   -6.1208643211  -3.8002943553   3.1652223465
H   -1.2463284561  -4.8740760401  -1.4854529137
H   -1.0278979330  -3.4391697965  -0.5045211060
H   -2.2213731981  -3.4475359786  -1.7968808553
H   -5.6640428260   1.6996294910   2.8221867386
C   -4.1323348734   3.0806700496   2.2226249619
H   -2.4715679624   4.2131043641   1.4672229571
H   -4.7021914842   0.7129370478  -3.1059566235
N   -2.9744578423   0.7762451485  -1.9282108034
H   -6.3975437129  -1.6109310550  -2.4839340833
C    2.6329820293   5.6523577305   2.5925737642
H   -0.2811979990   5.8972421364   2.8977062094
```

```
H    0.4615511848   5.8495628765   4.4787997867
H   -0.9894320064   4.9023589194   4.1659582145
H    4.7014549115   0.7141312204   3.1061301629
C    4.5462489928  -0.8431722171   1.5952288291
H   -4.5562162333   3.9123157866   2.7697227317
C   -2.3211218904   1.8732809977  -2.4734973916
H    3.6228427880   5.6233492676   2.1437369614
H    2.7134810453   6.1739066915   3.5472778788
H    1.9941499651   6.2669967204   1.9567192372
C    5.5981552074  -1.7273483021   1.7717103972
N    3.7124032199  -1.3572865928   0.5955548870
C   -1.0547115409   1.6834881408  -3.0291980122
C   -2.8177462105   3.1692220731  -2.3604349064
H    6.3970693365  -1.6098038582   2.4848930447
C    5.4000604882  -2.7899282464   0.8847835725
C    4.2316003820  -2.5521113336   0.1680171696
P    2.2544724309  -0.4658305785   0.0936277730
C   -0.3124096070   2.7770668446  -3.4391394288
N   -0.5665858564   0.3507566115  -3.2323593449
C   -2.0754197051   4.2739129914  -2.7636158441
H   -3.7870092839   3.3095555980  -1.9013583150
H    6.0266189488  -3.6578334762   0.7763450735
C    3.5496720462  -3.4752544579  -0.7881137727
C    3.0438258884   0.9374505956  -0.8183027936
N    2.0522129170  -1.5395274401  -1.4321794760
H    0.6595138260   2.6045601281  -3.8814506668
C   -0.7937880342   4.0771724876  -3.3051479211
C    0.3746557268  -0.0492532293  -2.4130349050
C   -2.6346833013   5.6511473606  -2.5942168911
C    4.6007426743  -4.1724826743  -1.6841305204
C    2.8110783941  -4.5750759635   0.0366449462
C    2.5412520270  -2.7830887334  -1.6511046218
C    4.2110039481   0.7678246772  -1.5566951007
C    2.4225137064   2.1839120622  -0.7911172995
C    1.0678553898  -1.2689202799  -2.4084491792
C    0.0477255407   5.2360803967  -3.7359260510
H    0.6383991280   0.6650651366  -1.6495855440
H   -2.7151979322   6.1724320231  -3.5490628412
H   -1.9961447682   6.2661450816  -1.9584144420
H   -3.6246044257   5.6219354570  -2.1455265252
H    5.2774279483  -4.7292396535  -1.0377447466
H    4.0849914026  -4.9193038772  -2.2856677253
C    5.3892229028  -3.2403742264  -2.5827178385
H    2.3421061828  -5.2526203028  -0.6772780868
H    3.5700779043  -5.1482444668   0.5698508726
C    1.7750105169  -4.0491251720   1.0089393193
C    1.9041906738  -3.3302177522  -2.7704125478
H    4.6966376322  -0.1977606236  -1.5902123391
C    4.7550752303   1.8372113473  -2.2533948001
C    2.9643220726   3.2502233594  -1.4936740650
H    1.5134570056   2.3246603394  -0.2229094850
C    0.9973829981  -2.3966627989  -3.2423804335
H    0.2797648016   5.8966166294  -2.8987377723
H   -0.4630788913   5.8488618177  -4.4797874537
H    0.9881024748   4.9019186910  -4.1670698372
H    4.7414549935  -2.7189749049  -3.2859862364
H    6.1211720065  -3.8007680470  -3.1638146596
H    5.9306461109  -2.4903297438  -2.0078928882
H    1.2462518008  -4.8730429584   1.4868237756
H    1.0283819363  -3.4379861701   0.5059398883
```

```
H    2.2218846773  -3.4468909866   1.7982211397
H    2.1001519505  -4.3028944544  -3.1877774946
H    5.6647208620   1.6986774406  -2.8224786951
C    4.1329483015   3.0800620819  -2.2238939374
H    2.4720412231   4.2129042903  -1.4694142238
H    0.3574230822  -2.4963291626  -4.0970580165
H    4.5569976345   3.9114362884  -2.7712723280
B    1.2131138003  -0.4597534313   4.4183018317
H    1.6557818897  -1.5104031709   3.9838725648
H    2.0941458547   0.2211801739   4.8999546905
H    0.3462355647  -0.6893660414   5.2483622806
B   -1.2127510394  -0.4607363268  -4.4186088024
H   -0.3455966535  -0.6904748666  -5.2483406894
H   -1.6555143010  -1.5113258349  -3.9841225598
H   -2.0936647544   0.2200548140  -4.9006842430
```

```
1a-NN-2BPh3
194
G(CENSO)=       -4802.14255701  G(xTB)=        -275.89311122         !CONF2
C    2.3846952130   1.3274875195   1.1994095834
C    2.4338976571   0.5253441066   0.0455431717
C    3.2447959549   1.4942173554   2.2905153281
N    1.2835176719   2.1881125195   1.4143142343
H    1.4928594205   0.3948371714  -0.4686177197
N    3.4482087251  -0.0791856493  -0.5268126765
C    2.6746145626   2.4183790478   3.1498406188
H    4.1699411858   0.9722932386   2.4297932754
C    1.4709168105   2.8513703696   2.5808394989
P   -0.2288605670   2.1243473714   0.3493831871
C    3.1145927042  -0.8328836783  -1.7086608288
H    3.0875908207   2.7638210482   4.0818811622
C    0.5264421070   3.8634745495   3.1543650601
C    0.5807909326   2.8437286030  -1.1494718342
N   -0.8811422279   3.6210816019   1.0585160889
C    3.3680304066  -0.2810531642  -2.9535336323
C    2.6292235109  -2.1392518290  -1.6257119356
C   -0.4862831259   4.3361489628   2.1602219873
C    1.3344197090   5.0683310663   3.6913293694
C   -0.2265693740   3.2193009719   4.3581057874
C    0.6373501291   2.0800673258  -2.3120164787
C    1.1481583305   4.1148749442  -1.1333038780
C   -1.9205844617   4.3120854590   0.4254981207
H    3.7515125466   0.7260906788  -2.9970149227
C    3.1661639250  -0.9865426296  -4.1322481026
C    2.4616196172  -2.8658569715  -2.8075871356
N    2.2007434306  -2.6529632919  -0.4116824541
C   -1.2732946169   5.4815631748   2.2268968227
H    0.6353845645   5.7536859046   4.1685912557
H    1.9820190557   4.7039404203   4.4872038609
C    2.1578664383   5.8050820738   2.6535342875
H    0.5267632114   2.9272940016   5.0903946064
H   -0.8321122015   4.0006449516   4.8185641031
C   -1.0919545955   2.0305530121   3.9998757613
H    0.1969487456   1.0933008519  -2.3353252538
C    1.2540787878   2.5827988780  -3.4483781075
C    1.7585768959   4.6179947791  -2.2722502059
H    1.1194271688   4.7114091163  -0.2323170493
```

```
C   -2.5719943242   3.6974716167  -0.6628228730
C   -2.1610932803   5.4705345839   1.1459263401
C    2.7195738909  -2.3173000481  -4.0547011326
C    3.4332039980  -0.3342254847  -5.4507942044
H    2.0710028900  -3.8717507198  -2.7349803332
C    2.5903578614  -3.7528712093   0.1281290963
H   -1.2056546544   6.2365377685   2.9906303961
H    2.8916806486   5.1514769416   2.1848317417
H    2.7007258017   6.6294091332   3.1153207033
H    1.5323952674   6.2234627346   1.8665586788
H   -0.5100657629   1.2497841848   3.5165580071
H   -1.9027859866   2.3029718028   3.3298484422
H   -1.5381381419   1.5970419055   4.8941422757
H    1.2936039924   1.9819645230  -4.3462389555
C    1.8141603494   3.8527528590  -3.4316644587
H    2.1954629309   5.6073560454  -2.2531111069
H   -3.3786223146   4.2139447154  -1.1792155610
N   -2.1883263106   2.5070494167  -0.9600563704
H   -2.9205020683   6.1992175776   0.9159292313
C    2.4896365981  -3.1257204277  -5.2917059406
H    2.5320452861  -0.2815514284  -6.0643854406
H    4.1705412248  -0.8897553084  -6.0318207355
H    3.8077715652   0.6779104303  -5.3201168437
H    3.4061717113  -4.3567241056  -0.2639673226
C    1.9304454217  -4.1362681132   1.3132520133
H    2.2939941248   4.2460502650  -4.3180703742
C   -2.6145278004   1.7574815918  -2.0449604255
H    3.4035125208  -3.2332221160  -5.8775061248
H    1.7563453104  -2.6511338234  -5.9453715740
H    2.1272887650  -4.1218074898  -5.0496916408
C    2.1701276772  -5.1198329777   2.2587721730
N    0.8764239602  -3.3411786744   1.7786623419
C   -3.1056203458   0.4631947818  -1.8621829264
C   -2.4429778641   2.2292634513  -3.3491042959
H    2.9374412660  -5.8731692482   2.1919447728
C    1.2676479983  -4.9188244884   3.3082111438
C    0.4718316303  -3.8204903213   2.9982895162
P    0.2292075535  -2.0215045104   0.7747824575
C   -3.3566604932  -0.3295743869  -2.9698676682
N   -3.4453856151  -0.0338968319  -0.5530307965
C   -2.7024336423   1.4408349367  -4.4597421980
H   -2.0488268780   3.2278573539  -3.4803584663
H    1.1943141185  -5.5016780518   4.2096157029
C   -0.5631437616  -3.1727086837   3.8605179071
C   -0.5613965905  -3.0253694255  -0.5608577810
N   -1.2981897184  -1.8769020856   1.8127802278
H   -3.7440593777  -1.3239692749  -2.8111516815
C   -3.1501297471   0.1220722765  -4.2666628194
C   -2.4353087469  -0.5163928305   0.1322855468
C   -2.4736426326   1.9837327158  -5.8343962109
C   -1.3885311737  -4.2586352599   4.5935086580
C    0.1602357941  -2.3145098953   4.9415777925
C   -1.4935484744  -2.2882670834   3.0885299327
C   -1.1318293679  -4.2683597084  -0.3005325430
C   -0.6040067093  -2.5063238159  -1.8521721526
C   -2.3931191986  -1.0702148295   1.4240905265
C   -3.4153288564  -0.7834368582  -5.4266281492
H   -1.4927937265  -0.4979932544  -0.3949455108
H   -2.1392995288   3.0176804661  -5.7985545652
H   -3.3812111270   1.9435367632  -6.4379968145
```

H	-1.7187305811	1.4063403800	-6.3706116976
H	-0.7044783837	-4.8349475955	5.2146063591
H	-2.0609543081	-3.7525298888	5.2841032206
C	-2.1766677654	-5.1855302500	3.6896907999
H	-0.6119072559	-1.8743116937	5.5730682241
H	0.7327838289	-2.9982880712	5.5691283861
C	1.0635257042	-1.2308713218	4.3921949235
C	-2.6957179019	-1.7426293990	3.5537192936
H	-1.1129404359	-4.6757575711	0.7007934590
C	-1.7317024482	-4.9858060562	-1.3244384275
C	-1.2086656479	-3.2238075587	-2.8739648727
H	-0.1634972220	-1.5421541711	-2.0649709642
C	-3.2570276461	-1.0071843589	2.5234639776
H	-3.7794933425	-1.7518959711	-5.0934794522
H	-2.5155659659	-0.9485235514	-6.0216992897
H	-4.1602248724	-0.3610240565	-6.1023780438
H	-2.8999093812	-4.6407164092	3.0848065126
H	-2.7279489119	-5.9145076788	4.2831521292
H	-1.5240611565	-5.7370516984	3.0146246189
H	1.8999897161	-1.6414051687	3.8321945871
H	1.4762247697	-0.6297090027	5.2016452261
H	0.5230953515	-0.5574441198	3.7317460695
H	-3.1098678326	-1.8891097638	4.5363828141
H	-2.1720709000	-5.9515269804	-1.1154780727
C	-1.7729492958	-4.4646629606	-2.6127218886
H	-1.2358569025	-2.8126459553	-3.8735374894
H	-4.1787506235	-0.4621842896	2.5560650336
H	-2.2446390526	-5.0249264073	-3.4090875997
B	5.0304831528	0.0288402938	-0.1354341789
B	-5.0288473065	-0.0412726737	-0.1488120742
C	5.3706560799	1.6061922581	0.1052640033
C	4.7636088707	2.6058324738	-0.6646575861
C	6.3328211706	2.0377895370	1.0219465729
C	5.0853920039	3.9490888430	-0.5270638780
H	4.0023200518	2.3350971407	-1.3847200101
C	6.6677870487	3.3788595162	1.1724461436
H	6.8261173046	1.3147071180	1.6579573079
C	6.0427742127	4.3465916988	0.3980592041
H	4.5840456318	4.6872361522	-1.1399463473
H	7.4137441784	3.6674932300	1.9029653093
H	6.2952895781	5.3931928295	0.5140184944
C	5.3466496012	-0.9269214621	1.1431147231
C	4.3809419057	-1.5496111369	1.9372129920
C	6.6831373520	-1.1913446515	1.4786498480
C	4.7175653695	-2.3729679082	3.0069972255
H	3.3335518344	-1.4003453766	1.7192801004
C	7.0357543181	-2.0041775752	2.5468032622
H	7.4735984705	-0.7666571012	0.8719760192
C	6.0488982073	-2.6021353713	3.3222712071
H	3.9355154422	-2.8408734308	3.5903245170
H	8.0813395079	-2.1790331876	2.7692010368
H	6.3153208390	-3.2433644929	4.1532250982
C	5.9022577540	-0.5374473914	-1.4030610793
C	6.5696413785	0.2929783355	-2.3055573942
C	6.0077383911	-1.9154361855	-1.6342680360
C	7.2781648396	-0.2113029891	-3.3921908000
H	6.5283794270	1.3659179337	-2.1694220101
C	6.7032720214	-2.4350054859	-2.7158755735
H	5.5221187821	-2.6021586254	-0.9522351016
C	7.3421706593	-1.5802639027	-3.6082020484

```
H    7.7787521555   0.4680512073  -4.0714192286
H    6.7493791578  -3.5069062243  -2.8653533224
H    7.8893966967  -1.9785507020  -4.4536019228
C   -5.8983241342   0.2530705063  -1.5070561859
C   -6.0042357158   1.5533908251  -2.0179705731
C   -6.5608987433  -0.7464354236  -2.2228906804
C   -6.6934420589   1.8373280830  -3.1878181938
H   -5.5242320995   2.3676971199  -1.4899249399
C   -7.2626396983  -0.4787405823  -3.3945035388
H   -6.5211628544  -1.7681956991  -1.8681839844
C   -7.3260578204   0.8160708067  -3.8887801509
H   -6.7409444723   2.8556434499  -3.5543164669
H   -7.7592509373  -1.2846542600  -3.9210908512
H   -7.8681373379   1.0307422851  -4.8013894742
C   -5.3292815133   1.1627664949   0.9044118672
C   -6.6601645364   1.5123418367   1.1784320821
C   -4.3515640308   1.9183653315   1.5550412347
C   -6.9959706676   2.5304443402   2.0599358729
H   -7.4588968001   0.9858645699   0.6706675993
C   -4.6707298053   2.9474232203   2.4352535344
H   -3.3076984422   1.7113729752   1.3714149304
C   -5.9971581341   3.2572064157   2.6978217409
H   -8.0378355104   2.7642693862   2.2419235014
H   -3.8786416195   3.5122897788   2.9091767783
H   -6.2505450548   4.0587428011   3.3804708657
C   -5.3889826191  -1.5294314447   0.4161582718
C   -4.7858583476  -2.6758495092  -0.1146393594
C   -6.3682271589  -1.7464239182   1.3893922275
C   -5.1275351054  -3.9557377174   0.3008551983
H   -4.0117251939  -2.5750723428  -0.8647456255
C   -6.7239339432  -3.0210715236   1.8156901543
H   -6.8597208971  -0.8992797359   1.8487941061
C   -6.1016030543  -4.1377498119   1.2747364145
H   -4.6281464984  -4.8129655858  -0.1325759405
H   -7.4835144408  -3.1396483103   2.5788479121
H   -6.3694984202  -5.1332307110   1.6061368301
```

```
1a-NN-2B(C6F5)3
  194
G(CENSO)=       -7778.46491981  G(xTB)=        -402.85999790         !CONF1
C    2.5349136438   1.2527691488   1.3656367736
C    2.6226020112   0.5734607014   0.1453486434
C    3.3523275487   1.3011902305   2.5039791942
N    1.4473528857   2.1307672999   1.6102505156
H    1.7000009351   0.5054780387  -0.4114110304
N    3.6494459644   0.0254093609  -0.4751849100
C    2.7849016086   2.1785370218   3.4059659559
H    4.2385042893   0.7213166945   2.6647679816
C    1.6189520439   2.6975406136   2.8268838468
P   -0.1053335387   2.0777127993   0.6037193880
C    3.2852883173  -0.5951444681  -1.7359558486
H    3.1688415361   2.4383819853   4.3771822185
C    0.7278340861   3.7325869991   3.4366883511
C    0.5788835018   2.8838336400  -0.9080651577
N   -0.7744906805   3.5207566104   1.4006996688
C    3.5582675689   0.0563241356  -2.9294204663
C    2.6907996195  -1.8606198061  -1.7775200691
```

C	-0.3388599227	4.2079022837	2.5052794504
C	1.5991761720	4.9336873515	3.8833799670
C	0.0500528619	3.1291525329	4.7016882544
C	0.6157492673	2.1491644413	-2.0899361828
C	1.0368398097	4.1988184669	-0.8993264232
C	-1.8468043861	4.2205342488	0.8359546853
H	4.0021556343	1.0354472890	-2.8964272534
C	3.2934621558	-0.5110391062	-4.1684243490
C	2.4650659556	-2.4508872829	-3.0247021084
N	2.2253346973	-2.4789793584	-0.6291705983
C	-1.1326762135	5.3406867785	2.6441287700
H	0.9499117100	5.6467038306	4.3890358352
H	2.2967377887	4.5752390332	4.6385528390
C	2.3565517546	5.6198304651	2.7646576143
H	0.8421312779	2.8583745931	5.3999982179
H	-0.5275109095	3.9258411543	5.1705160282
C	-0.8346698658	1.9310993022	4.4279722798
H	0.2488726928	1.1322644822	-2.1077357411
C	1.1096542370	2.7214277092	-3.2530153167
C	1.5138170931	4.7738764576	-2.0673522859
H	1.0191674652	4.7746571840	0.0151686013
C	-2.5452840334	3.6672475094	-0.2564281669
C	-2.0644980958	5.3540720817	1.6019153057
C	2.7547199017	-1.8069169149	-4.2168616972
C	3.5954707417	0.2518237346	-5.4184720392
H	2.0039572301	-3.4286028086	-3.0413800195
C	2.5462055688	-3.6665850699	-0.2560938445
H	-1.0410609244	6.0705945244	3.4295058101
H	3.0298569349	4.9328418847	2.2551195660
H	2.9587051632	6.4369423566	3.1596936105
H	1.6821089449	6.0381547068	2.0197776034
H	-1.2402506580	1.5354092854	5.3584551982
H	-0.2776573520	1.1282914371	3.9473586838
H	-1.6750054877	2.1842002783	3.7854821512
H	1.1371435346	2.1418880325	-4.1650655861
C	1.5533130732	4.0361481082	-3.2452397719
H	1.8639873636	5.7972199915	-2.0566422177
H	-3.3375734161	4.2467327649	-0.7271937815
N	-2.2244923819	2.4796347600	-0.6295536035
H	-2.8386880009	6.0824904730	1.4263225046
C	2.4663936045	-2.4767145399	-5.5218700629
H	4.3493330210	-0.2548090426	-6.0234663958
H	3.9666954716	1.2479618631	-5.1906638852
H	2.7118966090	0.3569967180	-6.0495013190
H	3.3385972968	-4.2459795815	-0.7267949312
C	1.8474383366	-4.2200883674	0.8359783144
H	1.9316927836	4.4866498335	-4.1532439297
C	-2.6896078248	1.8616019572	-1.7782318446
H	2.0495882111	-3.4696102754	-5.3717866299
H	3.3677326123	-2.5769132184	-6.1281473599
H	1.7550070520	-1.9011464696	-6.1163185698
C	2.0645849364	-5.3540017406	1.6015309509
N	0.7744332847	-3.5207942812	1.4000680619
C	-3.2841941116	0.5961427274	-1.7373300099
C	-2.4632178723	2.4522083403	-3.0251365289
H	2.8387866083	-6.0824222761	1.4260190481
C	1.1319404166	-5.3411961127	2.6430198290
C	0.3380952277	-4.2084402323	2.5040621644
P	0.1054589606	-2.0776344425	0.6031058543
C	-3.5566345404	-0.0549223585	-2.9311354665

N	-3.6489279539	-0.0248688663	-0.4769453445
C	-2.7523793391	1.8086505795	-4.2176339847
H	-2.0021035084	3.4299303751	-3.0412993874
H	1.0397704716	-6.0714738994	3.4279885103
C	-0.7294031741	-3.7337461028	3.4348644356
C	-0.5779572715	-2.8831528793	-0.9093794406
N	-1.4478290502	-2.1311120835	1.6087043249
H	-4.0006025876	-1.0340176865	-2.8986421302
C	-3.2911397560	0.5127637515	-4.1698422392
C	-2.6223556265	-0.5731503624	0.1438438057
C	-2.4633846470	2.4788574464	-5.5222841949
C	-1.6011322142	-4.9351316588	3.8800848865
C	-0.0526373961	-3.1312411674	4.7008347235
C	-1.6200575940	-2.6983233736	2.8250491761
C	-1.0358446591	-4.1981722338	-0.9014575955
C	-0.6142172631	-2.1479982795	-2.0909794472
C	-2.5352488361	-1.2529806857	1.3638843586
C	-3.5925452010	-0.2497240677	-5.4202614537
H	-1.6994986123	-0.5049202927	-0.4124590624
H	-2.0466975929	3.4717221624	-5.3716683935
H	-3.3644149961	2.5791927359	-6.1289972189
H	-1.7516559113	1.9035023946	-6.1165305067
H	-0.9522719564	-5.6484750121	4.3857962729
H	-2.2992741146	-4.5771298182	4.6349315704
C	-2.3576646246	-5.6205620910	2.7603682650
H	-0.8452384935	-2.8610875882	5.3987946994
H	0.5246598303	-3.9282548270	5.1694361558
C	0.8321767129	-1.9328758558	4.4287584843
C	-2.7863207542	-2.1795585395	3.4037168684
H	-1.0186389697	-4.7744074269	0.0127964677
C	-1.5123104469	-4.7727159694	-2.0699497410
C	-1.1077066631	-2.7197182565	-3.2545115547
H	-0.2474805720	-1.1310376806	-2.1081124371
C	-3.3532443115	-1.3018247945	2.5017927990
H	-3.9639105999	-1.2459174827	-5.1929301350
H	-2.7086604420	-0.3547497273	-6.0508845638
H	-4.3460775901	0.2571099094	-6.0254932438
H	-2.9601249976	-6.4379054135	3.1544530612
H	-1.6826685911	-6.0384437844	2.0157450443
H	-3.0305880387	-4.9332551206	2.2507583708
H	0.2753645869	-1.1295850277	3.9487267525
H	1.6728259831	-2.1852794970	3.7863891919
H	1.2373206197	-1.5381506088	5.3598430144
H	-3.1707217286	-2.4397311697	4.3746611725
H	-1.8624975602	-5.7960631741	-2.0598463513
C	-1.5513102979	-4.0344617900	-3.2475271160
H	-1.1347507272	-2.1397813728	-4.1663234411
H	-4.2395262956	-0.7220331599	2.6623188712
H	-1.9292908371	-4.4845596670	-4.1559009691
B	5.2282019506	0.1495169368	-0.0884009420
B	-5.2278597119	-0.1493280335	-0.0909719237
C	6.0739155147	-0.2185564114	-1.4701836220
C	6.7963069169	0.6671010637	-2.2612913898
C	6.0939835980	-1.5326346310	-1.9379450585
C	7.4236095483	0.3082048478	-3.4464752255
C	6.7094852840	-1.9342974449	-3.1097004044
C	7.3736783418	-1.0007375211	-3.8834077345
C	5.4968458424	1.6969275065	0.4474241137
C	6.2337571826	2.0505871668	1.5748671945
C	4.9522488100	2.7933784462	-0.2194899322

```
C    6.4142465504   3.3566932184   2.0096458273
C    5.1054659686   4.1095683683   0.1769839812
C    5.8459910064   4.3991586243   1.3080261951
C    5.7006541217  -0.9891590258   1.0186546021
C    4.8811645358  -1.7651045412   1.8253674206
C    7.0576833254  -1.2860244958   1.1400607063
C    5.3489684377  -2.7697909178   2.6634077452
C    7.5657586339  -2.2829342430   1.9518833026
C    6.7008588806  -3.0386484870   2.7243073306
C   -6.0730080098   0.2193183075  -1.4729464747
C   -6.7952164363  -0.6659449986  -2.2646703113
C   -6.0929692303   1.5336245556  -1.9400617162
C   -7.4223047791  -0.3064438963  -3.4497922755
C   -6.7082822631   1.9358935917  -3.1117112414
C   -7.3723694942   1.0027386069  -3.8860019816
C   -5.7011897494   0.9887365867   1.0163786758
C   -7.0583606499   1.2851993952   1.1372579926
C   -4.8823710439   1.7643704440   1.8240620961
C   -7.5671400192   2.2815349718   1.9493556738
C   -5.3509740604   2.7682370655   2.6626602466
C   -6.7028935657   3.0369281721   2.7227956233
C   -5.4967161374  -1.6970335687   0.4439451207
C   -6.2340974332  -2.0512616875   1.5709081308
C   -4.9518667717  -2.7931541353  -0.2232979092
C   -6.4148169854  -3.3575865025   2.0049237336
C   -5.1053205165  -4.1095532831   0.1724041583
C   -5.8463624710  -4.3997117659   1.3029609896
F   -4.2289455230  -2.6199558549  -1.3488596711
F   -4.5591096011  -5.1119908711  -0.5352734871
F   -6.0047800354  -5.6660824563   1.7080380353
F   -7.1211036182  -3.6146701534   3.1189674676
F   -6.7921756632  -1.1263108327   2.3816838812
F   -3.5486030953   1.5798517150   1.8527112690
F   -7.9798448818   0.5647681772   0.4578236188
F   -8.8898490272   2.5121261763   2.0132199594
F   -7.1731913149   4.0010974690   3.5262078471
F   -4.4988715836   3.4782336880   3.4225403450
F   -5.4722672304   2.5198095968  -1.2559612962
F   -6.6568515769   3.2180423149  -3.5111792020
F   -7.9685058370   1.3645208189  -5.0296645326
F   -8.0838228243  -1.2228361379  -4.1770428146
F   -6.9375751483  -1.9668080024  -1.9317227473
F    3.5474618012  -1.5801016644   1.8535891308
F    4.4963359837  -3.4798506953   3.4226187977
F    7.1705468217  -4.0032529355   3.5275576356
F    8.8883925437  -2.5138073456   2.0164176497
F    7.9797066893  -0.5654429003   0.4615129957
F    5.4731386873  -2.5191720800  -1.2544841985
F    6.6580781829  -3.2162344417  -3.5098585985
F    7.9699483718  -1.3619135739  -5.0271920896
F    8.0852627508   1.2249687330  -4.1731279702
F    6.9386289254   1.9677853064  -1.9276343852
F    6.7916103657   1.1252194902   2.3853233988
F    7.1200713250   3.6132176198   3.1241100998
F    6.0041677698   5.6653194223   1.7138524238
F    4.5595121558   5.1123581199  -0.5303933801
F    4.2298724338   2.6207633840  -1.3454893118
```

```
1a-N-GaCl3
  130
G(CENSO)=       -6669.80563814  G(xTB)=        -196.67292831        !CONF5
C   -0.2319854728   2.1287247820  -0.9375398981
C    0.2007415399   0.8816234340  -1.3914280171
C    0.3928645820   3.3883315374  -0.9203321406
N   -1.5157711397   2.2986159183  -0.3671882188
H   -0.4339079139   0.0369271588  -1.1660986068
N    1.2833246699   0.5727643148  -2.0765414107
C   -0.4789021231   4.2881450374  -0.3473807034
H    1.3835585217   3.6140171264  -1.2658397199
C   -1.6564553053   3.5942496360  -0.0202355391
P   -2.5727309811   0.8357679775   0.2066273467
C    1.4685466220  -0.8086582093  -2.4237029376
H   -0.3041230144   5.3372421439  -0.1844471437
C   -2.8647303975   4.1870149137   0.6332024740
C   -2.9789831256   0.2783438898  -1.5080544457
N   -3.9629454975   1.9105851882   0.5093624970
C    0.8466295518  -1.3370571242  -3.5411129146
C    2.3834348644  -1.5785271646  -1.7021181384
C   -4.0471286908   3.2759549849   0.5971615943
C   -3.2104496826   5.5287752004  -0.0544051838
C   -2.5204144615   4.4885098830   2.1246535930
C   -3.5430792018   1.1407223829  -2.4435550274
C   -2.6559080147  -1.0248997148  -1.8803407652
C   -5.2423440682   1.3741357144   0.6798040242
H    0.1678628533  -0.7077209960  -4.1010078467
C    1.0906971691  -2.6413525466  -3.9603673157
C    2.6576702961  -2.8728987821  -2.1403277662
N    2.8667045825  -1.0809761409  -0.5041749064
C   -5.3823565050   3.6081861846   0.8016972077
H   -4.0462373077   5.9739711097   0.4835491021
H   -2.3702599189   6.2048692241   0.0937339921
C   -3.5369916725   5.4258635705  -1.5314397182
H   -1.6878110079   5.1925147355   2.1275834555
H   -3.3783198683   5.0080735842   2.5517351411
C   -2.1709864543   3.2744422396   2.9609254176
H   -3.7859518286   2.1575214597  -2.1667731299
C   -3.7889725106   0.7004971430  -3.7359743007
C   -2.9014928579  -1.4625169142  -3.1735139779
H   -2.2104683524  -1.6996125553  -1.1631622300
C   -5.3575650594  -0.0240583556   0.8004262309
C   -6.1283941836   2.4258063396   0.8493948452
C    2.0272107500  -3.4165264994  -3.2528130694
C    0.3685110794  -3.1951321493  -5.1465601083
H    3.3467146996  -3.4748133065  -1.5634309100
C    4.0924606728  -0.9122146230  -0.1544684255
H   -5.7620186383   4.6081739738   0.9182098547
H   -3.7712132043   6.4097706483  -1.9374917194
H   -4.3973861781   4.7825011704  -1.7091810964
H   -2.6984145968   5.0274484731  -2.1013803130
H   -1.9074142510   3.5762322837   3.9739742044
H   -1.3167068334   2.7391330298   2.5519760097
H   -3.0021277132   2.5753598582   3.0353532918
H   -4.2269167157   1.3766772440  -4.4581198309
C   -3.4695535768  -0.6018015292  -4.1032436303
H   -2.6426363753  -2.4745202700  -3.4548241703
H   -6.3296664622  -0.4868858114   0.9567229840
N   -4.2539582734  -0.6873218956   0.7708504176
H   -7.1881190392   2.3274521907   1.0170512738
```

C	2.3312296283	-4.8208936560	-3.6686826149
H	-0.2504092493	-4.0519283367	-4.8724097955
H	1.0615416761	-3.5452430555	-5.9127550353
H	-0.2795957145	-2.4480633280	-5.5983946696
H	4.9371702923	-1.0805038609	-0.8189065354
C	4.2936310299	-0.4282377310	1.1536667713
H	-3.6577251834	-0.9418393958	-5.1130428541
C	-4.1459895051	-2.0731964135	0.8139806506
H	3.0776225378	-5.2706406825	-3.0184620756
H	2.7070038253	-4.8639334244	-4.6918131647
H	1.4387968489	-5.4479246445	-3.6389266810
C	5.4094212825	0.0065303340	1.8515624995
N	3.1680461157	-0.1919882973	1.9517719224
C	-3.1813548622	-2.6396315898	1.6625560421
C	-4.8694156134	-2.8944778216	-0.0499444104
H	6.4232646442	-0.0298632780	1.4889284073
C	4.9623970267	0.5165856293	3.0741559862
C	3.5768652511	0.3890833062	3.1243137321
P	1.5492434414	-0.6885409933	1.3986562176
C	-2.9629974985	-4.0128973021	1.5869633252
N	-2.5344934496	-1.8489519030	2.6200845249
C	-4.6520964123	-4.2648040810	-0.1174420530
H	-5.5806649771	-2.4310338827	-0.7217918876
H	5.5726563985	0.9495364287	3.8476003193
C	2.6556564150	0.9157385602	4.1790324229
C	1.7340349314	-2.5243109333	1.5596234413
N	0.8189687268	-0.4420787052	3.0815322539
H	-2.2242410759	-4.4490954133	2.2470734799
C	-3.6666519196	-4.8325194233	0.7089465141
C	-1.3019473946	-1.5372232658	2.4258679438
C	-5.4323009278	-5.1049480723	-1.0799960479
C	3.2655802794	0.6662175201	5.5784540267
C	2.5272578238	2.4566450097	3.9823463348
C	1.2860884944	0.3117457422	4.1159667748
C	2.5088721089	-3.0923518667	2.5666071270
C	1.0469295244	-3.3516513231	0.6745964473
C	-0.5173422531	-0.7758431511	3.3382535206
C	-3.3633825643	-6.2964019860	0.6471858571
H	-0.8133852790	-1.8495467086	1.5056442349
H	-5.9786468737	-5.9016115020	-0.5722100993
H	-4.7818012649	-5.5914619938	-1.8091678248
H	-6.1548776340	-4.5050244269	-1.6287356155
H	4.2186454073	1.1910023730	5.6291318940
H	2.6208677929	1.1496299632	6.3112454959
C	3.4561595968	-0.7926700157	5.9428489200
H	1.8896855786	2.8308966391	4.7838304759
H	3.5163107722	2.8910067172	4.1336357921
C	1.9748393617	2.8804949924	2.6368474777
C	0.2624310038	0.4563126135	5.0517296882
H	3.0413093236	-2.4602296045	3.2638085418
C	2.6002681397	-4.4720061314	2.6820365255
C	1.1332073675	-4.7311826375	0.7954121620
H	0.4425447143	-2.9213404058	-0.1120888550
C	-0.8563446342	-0.2238513598	4.5697589991
H	-2.5658714263	-6.5635978085	1.3367298902
H	-3.0545750354	-6.5991975198	-0.3552937620
H	-4.2383474152	-6.8996899763	0.8960869173
H	4.1372422147	-1.2922631657	5.2554013110
H	2.5117758864	-1.3350703348	5.9306220273
H	3.8755007251	-0.8831747607	6.9447746814

```
H    1.8424858941   3.9616192811   2.6005103206
H    1.0036090129   2.4283107653   2.4418386599
H    2.6401699048   2.6062142696   1.8199940325
H    0.3392075938   0.9930675650   5.9822257319
H    3.2074734157  -4.9049123987   3.4661238498
C    1.9119446752  -5.2946608266   1.7976568903
H    0.5931282577  -5.3641664711   0.1040587765
H   -1.8165097142  -0.3224652206   5.0482774202
H    1.9813808969  -6.3704523279   1.8916301675
Ga   2.6654102666   1.7292150387  -2.8733765499
Cl   3.8808429706   2.7232794256  -1.3216809973
Cl   1.6551126740   3.1783346248  -4.1967776187
Cl   4.0205572047   0.4919616640  -4.0909962714
```

```
1a-NN-2GaCl3
  134
G(CENSO)=       -9975.99753515  G(xTB)=        -211.79131470        !CONF1
C    1.4791580004   2.1096131115   1.1915653160
C    1.8228645230   1.5149204564  -0.0228151077
C    2.2368116427   2.4992472877   2.3103022216
N    0.1302006878   2.4177068007   1.4814966528
H    1.0010950251   1.1963991306  -0.6475953433
N    3.0099774552   1.2801382561  -0.5445807314
C    1.3696381604   3.0121179410   3.2510676484
H    3.2980867941   2.3910252720   2.4298489245
C    0.0710014501   2.9580003864   2.7132300157
P   -1.2829479921   1.8263123582   0.3720817637
C    3.0376675251   0.6782338707  -1.8486174873
H    1.6283889841   3.3904064023   4.2246251819
C   -1.1833912887   3.3826377960   3.4086754532
C   -0.8973471720   3.0265707215  -0.9797327814
N   -2.4646835395   2.7761580506   1.3038853729
C    2.9837163931   1.4757761731  -2.9787395365
C    3.2427960038  -0.6953604173  -1.9762247579
C   -2.3646186594   3.4449873735   2.4955389276
C   -0.9600554290   4.7611805779   4.0729689972
C   -1.4863839349   2.3473363964   4.5352943069
C   -0.9062519580   4.4009906199  -0.7610687161
C   -0.5540202764   2.5325809922  -2.2359106774
C   -3.7571292016   2.9591119900   0.7990596301
H    2.8578695739   2.5423829437  -2.8518762706
C    3.1008678128   0.9378413473  -4.2559006349
C    3.3900623845  -1.2356168218  -3.2518644191
N    3.1699297679  -1.4948737587  -0.8423941066
C   -3.5898495581   4.0579298191   2.7436644451
H   -1.8608552660   5.0108417075   4.6311384016
H   -0.1716014785   4.6508977424   4.8158235059
C   -0.6140276421   5.8838475308   3.1154719716
H   -0.6229666224   2.3313970802   5.2005128915
H   -2.3260947713   2.7343318463   5.1129071283
C   -1.7936863505   0.9477425287   4.0447779475
H   -1.1630856866   4.7952373616   0.2122854443
C   -0.5810497920   5.2711947776  -1.7912005847
C   -0.2285728754   3.4048058416  -3.2645325052
H   -0.5387295051   1.4665578361  -2.4156742135
C   -4.1009368577   2.2447475299  -0.3620490432
C   -4.4566444302   3.7576543751   1.6890807245
C    3.3208031566  -0.4447360127  -4.3918209800
```

C	2.9975182260	1.8251644512	-5.4543838368
H	3.5238462379	-2.3047652674	-3.3438426704
C	4.1007933588	-2.2453762230	-0.3637717047
H	-3.8210740150	4.6493742456	3.6122621528
H	-1.3978676356	6.0365969990	2.3748008653
H	0.3149087995	5.6871947959	2.5817184413
H	-0.4875691487	6.8197957946	3.6588194933
H	-1.9163231132	0.2677789890	4.8870512115
H	-0.9907928950	0.5535584281	3.4255286024
H	-2.7122068163	0.9125261401	3.4624568032
H	-0.5907135171	6.3383028824	-1.6133744929
C	-0.2423304229	4.7754437698	-3.0450733799
H	0.0360181566	3.0104156020	-4.2360096163
H	-5.1015618131	2.2987629301	-0.7818724427
N	-3.1698316044	1.4951033627	-0.8415338018
H	-5.4846190159	4.0603967307	1.5797427366
C	3.4563860045	-1.0697005506	-5.7436842556
H	2.1458200952	1.5529875519	-6.0802355418
H	3.8825123957	1.7507083053	-6.0870940756
H	2.8796319405	2.8659324699	-5.1641593930
H	5.1012755370	-2.2995697433	-0.7839228861
C	3.7569579420	-2.9605410408	0.7968397990
H	0.0137837235	5.4562216901	-3.8459432825
C	-3.2427858272	0.6961084401	-1.9757420260
H	3.6182386219	-2.1418294105	-5.6674034973
H	4.2904955782	-0.6411005899	-6.3002870744
H	2.5630628950	-0.9058674899	-6.3479701179
C	4.4564102135	-3.7599493686	1.6861367118
N	2.4646418027	-2.7776561858	1.3020228985
C	-3.0378893796	-0.6775773819	-1.8487575488
C	-3.3899748329	1.2369950042	-3.2511201205
H	5.4842862674	-4.0628799153	1.5763945653
C	3.5897210699	-4.0608046364	2.7406415083
C	2.3646040922	-3.4473667592	2.4931844027
P	1.2829229702	-1.8268934860	0.3711467327
C	-2.9843257701	-1.4746187295	-2.9792474580
N	-3.0101340090	-1.2800884293	-0.5449897215
C	-3.3210188345	0.4466181750	-4.3914448913
H	-3.5235150460	2.3062134926	-3.3426050280
H	3.8209517024	-4.6529445145	3.6087630087
C	1.1835215649	-3.3853817662	3.4065342325
C	0.8971679963	-3.0260005645	-0.9816500898
N	-0.1302137378	-2.4191542641	1.4800994400
H	-2.8585093113	-2.5412919530	-2.8528814139
C	-3.1015740670	-0.9360780638	-4.2561481072
C	-1.8229919328	-1.5154315231	-0.0235401397
C	-3.4564302843	1.0721625024	-5.7430543351
C	0.9601755540	-4.7642422956	4.0701713488
C	1.4868182039	-2.3507292445	4.5336821479
C	-0.0709219888	-2.9602480839	2.7114844523
C	0.9057708493	-4.4005945763	-0.7640458876
C	0.5542233448	-2.5309883939	-2.2375277536
C	-1.4791942339	-2.1108297524	1.1904761262
C	-2.9983146653	-1.8228870316	-5.4550309690
H	-1.0012609312	-1.1967471510	-0.6482801667
H	-3.6180152396	2.1443001364	-5.6663561302
H	-4.2906330234	0.6439689773	-6.2998288098
H	-2.5631399976	0.9083368161	-6.3473928997
H	1.8610805102	-5.0142773061	4.6280036487
H	0.1719115188	-4.6542437992	4.8132685300

```
C    0.6138013081  -5.8864020744   3.1122055377
H    0.6236089157  -2.3352252488   5.1991852621
H    2.3267214843  -2.7380724595   5.1107781359
C    1.7939057273  -0.9508213901   4.0439429241
C   -1.3695151815  -3.0146966043   3.2493844991
H    1.1623770990  -4.7956376097   0.2090436569
C    0.5805549059  -5.2699560337  -1.7948816857
C    0.2290264862  -3.4024072804  -3.2669119841
H    0.5391090539  -1.4648236739  -2.4164869989
C   -2.2367518625  -2.5011419833   2.3090489509
H   -2.1466488151  -1.5505063753  -6.0808340112
H   -3.8833468554  -1.7480892072  -6.0876336692
H   -2.8805142857  -2.8638012732  -5.1653175748
H   -0.3154118901  -5.6895389507   2.5790134547
H    0.4876948706  -6.8226704823   3.6550831159
H    1.3972968319  -6.0386531485   2.3710688651
H    1.9170744592  -0.2714370952   4.8866082413
H    0.9906523641  -0.5561800771   3.4254562827
H    2.7120740456  -0.9152380077   3.4610919481
H   -1.6281937222  -3.3936360539   4.2227085540
H    0.5899311348  -6.3371983060  -1.6178714439
C    0.2422316315  -4.7731909659  -3.0484458616
H   -0.0348026833  -3.0073616968  -4.2383033801
H   -3.2980176196  -2.3930235213   2.4287483578
H   -0.0138727151  -5.4532809767  -3.8499016706
Ga  -4.8059131420  -1.7528275458   0.1100985817
Ga   4.8057931251   1.7530740449   0.1102973292
Cl  -4.8694018181  -3.9366102564   0.4080358459
Cl  -6.2809147078  -1.2019193964  -1.4276186583
Cl  -5.2923475000  -0.6446550964   1.9552152957
Cl   5.2928040359   0.6440010766   1.9547648667
Cl   4.8684209774   3.9366549692   0.4099983040
Cl   6.2807398471   1.2033756801  -1.4279301302
```

```
2GaCl4
  134
G(CENSO)=       -9975.99927132  G(xTB)=        -211.80354158         !CONF27
P    2.2554035850  -1.7956874629   0.5574850315
N    3.6981761044  -2.7843795301   0.2846126147
C    1.7301352833  -2.3598229572   2.2255631926
C    4.9231511312  -2.4716777534   0.8885436763
C    3.8865417349  -3.8534036539  -0.5599360748
C    1.8645741928  -3.6768264714   2.6520631154
C    1.1279870006  -1.4168018542   3.0550847171
C    5.0652965108  -1.4367826865   1.8349176158
C    5.8652870167  -3.3738786860   0.4150287901
C    5.2228757400  -4.2249678849  -0.4866193209
C    2.8444786805  -4.4241404450  -1.4686030912
H    2.3266004945  -4.4117958842   2.0069711302
C    1.4086747975  -4.0448724414   3.9096718915
C    0.6657843059  -1.7897077917   4.3083393798
H    1.0336191555  -0.3901720061   2.7276101773
N    4.0780108375  -0.6539732393   2.1172224233
H    6.0419160660  -1.3392248921   2.3098890260
H    6.9008836815  -3.3959241459   0.7115573175
H    5.6699663459  -5.0347090696  -1.0362497306
C    3.1330250307  -3.9194586549  -2.9187407612
C    2.9378472285  -5.9662453464  -1.4744619644
```

```
C    1.4583821094  -3.9988164081  -1.1098788054
H    1.5213951757  -5.0677687770   4.2432429761
C    0.8088019314  -3.1032395730   4.7383248705
H    0.2022964519  -1.0529564970   4.9505686590
C    4.1136609871   0.1962344656   3.2101875479
H    4.1096408147  -4.3116020615  -3.2033567428
H    2.3972015531  -4.3835069676  -3.5755678270
C    3.1047701905  -2.4146647381  -3.0918552996
H    2.2361977253  -6.3379454713  -2.2196641839
H    3.9269970873  -6.2398109837  -1.8378252728
C    2.6727059929  -6.6271011407  -0.1368049954
N    1.1678501529  -2.8936490456  -0.3803681508
C    0.2578004966  -4.5349759090  -1.5859146067
H    0.4537652520  -3.3937046613   5.7180681826
C    3.4737773407   1.4560380125   3.1932003194
C    4.6817216572  -0.2318383520   4.4177494131
H    3.8525410641  -1.9204617021  -2.4717377595
H    3.3145033464  -2.1526338287  -4.1282137355
H    2.1311274748  -1.9951546978  -2.8453522455
H    1.6733185399  -6.4004186306   0.2316315792
H    2.7522910521  -7.7100805435  -0.2262746010
H    3.3889069336  -6.3062570517   0.6182275712
C   -0.2283281443  -2.6895856718  -0.3881216403
H    0.1680618455  -5.4029121716  -2.2158966775
C   -0.7822752239  -3.7385989950  -1.1292372101
N    2.7825700350   1.9828756375   2.0967291507
C    3.4733532689   2.2133660021   4.3665956753
C    4.6671348204   0.5231382533   5.5783106817
H    5.1066981580  -1.2258983816   4.4472884470
C   -0.7469703211  -1.4623123881   0.0564535743
H   -1.8260905018  -3.8627035514  -1.3515208548
Ga   1.3536634439   3.3698606069   2.2105477241
C    2.9202244692   1.6229243524   0.8334481713
H    3.0075982789   3.1884129318   4.3511452591
C    4.0514184810   1.7877556843   5.5494008181
C    5.2695230323  -0.0127769131   6.8368164677
N   -1.9561380916  -0.9933204647   0.0811139549
H   -0.0073899059  -0.7120925484   0.3265293902
N    1.0416842433   3.0301615940   0.3319001740
Cl   2.2399858640   5.3334673638   2.6381829638
Cl  -0.1238688646   2.8401944182   3.7179651139
C    2.0357694584   2.1562716972  -0.1100619966
H    3.6933789675   0.9357041383   0.5307612630
C    3.9915604821   2.6497274210   6.7690512656
H    6.0607392533   0.6384200555   7.2109042308
H    4.5280767171  -0.0867866549   7.6337958526
H    5.6945801316  -1.0012720339   6.6814967556
C   -3.1539871338  -1.6167105651  -0.2117282166
C    0.3220538643   3.4240107457  -0.7490736282
C    1.9130971476   1.9992048052  -1.4944982972
H    3.4891211103   3.5912026822   6.5611619980
H    3.4540105767   2.1561785721   7.5802284958
H    4.9880410097   2.8763889960   7.1504009024
C   -3.5947690829  -2.8455445080   0.2641426564
C   -4.0222617557  -0.8233552862  -0.9688982342
C   -0.8062687011   4.4299340135  -0.7399062698
C    0.8432407182   2.7918684206  -1.8901271634
H    2.5267228077   1.3653268488  -2.1131204474
H   -2.9343152263  -3.4381510645   0.8807195118
C   -4.8714264129  -3.3082698478  -0.0246279906
```

N	-3.5105620408	0.4217948019	-1.3576249620
C	-5.3022948961	-1.2736642016	-1.2586280219
C	-1.9233587514	3.8811699591	-1.5965689155
C	-0.2789749089	5.7339641053	-1.3952635331
C	-1.3184104156	4.6889066861	0.6890468958
H	0.4460552208	2.8947996043	-2.8855740805
C	-5.7352108477	-2.5141361143	-0.8085715405
C	-5.3234826734	-4.6299711064	0.5071584621
C	-4.0152673369	1.2001593635	-2.3034556425
H	-5.9809075205	-0.6536109167	-1.8260532847
N	-2.5459552317	2.7015609731	-1.3485096067
C	-2.5073840911	4.4392311749	-2.7531856233
H	0.0012100258	5.5021620593	-2.4217071866
H	-1.1029062396	6.4432976982	-1.4529398262
C	0.8967030371	6.3786819531	-0.6928134205
H	-1.5678816577	3.7360124733	1.1491997467
H	-0.5028226083	5.1129067212	1.2737092757
C	-2.5117247415	5.6175030283	0.7871543011
C	-7.1160750919	-2.9834040766	-1.1346823987
H	-5.5989202168	-5.3109634814	-0.2992336606
H	-4.5429743102	-5.1057260820	1.0955534492
H	-6.2063298921	-4.5261942592	1.1392742402
H	-4.7647562656	0.8253803425	-2.9830261318
C	-3.4741680664	2.4703049060	-2.3583354680
C	-3.4688369617	3.5602804588	-3.2355432314
H	-2.2442546342	5.3872681165	-3.1870995053
H	1.2196999773	7.2571741350	-1.2508189515
H	0.6514632553	6.7066592972	0.3143047265
H	1.7485840649	5.7046691836	-0.6230970307
H	-2.8379200024	5.6906195178	1.8237313501
H	-2.2775503527	6.6234433046	0.4439977015
H	-3.3551325556	5.2530447553	0.2032897855
H	-7.6499226658	-2.2507183263	-1.7343789742
H	-7.0958236115	-3.9214848418	-1.6909555306
H	-7.7003890123	-3.1707265171	-0.2327242313
H	-4.0746705484	3.6682831171	-4.1194390596
P	-2.0942788098	1.1403722479	-0.3977695962
C	-3.0539938631	1.4902337534	1.1421247609
C	-4.1517430156	2.3493619181	1.1590404775
C	-2.6446026389	0.8845665019	2.3279672638
H	-4.4838427027	2.8291100529	0.2507723483
C	-4.8284457864	2.5955037190	2.3439824224
C	-3.3274074559	1.1279829774	3.5100312617
H	-1.7961178489	0.2172110838	2.3315149205
H	-5.6790398955	3.2637852746	2.3440855131
C	-4.4190297786	1.9847610228	3.5227198008
H	-2.9965590466	0.6537518177	4.4239620255
H	-4.9490047443	2.1764408124	4.4463755975
Ga	-1.6916389050	-1.2722412404	-4.9639846286
Cl	-0.2686753200	-2.5467763858	-6.0852651396
Cl	-3.3238402991	-2.5575944341	-4.2119050384
Cl	-0.6169120513	-0.2734638704	-3.3176098610
Cl	-2.5533494280	0.2773082665	-6.2921591059

8 References

[1] C. B. Fischer, S. Xu, H. Zipse, *Chem. – Eur. J.* **2006**, *12*, 5779–5784.

[2] J. Surkau, J. Bresien, D. Michalik, A. Schulz, *Angew. Chem. Int. Ed.* **2024**, *202413565*, DOI 10.1002/anie.202413565.

[3] L. Eickhoff, L. Ohms, J. Bresien, A. Villinger, D. Michalik, A. Schulz, *Chem. Eur. J.* **2022**, *28*, e202103983.

[4] G. M. Sheldrick, *Acta Crystallogr. Sect. A Found. Adv.* **2015**, *71*, 3–8.

[5] G. M. Sheldrick, *Acta Crystallogr. Sect. C Struct. Chem.* **2015**, *71*, 3–8.

[6] G. M. G. M. Sheldrick, *SADABS*, University Of Göttingen, Germany, University of Göttingen, Germany, **2004**.

[7] M. J. Frisch, G. W. Trucks, H. B. Schlegel, G. E. Scuseria, M. A. Robb, J. R. Cheeseman, G. Scalmani, V. Barone, B. Mennucci, G. A. Peterson, H. Nakatsuji, M. Caricato, X. Li, H. P. Hratchian, A. F. Izmaylov, J. Bloino, G. Zheng, J. L. Sonnenberg, M. Hada, M. Ehara, K. Toyota, R. Fukuda, J. Hasegawa, M. Ishida, T. Nakajima, Y. Honda, O. Kitao, H. Nakai, T. Vreven, J. A. Montgomery Jr., J. E. Peralta, F. Ogliaro, M. Bearpark, J. J. Heyd, E. Brothers, K. N. Kudin, V. N. Staroverov, T. Keith, R. Kobayashi, J. Normand, K. Raghavachari, A. Rendell, J. C. Burant, S. S. Iyengar, J. Tomasi, M. Cossi, N. Rega, J. M. Millam, M. Klene, J. E. Know, J. B. Cross, V. Bakken, C. Adamo, J. Jaramillo, R. Gomperts, R. E. Stratmann, O. Yazyev, A. J. Austin, R. Cammi, C. Pomelli, J. W. Ochterski, R. L. Martin, K. Morokuma, V. G. Zakrezewski, G. A. Voth, P. Salvador, J. J. Dannenberg, S. Dapprich, A. D. Daniels, O. Farkas, J. B. Foresman, J. V. Ortiz, J. Cioslowski, D. J. Fox, *Gaussian 09, Revision E.01*, Gaussian Inc., Wallingford CT, **2013**.

[8] F. Neese, *Wiley Interdiscip. Rev. Comput. Mol. Sci.* **2022**, e1606.

[9] E. D. Glendening, J. K. Badenhoop, A. E. Reed, J. E. Carpenter, J. A. Bohmann, C. M. Morales, C. R. Landis, F. Weinhold, *NBO 6.0*, Theoretical Chemistry Institute, University Of Wisconsin, Madison, WI, **2013**.

[10] J. E. Carpenter, F. Weinhold, *J. Mol. Struct. THEOCHEM* **1988**, *169*, 41–62.

[11] F. Weinhold, J. E. Carpenter, in *Struct. Small Mol. Ions* (Eds.: R. Naaman, Z. Vager), Springer, Boston, MA, **1988**, pp. 227–236.

[12] F. Weinhold, C. R. Landis, *Valency and Bonding. A Natural Bond Orbital Donor-Acceptor Perspective*, Cambridge University Press, **2005**.

[13] E. D. Glendening, C. R. Landis, F. Weinhold, *NBO 6.0: Natural Bond Orbital Analysis Program*, **2013**.

[14] J. P. Perdew, K. Burke, M. Ernzerhof, *Phys. Rev. Lett.* **1997**, *78*, 1396–1396.

[15] J. P. Perdew, K. Burke, M. Ernzerhof, *Phys. Rev. Lett.* **1996**, *77*, 3865–3868.

[16] S. Grimme, J. Antony, S. Ehrlich, H. Krieg, *J. Chem. Phys.* **2010**, *132*, 154104.

[17] S. Grimme, S. Ehrlich, L. Goerigk, *J. Comput. Chem.* **2011**, *32*, 1456–1465.

[18] F. Weigend, R. Ahlrichs, *Phys. Chem. Chem. Phys.* **2005**, *7*, 3297.

[19] F. Weigend, *Phys. Chem. Chem. Phys.* **2006**, *8*, 1057.

[20] C. Bannwarth, E. Caldeweyher, S. Ehlert, A. Hansen, P. Pracht, J. Seibert, S. Spicher, S. Grimme, *Wiley Interdiscip. Rev. Comput. Mol. Sci.* **2021**, *11*, 1–49.

[21] C. Bannwarth, S. Ehlert, S. Grimme, *J. Chem. Theory Comput.* **2019**, *15*, 1652–1671.

[22] S. Grimme, *J. Chem. Theory Comput.* **2019**, *15*, 2847–2862.

[23] P. Pracht, F. Bohle, S. Grimme, *Phys. Chem. Chem. Phys.* **2020**, *22*, 7169–7192.

[24] S. Grimme, F. Bohle, A. Hansen, P. Pracht, S. Spicher, M. Stahn, *J. Phys. Chem. A* **2021**, *125*, 4039–4054.

[25] S. Grimme, *J. Comput. Chem.* **2006**, *27*, 1787–1799.

[26] C. Adamo, V. Barone, *J. Chem. Phys.* **1999**, *110*, 6158–6170.
